# PRMT1-mediated methylation of ME2 promotes hepatocellular carcinoma growth by inhibiting ubiquitination

**DOI:** 10.1038/s41419-024-07219-y

**Published:** 2024-11-11

**Authors:** Shuai Zhang, Shuling Zhang, Baijuan Xia, Xueying Li, Hongyu Jiang, Su Feng, Yang Xiang, Ya Qiu, Shi Zhou, Peng Luo

**Affiliations:** 1 Department of Nutrition and Food Hygiene, School of Public Health, Guizhou Medical University, 561113 Guiyang, Guizhou P.R. China; 2 Department of Interventional Radiology, the Affiliated Hospital of Guizhou Medical University, 550004 Guiyang, Guizhou P.R. China; 3 School of Basic Medical Sciences, Guizhou Medical University, 561113 Guiyang, Guizhou P.R. China; 4 Department of Radiology, Guiyang Public Health Clinical Center, No.6 Daying Road, Yunyan District, 550001 Guiyang, Guizhou P.R. China; 5 Department of Cancer Research Laboratory, The Affiliated Cancer Hospital of Guizhou Medical University, 550000 Guiyang, Guizhou P.R. China

**Keywords:** Tumour biomarkers, Cancer metabolism

## Abstract

The mitochondrial malic enzyme 2 (ME2), which is frequently elevated during carcinogenesis and may be a target for cancer therapy, catalyzes the conversion of malate to pyruvate. The processes controlling ME2 activity, however, remain largely unclear. In this work, we show that human hepatocellular carcinoma (HCC) tissues contain high levels of ME2 and that the methylation of ME2 stimulates the growth and migration of HCC cells. Furthermore, we observed that ME2 interacts with protein arginine methyltransferase 1 (PRMT1) and that ME2 enzymatic activity is activated by mutation of ME2 at lysine 67. Mitochondrial respiration was markedly increased by activated ME2, which promoted cell division and carcinogenesis. Furthermore, a negative prognosis for patients was strongly linked with the expression levels of PRMT1 and ME2 R67K in HCC tissues. These findings imply that hepatocellular carcinoma growth is aided by PRMT1-mediated ME2 methylation, that is an essential signaling event that cancer cells need to continue mitochondrial respiration.

## Introduction

Primary liver cancer ranks second internationally in terms of cancer-related mortality and is the seventh most prevalent tumor. Its incidence is increasing annually [1]. Globally, more than 906,000 people received a liver cancer diagnosis in 2020. Hepatocellular carcinoma (HCC), which affects approximately 75% of liver cancer patients, is the most common type of liver cancer [2]. The 5-year survival rate for patients with hepatocellular carcinoma remains low despite the availability of a variety of treatment options, such as targeted therapy, radiotherapy, immunotherapy, and surgical resection. This is because HCC progresses quickly and is prone to drug resistance [3]. To improve the clinical prognosis of HCC patients, it is crucial to decipher how key clinical and molecular features influence the patient’s disease course and response to treatment.

Hepatocellular carcinoma is associated with issues related to liver metabolism, as the liver is an organ engaged in metabolism [4]. Cellular metabolic reprogramming has been identified as one of the hallmarks of cancer [5]. As a tumor progresses, tumor metabolism may have an impact on the phenotype of cancer cells and promote their aggressiveness [6]. An increasing body of research indicates that abnormal expression of metabolic enzymes plays a major role in the growth of tumor cells [7, 8]. According to previous studies, excessive hyperglycemia promotes HCC spread through the nuclear translocation of the metabolic enzyme PKM2, which strengthens the immunosuppressive environment [9]. In addition, tumor development is inhibited by TERT dephosphorylation and inhibited by fructose 1,6-bisphosphatase 1 [10].

A class of oxidative decarboxylases known as malic enzymes catalyzes the breakdown of malate into CO2 and pyruvate as well as the generation of the dinucleotide cofactors NAD+ and NADP+, which are necessary for glycolysis, gluconeogenesis, and fatty acid synthesis [11]. The tetrameric protein known as malic enzyme (ME) has a highly conserved amino acid sequence and structure [12]. In mammalian cells, three isoforms of ME have been identified: mitochondrial NADP+-dependent isozyme (ME2), cytoplasmic NADP+-dependent isozyme (ME1), and mitochondrial NADP + -dependent isozyme (ME3). The two primary isoforms of ME are ME1 and ME2 [13]. Among these, ME2 is more prevalent in cells and regulates cellular metabolism more than ME1 or ME3. The carbon skeleton, cellular energy requirements, and reducing equivalents are all factors that ME2 regulates TCA flux for [13, 14]. Furthermore, ME2 markedly improves lipid and glutamine metabolism in cancer cells, satisfying the high metabolic requirements of cancer cells [13]. ME2 is associated with the development of malignancies and is variably expressed in a number of cancers [15–20]. The generation of NADPH and its redox-control ability endow ME2 with powerful oncogenic functions. However, the mechanism of action of ME2 in HCC has rarely been reported.

Histone modifications, noncoding RNA regulation, and DNA methylation make up the majority of epigenetic control. With the rapid development of the field of epigenetics, the functions and mechanisms of cancer-related epigenomes and their associated proteins are better understood [21]. Epigenetic enzymes control epigenomic modifications that are significant drivers of cancer development, proliferation, and invasion [22]. Protein arginine methyltransferases (PRMTs) catalyze the methylation of a range of protein arginine (R) residues, including histones and nonhistone proteins. This activity affects biological functions such cell cycle control, DNA repair, RNA splicing, and transcription [23]. As a prominent member of the protein arginine methyltransferase family, PRMT1 catalyzes the asymmetric dimethylation and monomethylation of arginine side chains in proteins, mostly in the nucleus and cytoplasm [24]. PRMT1 controls protein‒protein interactions by methylating a variety of histone and nonhistone substrates [25]. Phosphoglycerate dehydrogenase (PHGDH) methylation has been linked to serine production and the development of liver cancer, according to a recent study. Despite being downregulated at the mRNA and protein levels, PRMT1-mediated R236 methylation activates PHGDH, promoting serine production, redox homeostasis, and the development of liver cancer [26]. However, the mechanism of action of PRMT1 in HCC has rarely been reported.

In the present study, we demonstrate the important role of ME2 methylation in hepatocellular carcinoma. ME2 is activated by PRMT1-mediated methylation at the R67 position, which promotes redox homeostasis and HCC growth. Moreover, PRMT1-mediated methylation of ME2 inhibits ME2-FBW7 interaction and ubiquitination-mediated degradation, promoting tumor cell survival and proliferation.

## Materials and methods

### Cell lines and clinical samples

Dulbecco’s modified Eagle’s medium (DMEM) supplemented with 10% fetal bovine serum (FBS) and 1% penicillin–streptomycin was used to maintain HepG2, Hep3B, and HEK293T cells at 37 °C and 5% CO2. The Chinese Academy of Sciences Bank of Type Culture Collection provided all of the cells, which were recently verified by short tandem repeat fingerprinting and negative mycoplasma test results. The cells were cultivated in DMEM (US Biological Life Sciences, D9800-03) devoid of serine and glycine and supplemented with 10% dialyzed FBS to deplete serine and glycine. The Affiliated Hospital of Guizhou Medical University was the site of the collection of 73 matched human HCC tissues and noncancer tissues (NCTs). The collection and use of clinical specimens were approved by the Ethics Committee of Guizhou Medical University (approval no. 2023118; Guiyang, China), and each patient provided written informed consent. The International Ethical Guidelines for Biomedical Research Involving Human Subjects and the Declaration of Helsinki were followed in this study.

### Reagents

Antibodies against ME2 (ab126616, 1:1,000 for immunoblotting, 1:100 for IHC staining), GFP (ab1218, 1:2,000; ab32146, 1:5,000), PRMT1 (ab190892, 1:1,000 for immunoblotting, 1:100 for IHC staining), GAPDH (ab8245, 1:500 for immunoblotting), COX IV (ab202554, 1:2,000) and Ki-67 (ab16667, 1:500) were obtained from Abcam. HA (AE036, 1:2,000) and FLAG (AE063, 1:2,000) antibodies were purchased from ABclonal. GST antibody (10000-0-AP, 1:2,000), β-tubulin antibody (11224-1-AP, 1:3,000) and AKT antibody (10176-2-AP, 1:2,000) were purchased from Proteintech. A site-specific antibody recognizing ME2 monomethyl R67 (meR67K-ME2, 1:500 for immunoblotting, 1:50 for IHC staining) was purchased from Biotech Shanghai. L-serine (S4311) and glycine (G8790) were purchased from Millipore-Sigma. AMI-1 (HY-18962) and chloroquine (HY-17589) were acquired from MedChemExpress.

### Oligonucleotides

Small interfering RNAs (siRNAs) of ME2 were obtained from RiboBio (China) with the following sequences:

Si-ME2: 5′-TATGGTTTATTAGTTAAGGGA-3′;

The shRNA targeting sequences were as follows:

human shRNA-PRMT1 #1: 5′-TGAGCGTTCCTAGGCGGTTTC-3′

human shRNA-PRMT1 #2: 5′-GTGTTCCAGTATCTCTGATTA-3

human shRNA-ME2: 5′-AGTTCTTACAGAGCTACTAAA-3′;

### Mass spectrometry analysis

Mass spectrometry (MS) analysis was performed after co-IP to identify the ME2-binding proteins in the cells. In short, HEK293T cells that consistently expressed HA-ME2 were lysed, and IP was subsequently performed using HA beads. SDS‒PAGE was used to separate the immunoprecipitates. Using sequencing-grade trypsin (Promega), the protein bands visible with Coomassie Brilliant Blue staining were removed, and the proteins were subjected to gel digestion. The Q Exactive Plus Orbitrap LC-MS/MS System (Thermo Fisher Scientific) was used to examine the digested samples (HA-ME2-binding proteins), and Proteome Discoverer 1.2 software was used for identification.

### Western blotting

After applying the BCA technique to evaluate the protein content of each sample, samples were subjected to electrophoresis on an SDS‒PAGE gel and then transferred to PVDF membranes (Merck, USA). PVDF membranes were incubated at 4 °C overnight with primary antibodies. The membranes were then washed three times with TBST and incubated for 2 h with secondary antibodies. The ECL reagent (Yeasen, Shanghai, China) was used to identify the protein signals in the membranes. To determine the expression level of the target proteins, GAPDH was used as a loading control

### Coimmunoprecipitation (Co-IP)

To conduct Co-IP experiments, anti-Flag M2 magnetic beads (A2220, Sigma, USA) were incubated for more than 4 h at 4 °C. Following the incubation period, three ice-cold NP40 buffer washes were performed on the beads, and the proteins were eluted in 1× loading buffer by heating to 100 °C for 10 min. Western blotting was then used to examine the eluted proteins.

### Immunohistochemistry (IHC) staining and scoring

Antibodies against PRMT1, ME2, and ME2 R67K were used to stain human HCC tissues. After paraffin-embedded tissues were sectioned at 4 μm, they were incubated overnight at 4 °C with the appropriate antibodies. A GTVision III Detection System/Mo&Rb (Gene Tech, China) was then used according to the manufacturer’s guidelines. In addition, anti-Ki67 and anti-PCNA antibodies were used to stain slices of xenograft tissues fixed in paraffin. Two independent researchers evaluated the staining.

### Cell proliferation and colony formation assays

cells were plated in 96-well plates with 10% FBS in DMEM for the CCK-8 test, and 200 μL of media containing CCK-8 reagent was added to the wells. At 0, 24, 48, 72, and 96 h, the optical density was measured. For the colony formation experiment, HCC cells were seeded in culture dishes at a density of 1000 cells/plate. The clones were preserved, stained, and counted under a microscope after two weeks.

### Cell migration and invasion assays

Transwell chamber assays and cell scratch assays were used to evaluate the potential for cell migration and invasion. Transfected cells (5 × 10^4^ cells) were placed in the upper chamber of the Transwell assay, and 600 μL of 10% serum media was added to the bottom chamber. After 12 h of incubation the cells were stained with 1% crystal violet for 30 min and 5 fields were randomly selected for counting under a 100x microscope. Transfected cells were then digested and counted without matrix gel embedding, and the upper chamber was used for small chamber migration studies. For cell scoring, cells were plated into 6-well plates at a density that resulted in a 100% fusion rate overnight. On Day 2, a 200 μL sterile pipette tip was used to scrape the cells. The cells were scraped and then washed three times with PBS before being incubated in fresh serum-free media. After that, the cells were placed in the incubator and removed at 0 and 48 h. The surface area of the blank was surveyed and measured using ImageJ software. Three replicates of each experiment were performed.

### Immunofluorescence (IF) analyses

The cells were first fixed and then DAPI, secondary antibodies conjugated with fluorescent dye, and primary antibodies at a dilution of 1:100 were added. Confocal microscopy was used to observe and record immunofluorescence images of the cells.

### Expression and purification of recombinant human ME2

The ME2 R67K mutant plasmid was created using a site-directed mutagenesis kit (C215-01, Vazyme, China). *Escherichia coli* BL21 (DE3) cells were used to produce the proteins. One colony was selected, and it was cultivated in LB medium supplemented with ampicillin (100 mg/L) at 37 °C and 200 rpm of shaking. When the cells reached an optical density of 0.4–0.6 at 600 nm (OD600), 400 μmol/L 3-indoleacrylic acid was added to promote protein expression at 18 °C. Following induction for 10 h, the cells were removed and lysed. Following a 30-min centrifugation at 12,000 rpm, the supernatants were separated and incubated at 4 °C for 5 h with His beads. Finally, the bound proteins were subjected to a 2-h elution with 6× His peptide at 4 °C, followed by ultrafiltration.

### ROS measurement

After the cells were washed in PBS, the fluorescent dye 2′,7′-dichlorofluorescein diacetate (H2DCF-DA, MilliporeSigma, 35845) was added, and the cells were incubated at 37 °C for 30 min. The stained cells were trypsinized, washed twice in PBS, and then resuspended in PBS. A Varioskan LUX Multimode Microplate Reader (Thermo Fisher Scientific) was used to measure the fluorescence intensity (Ex. 488 nm, Em. 525 nm) to measure the intracellular ROS levels.

### GSH and GSH/GSSG ratio quantification

A GSH and GSSG Assay Kit (Beyotime, S0053) was used to measure the intracellular GSH level and the GSH/GSSG ratio in accordance with the manufacturer’s instructions. The GSH and GSSG signal intensities were determined with a microplate reader (SpectraMax 190, Molecular Devices) at an optical density of 412 nm.

### NADPH/NADP+ ratio quantification

An NADP+/NADPH Assay Kit (Beyotime, S0179) was used to measure the intracellular NADPH/NADP+ ratio according to the manufacturer’s instructions. At an optical density (OD) of 450 nm, the NADP+ and NADPH signal intensities were determined using a microplate reader (SpectraMax 190, Molecular Devices).

### Measurement of intracellular pyruvate, malate, triglyceride, lactate, and ATP

Using a Pyruvate (PA) Content Assay Kit (BC2205, Solarbio), Malic Acid Content Assay Kit (BC5495, Solarbio), Triglyceride (TG) Content Assay Kit (BC0625, Solarbio), L-Lactic Acid Colorimetric Assay Kit (E-BC-K044-M, Eton), and ATP Content Assay Kit (BC0305, Solarbio), the intracellular pyruvate, malate, triglyceride, lactate, or ATP levels were measured spectrophotometrically in accordance with the manufacturer’s instructions.

### Extracellular acidification rate (ECAR) and oxygen consumption rate (OCR)

The Seahorse XF96 equipment (Seahorse Bioscience, USA) were utilized for the analysis of (OCR). The OCR test utilized assay medium (Seahorse Bioscience) in place of cell media, supplemented with 1 mM pyruvate, 10 mM glucose, and 2 mM glutamine. The test was conducted for 1.5 h at 37 °C, and the results were recorded using the Cell Mito Stress Kit (Seahorse Bioscience). The oligomycin and FCCP concentrations were 1.0 μM and 0.5 μM, respectively. For the ECAR test, cells were cultured for 1.5 h at 37 °C in the assay medium (Seahorse Bioscience) containing 2 mM glutamine. The Glycolytic Stress Test Kit (Seahorse Bioscience) was then used to measure the cells. The Seahorse XF96 Wave program was used to alter the OCR and ECAR results.

### Tumor formation assays in nude mice

At five weeks of age (*n* = 5 for each group), athymic male BALB/c nude mice purchased from Beijing Vital River Laboratory Animal Technology Co. (Beijing, China) were subcutaneously injected with cells. Three weeks after the injection, the mice were sacrificed, and tumor development was assessed. An electronic caliper was used to measure the tumor volume every other day. To reduce the impact of slight variations across groups, the mice were randomly assigned. At the conclusion of the trial, the mice were sacrificed by cervical dislocation, and the main organs and tumors were then removed using surgical scissors. The Guizhou Medical University Animal Care Committee approved the present study (approval no. 2101169).

### Statistics and reproducibility

Every experiment was independently conducted three times with comparable outcomes, and unless otherwise noted, all the data are presented as the means ± SD of three separate trials. To compare the variables between two groups, a two-tailed Student’s t test was used. An association in clinical samples was found using both linear regression and Pearson correlation. A log-rank test and Kaplan‒Meier survival curve were used to assess survival. GraphPad Prism 8.0 was used for all the statistical studies.

## Results

### PRMT1 interacts with ME2

**Fig. 1:**
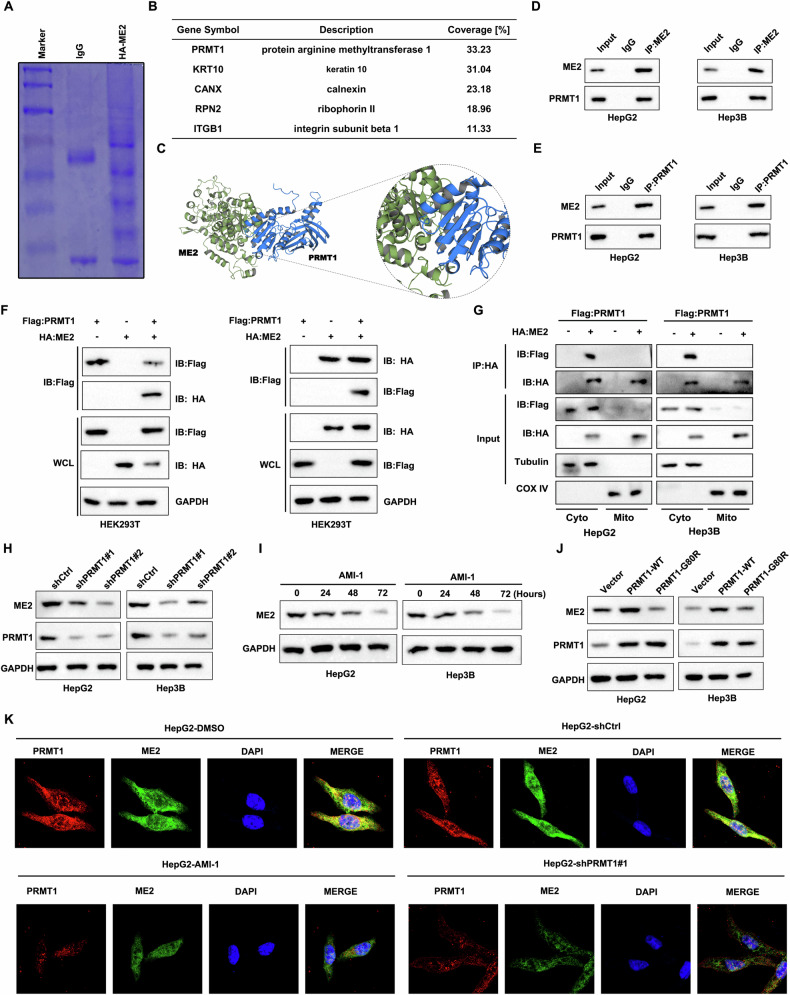
PRMT1 interacts with ME2. **A** Using Coomassie blue staining, we analyzed proteins that interact with ME2. **B** A list of proteins linked to ME2 that were found using mass spectrometry analysis and immunopurification. **C** Analysis of the interaction between PRMT1 and ME2 using protein docking. **D** ME2 binding to PRMT1: an immunoprecipitation study of the protein interactions between ME2 and PRMT1. **E** Protein interactions between ME2 and PRMT1 (PRMT1 binding to ME2) were examined by immunoprecipitation analysis. **F** Immunoprecipitation analysis to examine the exogenous binding relationship between ME2 and PRMT1. Western blotting was used to examine HEK293T cells that had been transfected with Flag-tagged PRMT1 and HA-tagged ME2. **G** Cytoplasmic and mitochondrial fractions of HCC cells transfected with indicated plasmids were immunoprecipitated with an anti-Flag antibody followed by western blotting analysis. β-tubulin and COX IV were used to assess purity of cytosolic and mitochondrial fractions respectively. **H** Immunoblotting assay to analyze the expression of ME2 after interference with PRMT1. **I** Expression of ME2 was analyzed by immunoblotting assay after inhibition of PRMT1 by AMI-1. **J** After mutation of PRMT1 (PRMT1-G80R), ME2 expression was analyzed by immunoblotting. **K** Immunofluorescence assay to analyze the expression level of ME2 after inhibiting or interfering with PRMT1. Scale bars, 5μm.

To elucidate the mechanism of ME2 regulation in tumor cells, potential ME2-interacting proteins were first analyzed using Caulmers Brilliant Blue staining and mass spectrometry, and the protein arginine methyltransferase (PRMT1) was identified (Fig. 1A, B). A protein molecular docking technique was utilized to analyze the mode of action of ME2 and PRMT1 binding to each other (Fig. 1C). Coimmunoprecipitation was subsequently performed to analyze the protein interactions between ME2 and PRMT1, and the results showed that in HCC cells, ME2 can bind to PRMT1 (Fig. 1D, E). The exogenous binding relationship between ME2 and PRMT1 was also analyzed using coimmunoprecipitation (Fig. 1F). Additionally, PRMT1 mainly binds to ME2 in the cytoplasm. (Fig. 1G). Western blotting revealed a significant decrease in the protein expression level of ME2 after interfering with PRMT1 expression (Fig. 1H). In hepatocellular carcinoma cells treated with a PRMT1 inhibitor (AMI-1), the protein expression level of ME2 was significantly downregulated after 72 h of treatment (Fig. 1I). The methyltransferase activity of PRMT1 is impaired by the single amino acid mutation G80R, which was inserted into its conserved methyl donor SAM binding region [27, 28]. In the present study, we generated G80R mutant PRMT1 (PRMT1-G80R) and wild-type PRMT1 (PRMT-WT) strains. Western blotting revealed that ME2 protein expression was upregulated by PRMT-WT but somewhat downregulated by PRMT1-G80R (Fig. 1J). Immunofluorescence analysis revealed that ME2 expression was downregulated following PRMT1 suppression or interference (Fig. 1K). These findings suggest that PRMT1 and ME2 interact.

### ME2 methylation promotes hepatocellular carcinoma cell proliferation and invasive metastatic ability

**Fig. 2:**
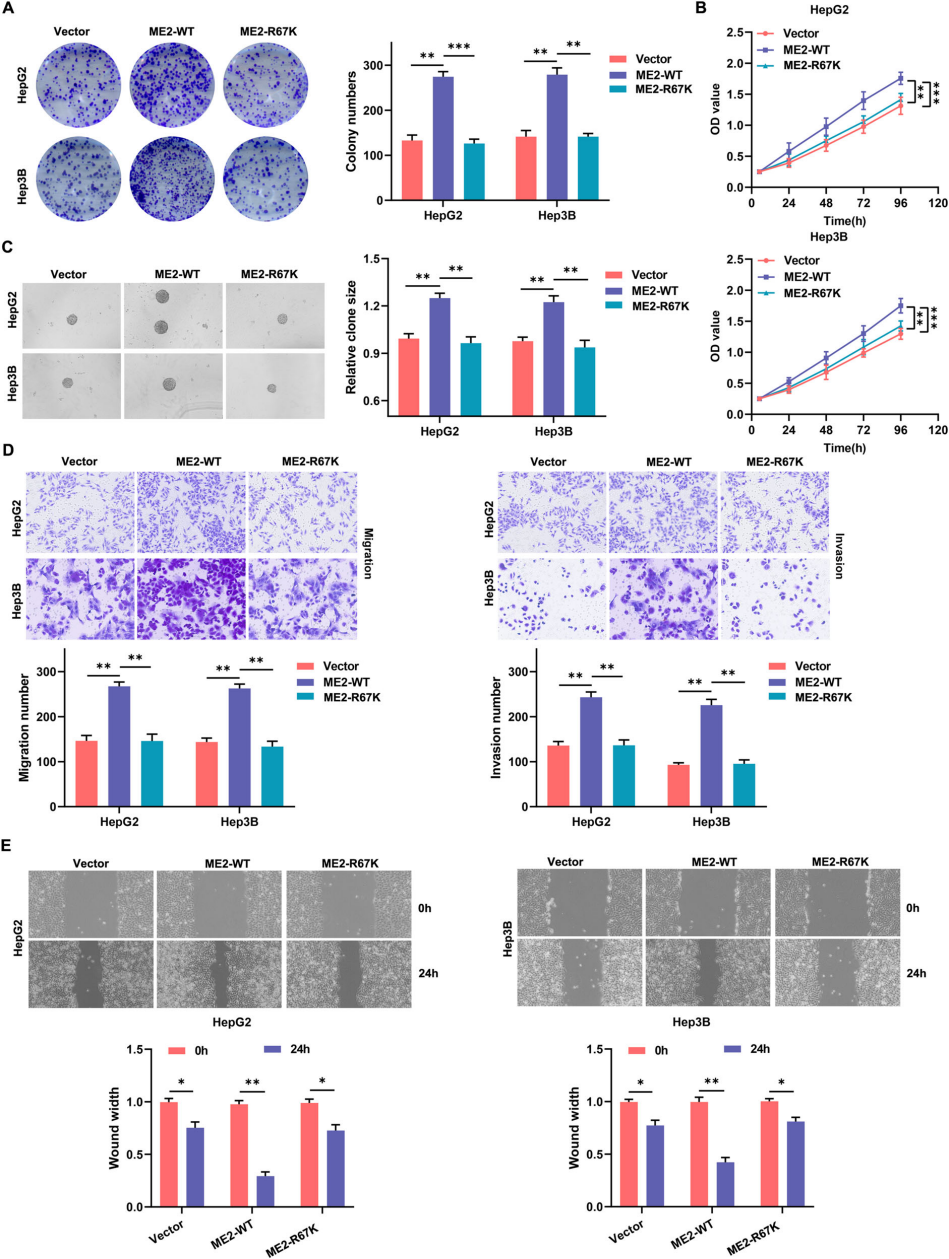
ME2 methylation promotes proliferation and invasive metastasis of HCC cells. **A** Plate cloning assay to analyze the colony forming ability of ME2-WT and ME2-MUT(R67K) on HCC cells. **B** CCK-8 assay to analyze the effects of ME2-WT and ME2-MUT(R67K) on HCC cell activity. **C** Cell suspension culture to analyze the effect of ME2-WT and ME2-MUT(R67K) on the stemness of HCC cells. **D** Transwell migration and invasion assay to analyze the effect of ME2-WT and ME2-MUT(R67K) on the invasive transfer ability of HCC cells. **E** Cell scratch assay to analyze the effect of ME2-WT and ME2-MUT(R67K) on the migration ability of HCC cells. **P < 0.01; ***P < 0.001.

It has been previously reported that arginine 67 (R67) is a methylated residue [29], so we constructed an arginine mutant at the R67 position by mutating R67 to lysine (ME2-R67K) and compared this mutant to ME2 wild-type (ME2-WT) to analyze the effect of ME2 methylation on hepatocellular carcinoma development. A plate cloning assay demonstrated that ME2-WT can enhance the ability of HCC cells to form colonies, whereas ME2-R67K lost this capacity-promoting effect (Fig. 2A). CCK-8 and cell suspension assays demonstrated that ME2-WT promoted the activity and stemness of HCC cells, whereas ME2-R67K lost its ability to promote the activity and stemness of HCC cells (Fig. 2B, C). The results of the Transwell and cell scratch assays demonstrated that ME2-WT stimulated HCC cell invasion and migration, but ME2-R67K did not (Fig. 2D, E). These studies show that ME2 methylation may stimulate HCC cell growth, invasion, and metastasis; however, the impact on hepatocellular carcinoma cells was greatly reduced or eliminated following arginine mutation at the ME2 R67 region.

### PRMT1 promotes hepatocellular carcinoma cell proliferation and invasive metastasis through ME2

**Fig. 3:**
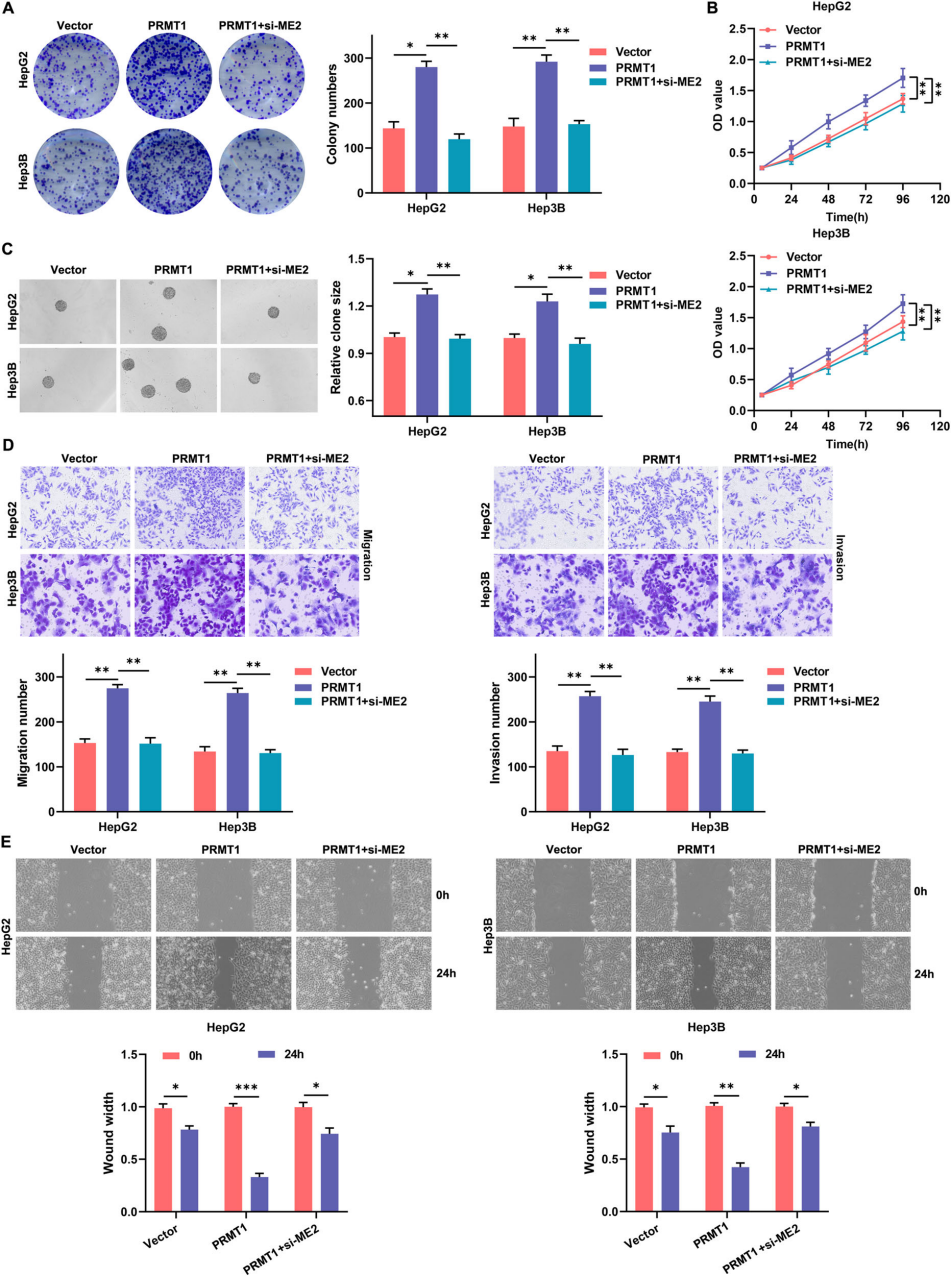
PRMT1 promotes the proliferation and invasive metastasis of HCC cells through ME2. **A** Plate cloning assay to analyze the effect of PRMT1 via ME2 on the colony formation of HCC cells. **B** CCK-8 assay to analyze the effect of PRMT1 on HCC cell activity through ME2. **C** Cell suspension culture to analyze the effect of PRMT1 via ME2 on HCC cell stemness. **D** Transwell migration and invasion assay to analyze the effect of PRMT1 via ME2 on the invasive transfer ability of HCC cells. **E** Cell scratch assay to analyze the effect of PRMT1 on the migration ability of HCC cells via ME2. *P < 0.05; **P < 0.01; ***P < 0.001.

In the previous experiments we observed that in hepatocellular carcinoma, ME2 interacts with PRMT1 and plays a role in the growth and invasive metastasis of HCC cells. Next, we investigated whether the regulation of ME2 by PRMT1 contributes to hepatocellular cancer. We first constructed HCC cells overexpressing PRMT1 (PRMT1) and overexpressing PRMT1 while interfering with ME2 expression (PRMT1+si-ME2). Subsequently, the proliferative viability, cell stemness, and invasive and migratory abilities of the different groups of HCC cells were analyzed. Plate cloning assays, CCK-8 assays and cell suspension assays showed that overexpression of PRMT1 promoted the colony-forming ability (Fig. 3A), cell viability (Fig. 3B) and stemness (Fig. 3C) of HCC cells, whereas overexpression of PRMT1 with concomitant interference of ME2 expression reversed this effect (Fig. 3A–C). Transwell assays and cell scratch assays, on the other hand, showed that overexpression of PRMT1 promoted the ability of HCC cells to migrate and invade, whereas overexpression of PRMT1 combined with ME2 expression similarly reversed the ability of PRMT1 to promote migration and invasion (Fig. 3 D and E). These experiments demonstrate that PRMT1 may facilitate HCC cell invasion, proliferation, and metastasis via ME2.

### ME2-R67 methylation is its functional active site

**Fig. 4:**
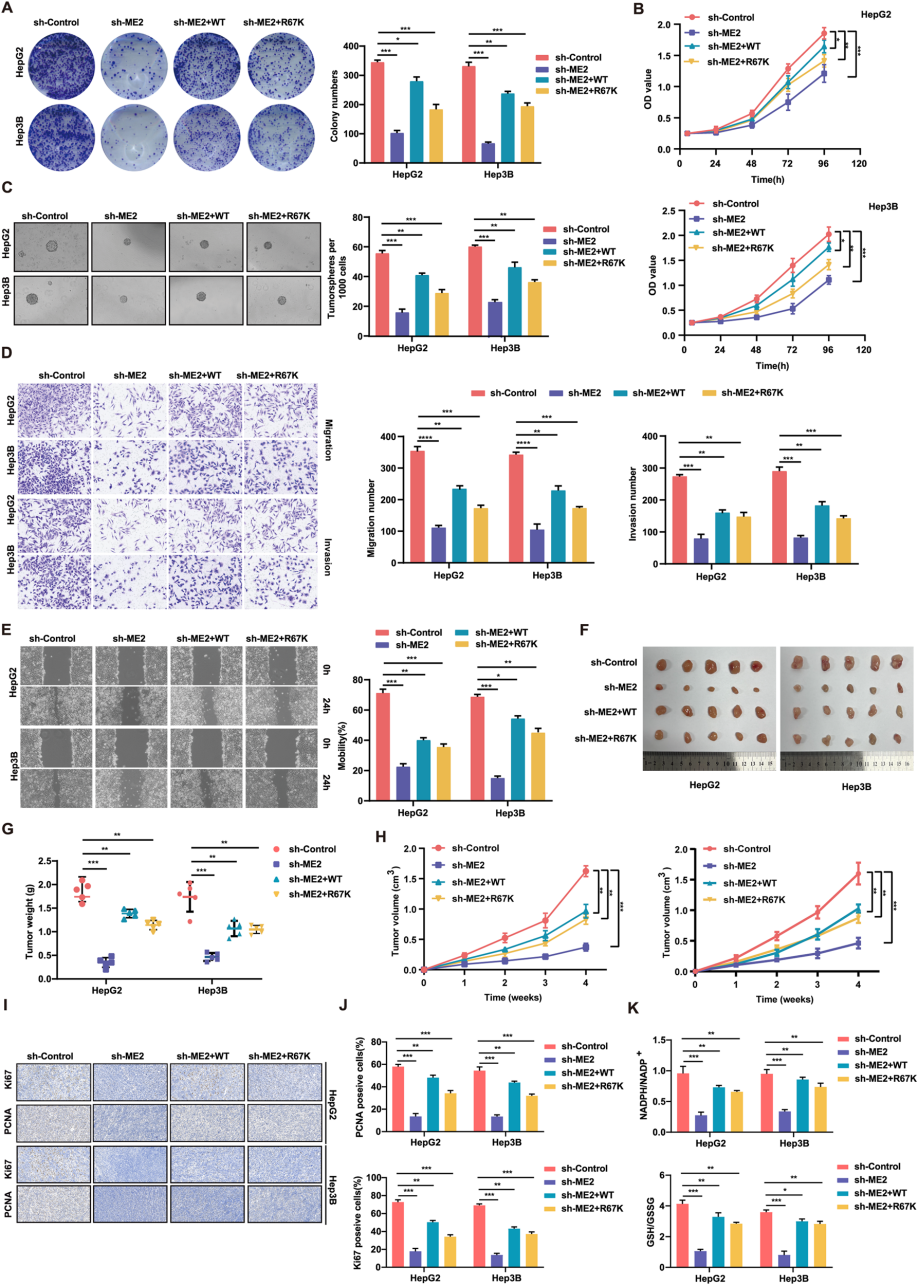
MEK2-R67 methylation is its functional active site. **A** Plate cloning test to examine how ME2 mutation and wild type affect the capacity of HCC cells to form colonies after ME2 knockdown. **B** The CCK-8 experiment to examine how ME2 wild-type and mutation affect the activity of HCC cells after ME2 knockdown. **C** Cell suspension culture analysis of how ME2 wild type and mutation affect the stemness of ME2-knocked down HCC cells. **D** Transwell Migration and Invasion Assay to examine how ME2 wild type and mutation affect invasion and metastasis of HCC cells that have had ME2 knocked down. **E** Cell scratch experiment to examine how the wild type and mutant forms of ME2 affect the capacity of HCC cells to migrate. **F**–**H**. The effects of ME2 wild type and mutant on the tumorigenicity of HCC cells with ME2 knockdown were examined using a subcutaneous tumorigenicity test. Thymus-free nude mice (*n* = 5/group) received subcutaneous injections of HepG2 and Hep3B cells (2 × 10^6^) with varying ME2 status. After 28 days of injection, the mice were sacrificed, and the tumor development was analyzed (**F**). Tumor volume (**H**) and weight (**G**) were assessed. **I**, **J**. To assess the expression and statistical quantification of KI67 and PCNA in the xenograft tumor tissues of each group, immunohistochemical procedures were performed. **K** GSH/GSSG and NADPH/NADP+ ratios in every set of xenograft tumor tissues.

Our studies revealed that the mutation of arginine to lysine at the R67 position of ME2 results in the loss of proliferation and invasive metastasis of HCC cells. To further clarify whether R67 is a functionally active site for ME2 methylation, we constructed HCC cell lines in which ME2 expression was knocked down and analyzed the effects of ME2 wild-type (WT) and R67-mutant (R67K) mutations on HCC cells. Effects of ME2 on the wild type (WT) and R67 mutant (R67K) strains. ME2-wild-type considerably increased the colony-forming ability of HCC cells with suppressed ME2 expression; however, ME2-R67K reduced this effect, according to the plate cloning test data (Fig. 4A). ME2 wild-type significantly promoted the colony formation ability of HCC cells with ME2 knockdown, whereas ME2-R67K significantly promoted the colony formation ability, stemness of the cells, invasive metastasis, and HCC cell activity of HCC cells with ME2 knockdown, according to the results of the CCK-8 assay, cell suspension assay, Transwell assay, and cell scratch assay (Fig. 4B–E). In vivo analysis of the effect of the R67 arginine mutant of ME2 on HCC cells using a nude mouse subcutaneous tumor formation assay and statistical analysis of the weight and volume of subcutaneous tumors were also performed, and the results also demonstrated that the R67 arginine mutant of ME2 in vivo could attenuate the growth-promoting effect of the wild-type ME2 on the growth of HCC cells with knockdown of ME2 expression (Fig. 4F–H). Additionally, proliferation-related indices were measured using immunohistochemistry. The subcutaneous tumor tissues of the sh-ME2-R67K group exhibited considerably lower expression levels of Ki67 and PCNA than did the tumor tissues of the sh-ME2-WT group (Fig. 4I, J). Moreover, the sh-ME2-WT group had the greatest levels of NADPH and GSH, but the sh-ME2-R67K group’s tumor tissues had far lower levels of NADPH and GSH than did the sh-ME2-WT group (Fig. 4K). The above results indicate that arginine at the R67 position is a functional active site for ME2 methylation.

### Role of ME2 methylation in the TCA cycle and NADPH homeostasis

**Fig. 5:**
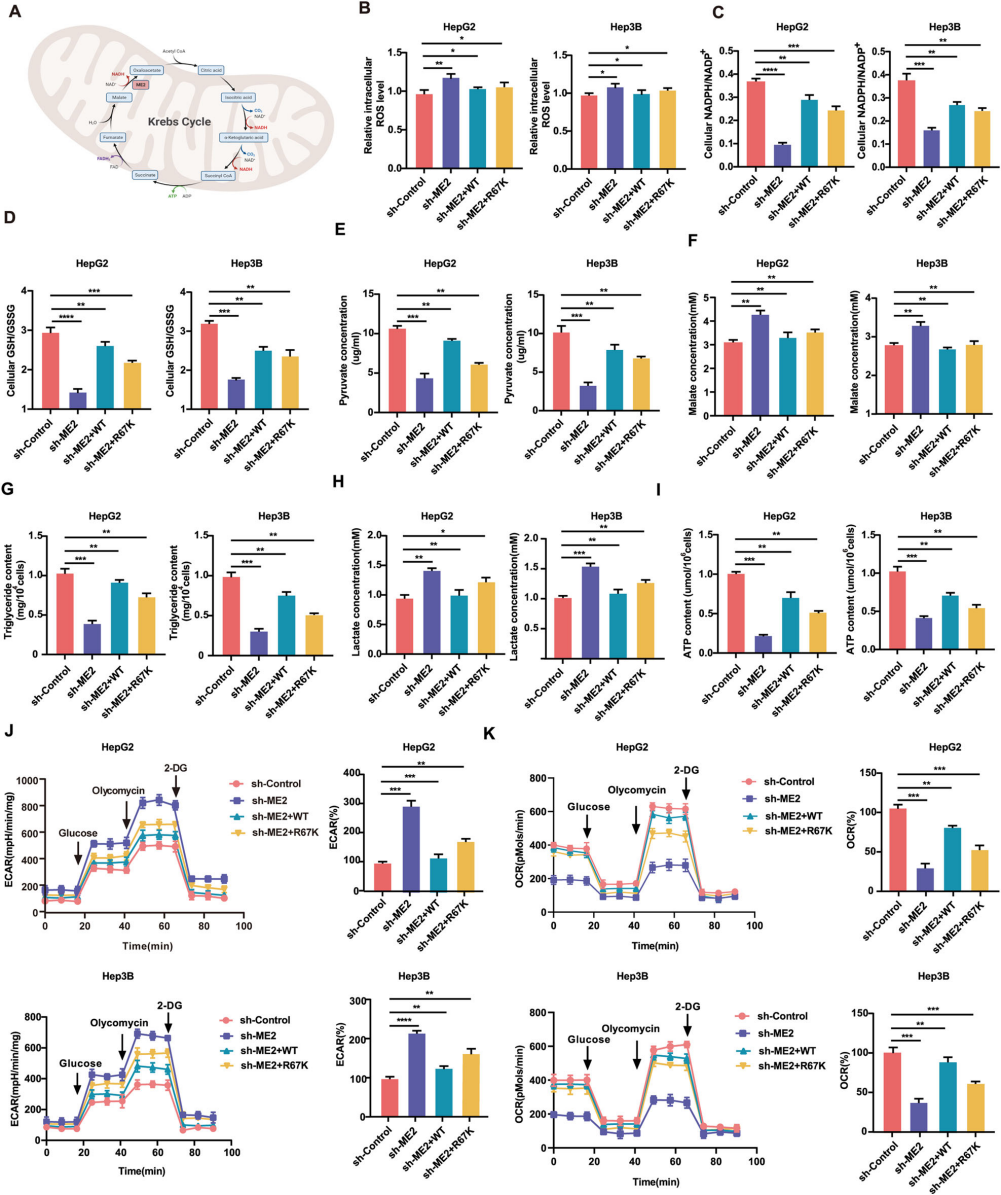
Role of methylation of ME2 in TCA and NADPH homeostasis. **A** Diagram of the biological function of ME2 in the TCA cycle. **B** ROS content analysis of the effects of ME2 wild type and mutation on the level of cellular oxidative stress in HCC cells with knockdown of ME2. **C** Ratio of NADPH/NADP+ in each group of cells. **D** Ratio of intracellular GSH/GSSG in each group of cells. **E**, **F** Pyruvate content in each cell group and Malte content in each cell group. **G** Triglyceride content in each cell group. **H** Lactic acid content in cells of each group. **I** ATP content in each group of cells. **J** EACR analysis of extracellular acidification rate in each group of cells. **K** OCR analysis of oxygen consumption of cells in each group. *P < 0.05; **P < 0.01; ***P < 0.001.

Malic enzymes adjust TCA fluxes to maintain a certain energy balance in the cell and to lower equivalency and biosynthetic precursor needs by recycling malate, a TCA cycle intermediary, to pyruvate (Fig. 5A). We reduced endogenous ME2 and reconstituted it with short hairpin RNA (shRNA)-resistant ME2 WT or ME2 R67K in HCC cells to investigate whether PRMT1-regulated ME2 R67 methylation impacts mitochondrial metabolism and the cellular redox balance. In accordance with previous research [13], loss of ME2 led to an increase in reactive oxygen species (ROS) in the cells (Fig. 5B) and a reduction in the NADPH/NADP+ ratio (Fig. 5C). The recombinant ME2 WT nearly fully reversed these effects (Fig. 5B, C). In accordance with these findings, the GSH/GSSG ratio decreased when ME2 was depleted, and this decrease was reversed when ME2 WT was expressed recombinantly (Fig. 5D). However, compared with cells recombinantly expressing ME2 WT, cells recombinantly expressing ME2 R67K attenuated or lost this reversal (Fig. 5B–D). Similarly, we examined the levels of pyruvate and malate in these recombinant HCC cell lines. Upon knockdown of ME2, pyruvate levels were reduced, malate levels were increased, and recombinant expression of ME2 WT reversed this effect; however, this reversal was lost in cells recombinantly expressing ME2 R67K compared to cells recombinantly expressing ME2 WT (Fig. 5E, F).

We also measured triglyceride levels since ME2 is associated with lipid metabolism [13, 30]. We found that individuals with lower endogenous ME2 levels also had lower triglyceride levels (Fig. 5G). Our results show that ME2 activity is increased by arginine methylation at the R67 location, which regulates the quantities of related metabolites and helps to maintain cellular redox balance. Given that ME2 is a crucial TCA cycle regulating enzyme, we measured the levels of lactate, ATP, and OCR as well as the extracellular acidification rate (ECAR) to further investigate the function of ME2 methylation in coordinating the metabolism of HCC cells (Fig. 5H–K). Increased glycolysis and decreased mitochondrial respiration resulted following ME2 knockdown. ME2 WT decreased glycolysis levels and improved the capacity to recover compromised mitochondrial respiration, but this effect was not observed in cells with ME2 R67K (Fig. 5J, K). Similarly, ME2 silencing led to decreased ATP levels and increased lactate levels, which were reversed in ME2 WT but not in ME2 R67 (Fig. 5H, I). Taken together, our findings indicate that in HCC cells, ME2 triggered by R67 methylation sustains mitochondrial respiration.

### Inhibition of ME2 ubiquitination by PRMT1 increases ME2 protein stability

**Fig. 6:**
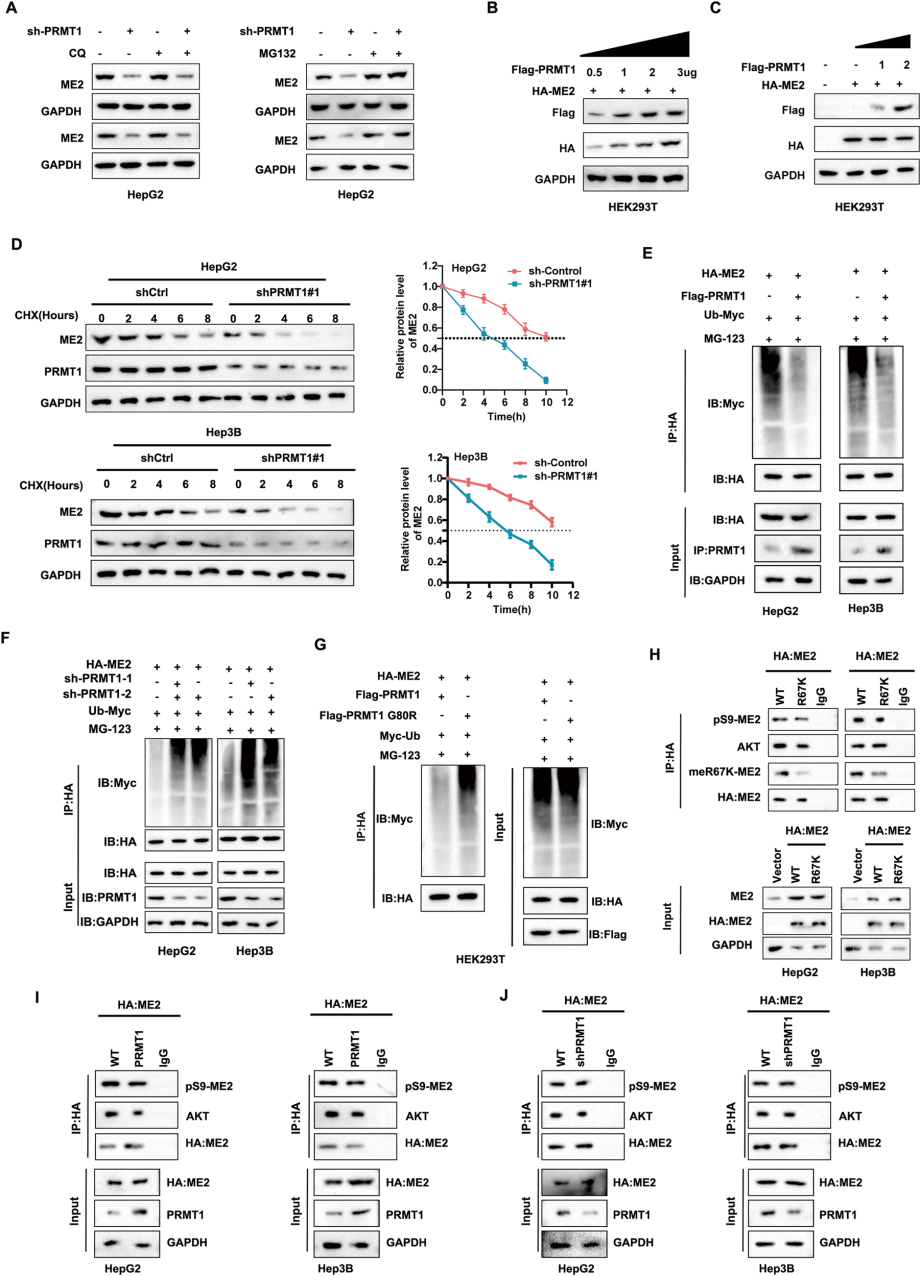
Inhibition of ubiquitination of ME2 by PRMT1 mediates the upregulation of its protein stability. **A** The effect of PRMT1 on the upregulation of ME2 expression was analyzed after the addition of chloroquine (lysosomal inhibitor) and MG132 (proteasome inhibitor) to cells. **B** ME2 expression was detected by immunoblotting after transfection with different doses of PRMT1. **C** ME2 expression was detected by immunoblotting after transfection with PRMT1 for different times. **D** Protein stability analysis of ME2 expression at different time points after addition of CHX to cells. **E** Ubiquitination assay to detect the ubiquitination level of ME2 after overexpression of PRMT1. **F** Ubiquitination assay to detect the ubiquitination level of ME2 in cells after disruption of PRMT1. **G** Ubiquitination assay to detect the ubiquitination level of ME2 in cells after mutation of PRMT1. **H** The changes of pS9-ME2 and meR67K-ME2 by IP, and the interaction between ME2 and AKT were detected both in HepG2 cells and Hep3B cells when wild and mutant HA-ME2 (WT, R67K) were transiently overexpressed. **I**, **J**. The amount of pS9-ME2 and the ME2-AKT interaction were detected by IP HA-ME2 after over-expressing PRMT1 (I) or silencing PRMT1 (J) in HepG2 cells and Hep3B cells, respectively.

We have shown in our study that PRMT1 enhances HCC cell proliferation and invasive metastatic potential through ME2. The impact of PRMT1 on the upregulation of ME2 expression was examined following the addition of MG132, a proteasome inhibitor, and chloroquine, a lysosomal inhibitor, to the cells to better elucidate the mechanism by which PRMT1 controls ME2 in HCC, and Western blotting showed that chloroquine and MG132 reversed the decrease in ME2 levels induced by PRMT1 knockdown (Fig. 6A). In addition, detection of ME2 expression levels at different doses and different transfection times revealed that ME2 expression levels were controlled by the dose of PRMT1 (Fig. 6B, C). Further analysis of the protein stability of ME2 by detecting ME2 expression at various time points following the addition of the protein synthesis inhibitor cycloheximide (CHX) to the cells revealed that PRMT1 was able to maintain the stability of ME2 (Fig. 6D). These findings suggest that PRMT1 regulates ME2 stability through proteasome activity. These findings are supported by the finding that PRMT1 upregulation reduced ME2 ubiquitination PRMT1 downregulation increased ME2 ubiquitination, whereas PRMT1 downregulation increased ME2 ubiquitination (Fig. 6E, F). However, when the location at which PRMT1 binds to ME2 was altered, the increase in protein expression levels and decrease in ME2 ubiquitination induced by PRMT1 overexpression was not observed (Fig. 6G). Recent research has demonstrated that ME2 can reside in the cytoplasm, where it is subject to phosphorylation by AKT at serine 9 within the N-terminal region, a post-translational modification that redirects cellular metabolism towards glycolysis [11]. In light of these findings, we conducted a study in which we transiently transfected HepG2 and Hep3B cells with plasmids encoding wild-type and mutant HA-ME2 (WT and R67K). Following this, we evaluated the levels of pS9-ME2 by HA-ME2 immunoprecipitation in both the WT and R67K groups. Our findings indicated that, in comparison to the WT group, the expression levels of meR67K-ME2 were reduced in the R67K mutant group, whereas the expression levels of pS9-ME2 exhibited no significant variation (Fig. 6H). Moreover, we observed that the interaction between ME2 and AKT was not significantly altered after the R67K-ME2 mutation (Fig. 6H). Additionally, we scrutinized the expression of pS9-ME2 by HA-ME2 immunoprecipitation subsequent to the overexpression or knockdown of PRMT1. Our data revealed that neither the interaction levels of AKT-ME2 nor the levels of ME2-S9 phosphorylation exhibited significant alterations (Fig. 6I, J). The above experimental results suggest that PRMT1 can increase the protein stability of ME2 by inhibiting its ubiquitination.

### PRMT1 mediates methylation of ME2 to inhibit ME2-FBW7 interaction and ubiquitination

**Fig. 7:**
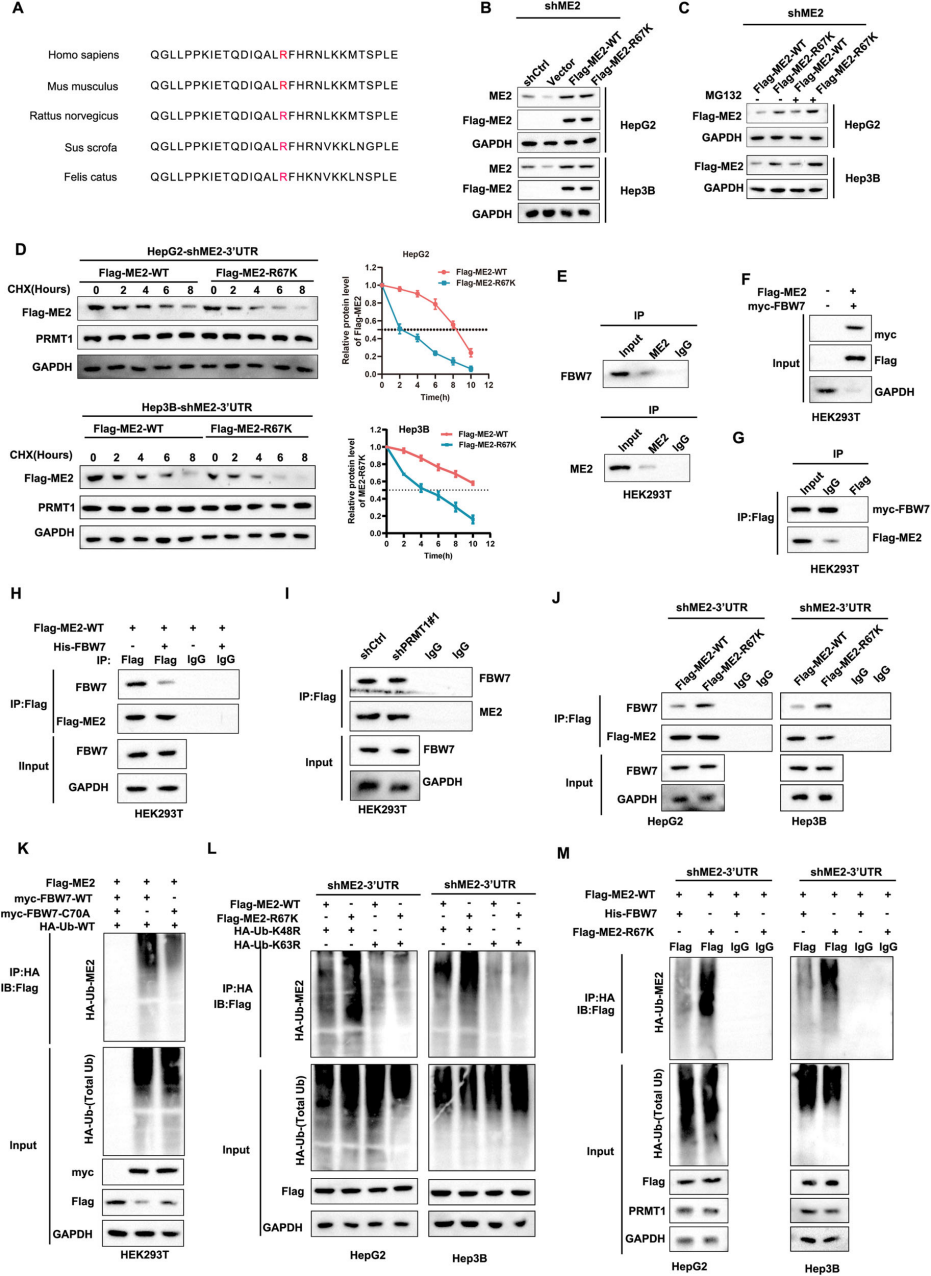
PRMT1-mediated methylation of ME2 inhibits ME2-FBW7 interactions and ubiquitination. **A** Conserved methylation sites of ME2 were analyzed in multiple species. **B, C** Analysis of the expression levels of ME2 in wild type and mutants. **D** Analysis of changes in protein stability of ME2 wild-type and mutants. **E** Immunoprecipitation assay to analyze the interaction of ME2 with its E3 ubiquitination ligase FBW7. **F**, **G** Exogenous immunoprecipitation assay to analyze the interaction of FBW7 with ME2. **H** Exogenous immunoprecipitation assay to analyze PRMT1 inhibition of FBW7 interactions with ME2. **I** Endogenous immunoprecipitation analysis of PRMT1 inhibits the interaction between FBW7 and ME2. **J** Methylation of ME2 inhibits the interaction between FBW7 and ME2. **K** FBW7 promotes ubiquitination of ME2 as analyzed by ubiquitination assay. **L** Ubiquitination assay analysis showing that FBW7 can promote the ubiquitination level of ME2-K48. **M** Ubiquitination assay analysis showing that FBW7 modifies ubiquitination of wild-type and methylated ME2.

R67 of ME2 is a residue that is conserved across species according to sequence comparison (Fig. 7A). Analysis of ME2-WT and ME2-R67K in ME2-depleted HCC cells revealed increased ME2 expression (Fig. 7B). Moreover, ME2-R67K may regulate ME2 stability through proteasome-mediated mechanisms, as evidenced by the increase in ME2 expression when the proteasome inhibitor MG132 was present (Fig. 7C). Moreover, after the protein synthesis inhibitor cycloheximide (CHX) was added to the cells, protein stability was analyzed by detecting the expression of ME2 at different time points, and the results showed that ME2 stability was poor in the ME2-R67K group (Fig. 7D). Mass spectrometry revealed that ME2 binds to FBW7, which is an E3 ubiquitination ligase [31]. An investigation into the potential involvement of ubiquitination in the pathogenesis of HCC involved the use of a coimmunoprecipitation test, which demonstrated the interaction between ME2 and FBW7 (Fig. 7E). Similarly, the results of exogenous co-IP showed that FBW7 can interact with ME2 (Fig. 7F, G). Further analysis revealed that PRMT1 inhibited the interaction between FBW7 and ME2 (Fig. 7H, I). Moreover, the methylation of ME2 inhibited the interaction between FBW7 and ME2 (Fig. 7J). Ubiquitination experiments demonstrated that FBW7 can facilitate the ubiquitination of ME2, but the E3 ligase-inactivated mutant FBW7-C70A was unable to catalyze the ubiquitination of ME2 (Fig. 7K). The degradation of proteins in the proteasome pathway is mostly facilitated by ubiquitination of K48, whereas signal transmission and protein stability are the major functions of K63-linked ubiquitination. To elucidate the type of ubiquitin linkage of ME2, endogenous IP experiments revealed that K48-linked ubiquitination predominated on ME2, with no evidence of K63-linked ubiquitination (Fig. 7L). Moreover, FBW7 promoted the ubiquitination of ME2-R67K (Fig. 7M). The above experimental results indicate that PRMT1-mediated methylation of ME2 inhibits ME2-FBW7 interactions and decreases its ubiquitination.

### PRMT1 is highly expressed in HCC and is positively correlated with ME2 methylation and poor clinical prognosis in hepatocellular carcinoma patients

**Fig. 8:**
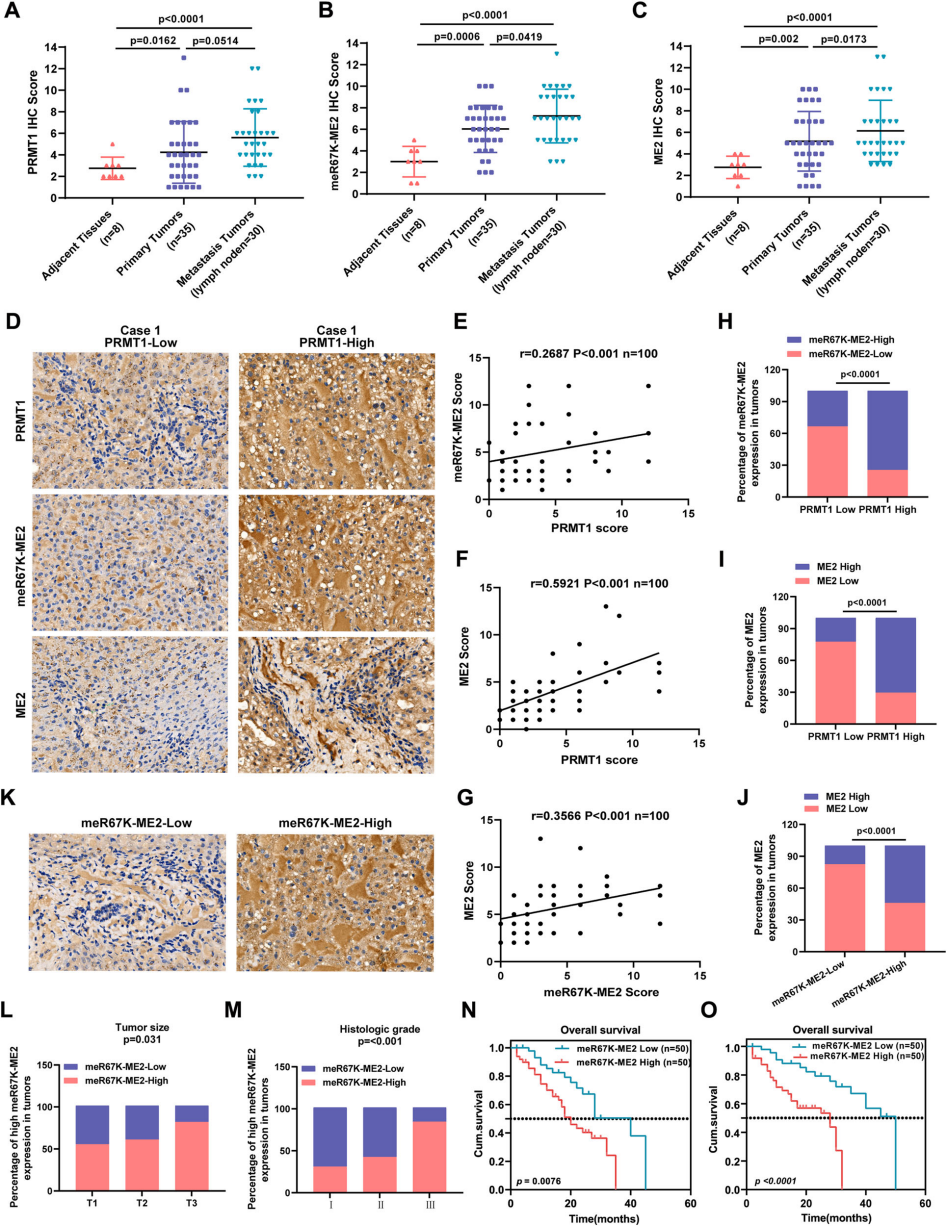
Correlation of PRMT1 expression with ME2 methylation and clinical prognosis of HCC. **A**–**C** Immunohistochemical staining assay of the methylation levels of PRMT1 (A), ME2 (B), and ME2 expression in 73 paired human paracarcinomas, primary foci, and metastatic foci. **D**, **K** Images of immunohistochemical analysis of the expression levels of PRMT1, ME2, and ME2-R67K. **E**–**G** Correlation of PRMT1 (**E**), ME2 (**F**), ME2-R67K (**G**) in HCC tissues analyzed after immunohistochemical scoring. **H**–**J**. Cardinality analysis of expression correlation of PRMT1 (**H**), ME2 (**I**), ME2-R67K (**J**) after post immunohistochemical scoring. **L**, **M** Cardinality analysis of correlation between ME2-R67K expression and tumor size (**L**) and tumor pathological grade (**M**). **N**, **O** Survival analysis of association between ME2-R67K expression and prognosis of HCC patients.

To determine the clinical importance of PRMT1 and ME2-R67 methylation, immunohistochemical studies of 73 HCC samples and surrounding paracancerous normal tissues were performed. The findings demonstrated that the HCC tissues had considerably higher levels of PRMT1, meR67K, ME2, and ME2 than the nearby normal tissues (Fig. 8A–C). There was a significant correlation observed between the expression levels of PRMT1, meR67, ME2, and ME2 in HCC tissues (Fig. 8D–J). Tumor size, tumor pathology grade, and ME2-R67K expression were also found to be positively correlated with each other (Fig. 8L, M). The expression of ME2-R67K was strongly associated with a poor prognosis for individuals with hepatocellular carcinoma according to survival analysis (Fig. 8N, O).

## Discussion

One of the “hallmarks of cancer” is the reprogrammed metabolic signature, which is the molecular fingerprint of cancer cells [32, 33]. An abnormal pattern of enzyme machinery expression coordinates metabolic processes into a framework that ultimately supports cell division, survival, and growth. The role of the NADP(+)-dependent mitochondrial malic enzyme 2 (ME2) in supplying the cell with pyruvate and reducing power for fatty acid and nucleotide production as well as maintaining redox equilibrium has attracted much interest [18]. According to recent research, In the absence of PTEN, AKT1 is activated, and the activated AKT1 phosphorylates ME2fl at the Ser9 site, resulting in the inability of ME2fl to localize to the mitochondria and promote the TCA cycle. The phosphorylated ME2fl will localize to the cytoplasm and form a complex with glycolysis-related enzymes, which leads to a shift in cellular metabolism from oxidative phosphorylation to glycolysis, promoting tumor proliferation [11]. However, a number of malignancies have been linked to the involvement of ME2 in their development. ME2 was highly expressed in CRC tissues, and knockdown of ME2 inhibited CRC cell proliferation. Glutamine deficiency enhanced the interaction of SIRT5 with ME2, which promoted SIRT5-mediated desuccinylation of the ME2 K346 site and activated the enzymatic activity of ME2. Activated ME2 significantly enhanced mitochondrial respiration to counteract glutamine deficiency, promoting cell proliferation and tumor development. In addition, the succinylation level of ME2 K346 in CRC tissues was negatively correlated with the level of SIRT5 and correlated with patient prognosis.SIRT5-mediated desuccinylation of ME2 is a key signaling event for cancer cells to maintain mitochondrial respiration under glutamine-deficient conditions and to promote CRC progression, offering the possibility of targeting the SIRT5-ME2 axis for the treatment of CRC [34]. However, little is known about the functional regulatory mechanisms of ME2 in hepatocellular carcinoma. In this study, we found that ME2 and PRMT1 can interact with each other and that ME2 can promote the proliferation and invasion of HCC cells; however, when arginine 67 is mutated to lysine, the promoting effect of ME2 on HCC cells is reversed. Moreover, we found that PRMT1 promoted the proliferation and invasion of HCC cells through ME2.

Growing evidence links protein arginine methylation to the development of cancer, at least partially by controlling metabolic reprogramming. Previous research has revealed that in glioblastoma stem cells, PRMT6 methylation of RCC1 controls mitogenesis, tumorigenicity, and radiation sensitivity [35]. Type I PRMT inhibitors attenuate PARPi resistance and thus may have an important clinical impact on non-small cell lung cancer treatment [36]. Additionally, PRMT4 limits the metabolism of glutamine in pancreatic ductal adenocarcinoma by blocking the activity of methylated malate dehydrogenase 1 (MDH1) or methylated glyceraldehyde-3-phosphate dehydrogenase, both of which hinder the development and multiplication of cancer cells in HCC glycolysis [37, 38]. However, the mechanism of action of PRMT1 in HCC is unclear. Since altered protein interactions are usually associated with posttranscriptional modification of proteins [39], we further explored whether the response of HCC cells to PRMT1 was related to the posttranscriptional modification of ME2, and observed that arginine at position R67 is a functionally active site for ME2 methylation. Moreover, PRMT1 was able to regulate ME2 methylation to promote the proliferation and invasive metastatic ability of HCC cells.

While the Warburg effect and aerobic glycolysis are widely recognized as key components of cancer cell metabolism, an increasing amount of research indicates that mitochondrial metabolism is responsible for more than just producing energy. These functions include the generation of reactive oxygen species (ROS), biosynthesis, and control of cellular growth and death, all of which are intimately related to the development of tumors [40, 41]. As a fumarate sensor in tumor cells, ME2 may dimerize in response to elevated fumarate concentrations, increasing its enzymatic activity and encouraging mitochondrial production and cell division [42]. In this study, we observed that ME2 knockdown markedly increased lactate and ECAR levels while decreasing OCR and ATP levels. Increasing the levels of ROS also increased the GSH/GSSG and NADPH/NADP+ ratios. ME2-R67K eliminated the reversal impact shown by ME2-WT following alteration of ME2 expression. This finding demonstrates the critical function of the PRMT1-ME2 axis in preserving redox homeostasis and mitigating oxidative stress in cancerous cells.

Protein stability is regulated by the important posttranslational modification process of ubiquitination, which is followed by proteasomal degradation of ubiquitinated proteins [43, 44]. Human chromosome 4, q31 (4q31), contains the gene encoding FBW7, also known as FBXW7. A number of cancers, including pancreatic, ovarian, non-small-cell lung, and stomach cancers, have mutations in or lack this gene [31, 45–48]. A component of the Skp1-Cullin-F-box (SCF) ubiquitin ligase complex, FBW7, is a substrate-recognizing member of the F-box protein family [49]. c-Myc, Cyclin E, c-Jun, Notch1, and mTOR are among the oncogenic proteins that specifically facilitate ubiquitination and proteasomal destruction, making them moniker cancer suppressors. Phenotypic activation of FBW7 expression can be facilitated by p53, and the two proteins work together to preserve genomic integrity and inhibit the growth of tumors [50]. In the present study, we found that FBW7 interacts with ME2 using mass spectrometry, while PRMT1 interfered with the interaction of ME2 with FBW7 by mediating the methylation of ME2, inhibiting the ubiquitination of ME2 and consequently its subsequent ubiquitination-mediated degradation. Most ME2 ubiquitination, according to our analysis, was K48-linked; K63-linked ubiquitination was not detected. Protein stability and signal transduction are the primary functions of K63-linked ubiquitination, whereas proteasomal pathway degradation is the primary function of K48-linked ubiquitination [51], which is consistent with our results. The clinical connection between the tumor and surrounding normal tissues of 73 HCC patients revealed that PRMT1 was strongly expressed in HCC and strongly linked with both ME2 methylation and poor clinical prognosis [51], which is consistent with our results. Moreover, according to the clinical correlation between tumor tissues and adjacent normal tissues of 73 HCC patients, PRMT1 was highly expressed in HCC and was positively correlated with ME2 methylation and poor clinical prognosis in hepatocellular carcinoma patients.

In this work, for the first time, we investigated the mechanism of ME2 methylation and observed that PRMT1 mediates the methylation of ME2 and prevents its ubiquitination, which increases its protein stability and enhances mitochondrial respiration and HCC tumor development. ME2 R67 methylation is linked to the clinical outcome of HCC patients. Therefore, targeting the PRMT1-ME2 axis may be a novel approach for the treatment of HCC.

## Supplementary information

supplementary material

Reproducibility checklist

## Data Availability

The data supporting the findings of this study are available from the corresponding author upon reasonable request.
