# Supplementary figures and images for: PRMT1-mediated methylation of ME2 promotes hepatocellular carcinoma growth by inhibiting ubiquitination
Source: Cell Death Dis. 2024 Nov 11;15(11):814. doi: 10.1038/s41419-024-07219-y (PMC11555414; doi:10.1038/s41419-024-07219-y)

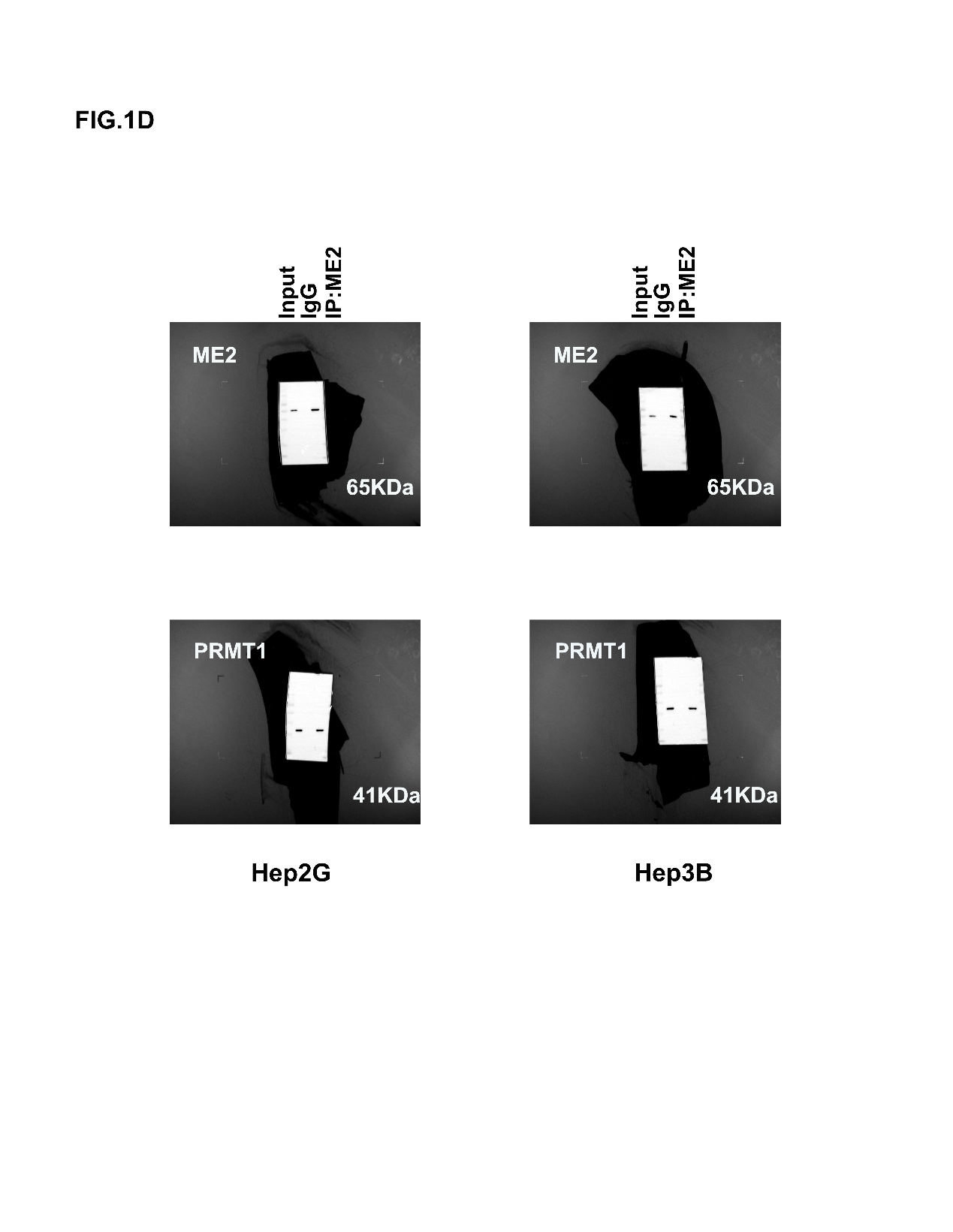


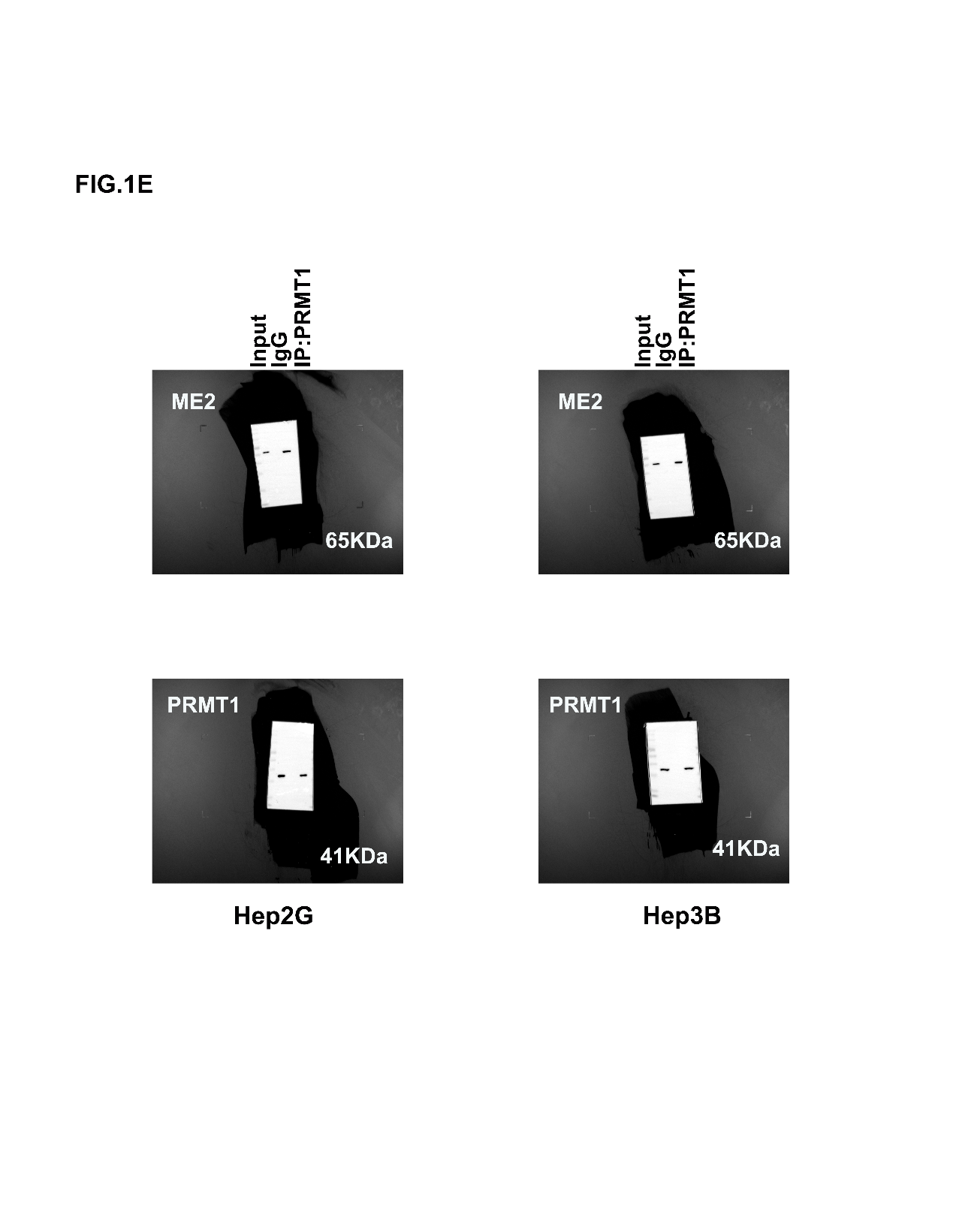


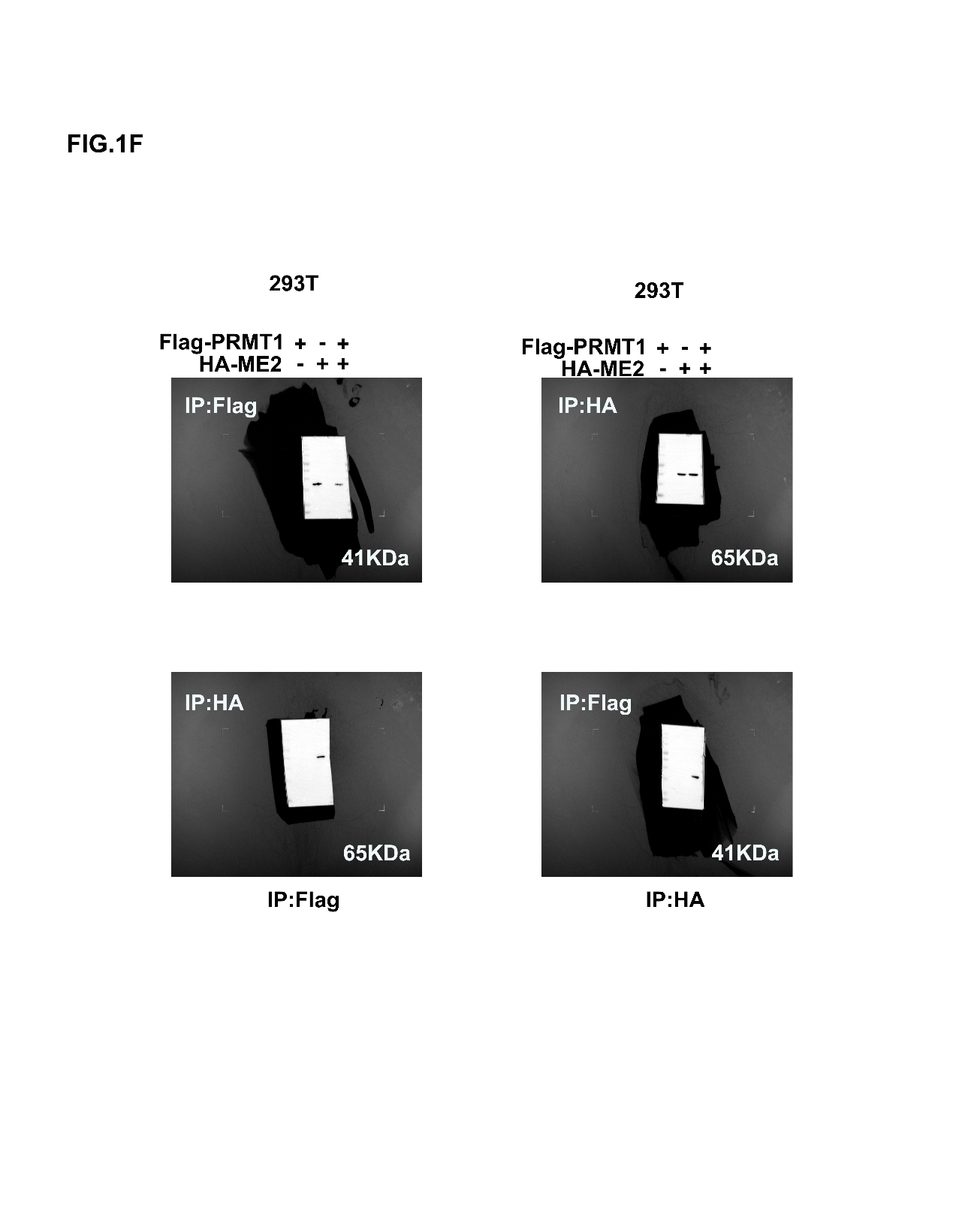


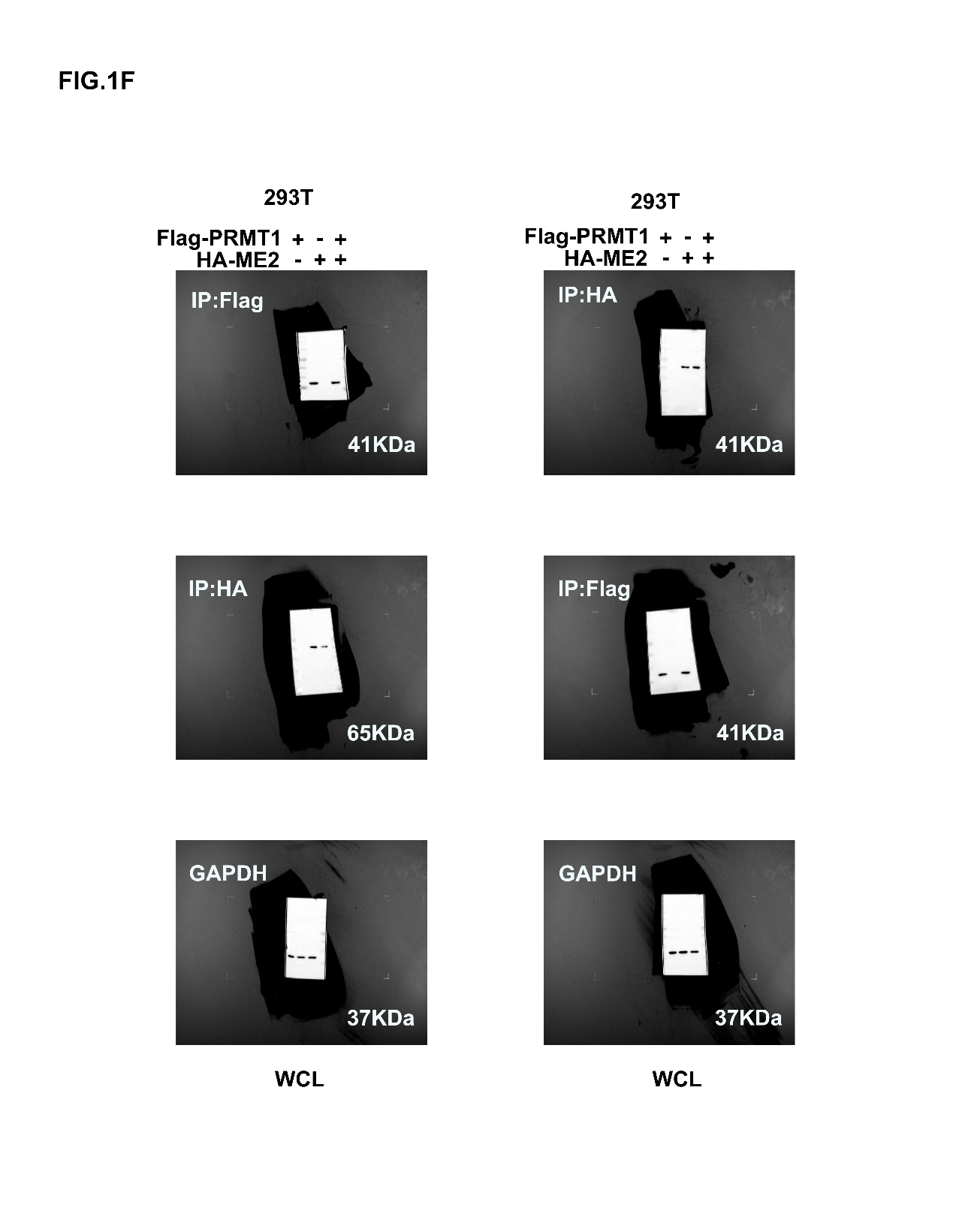


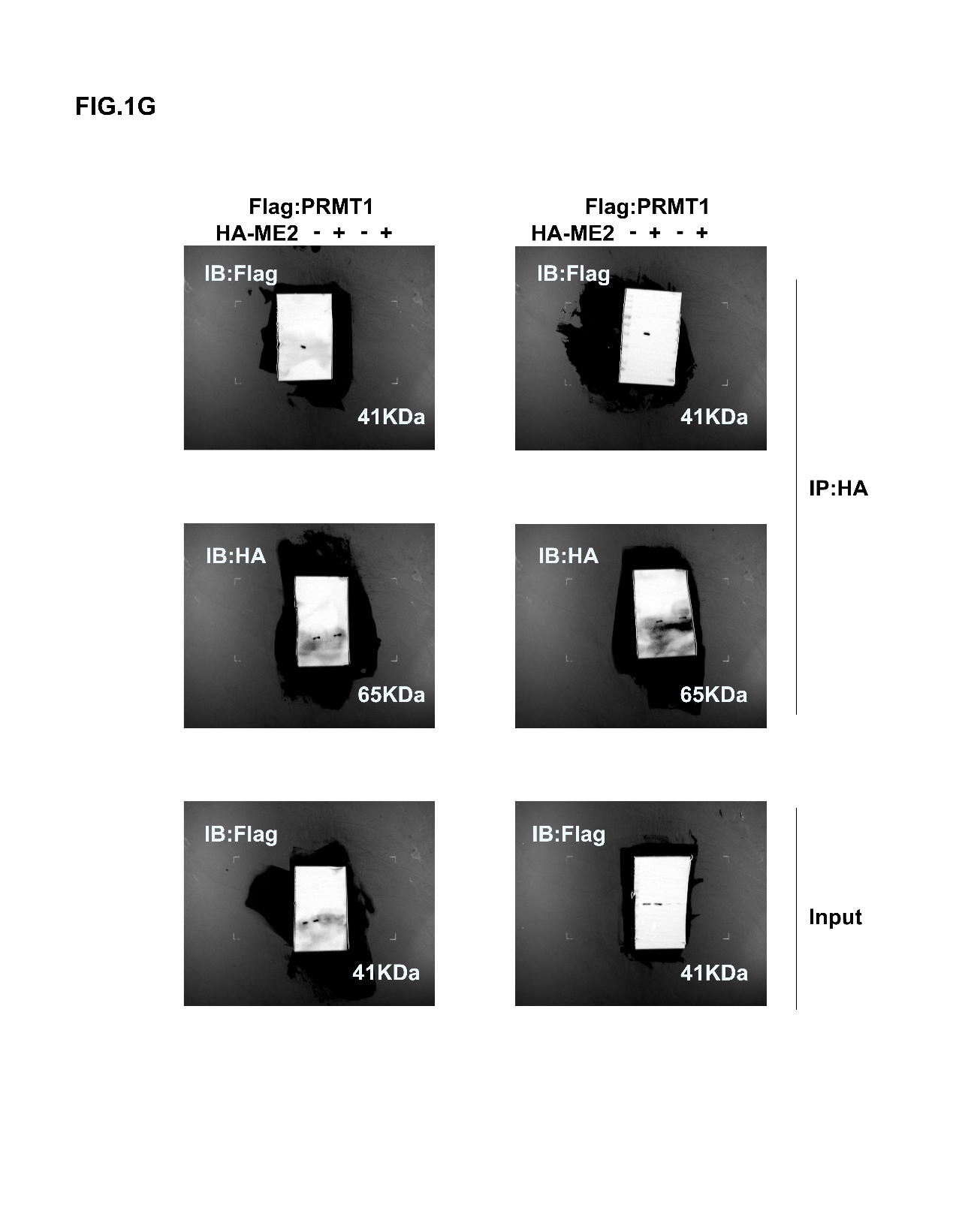


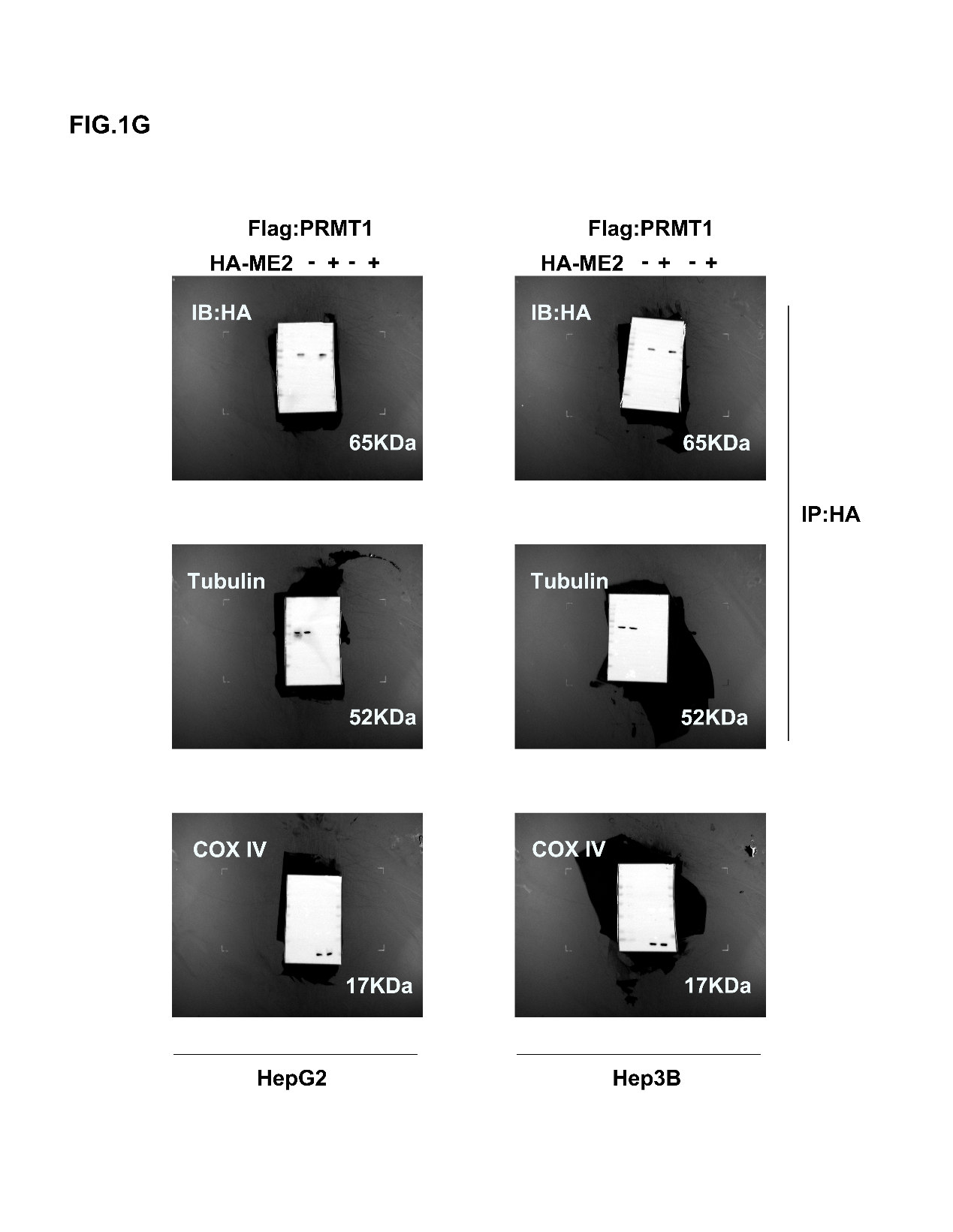


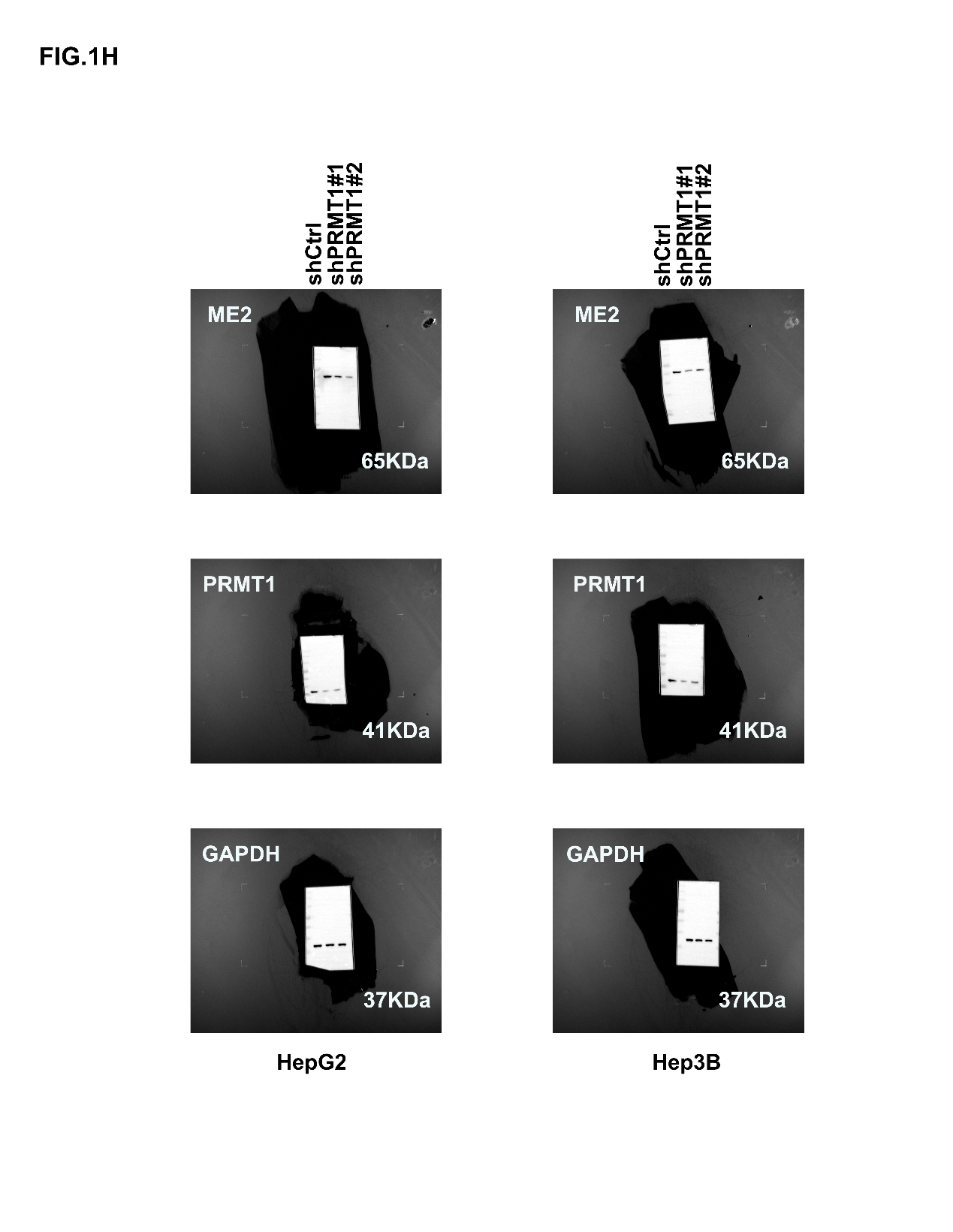


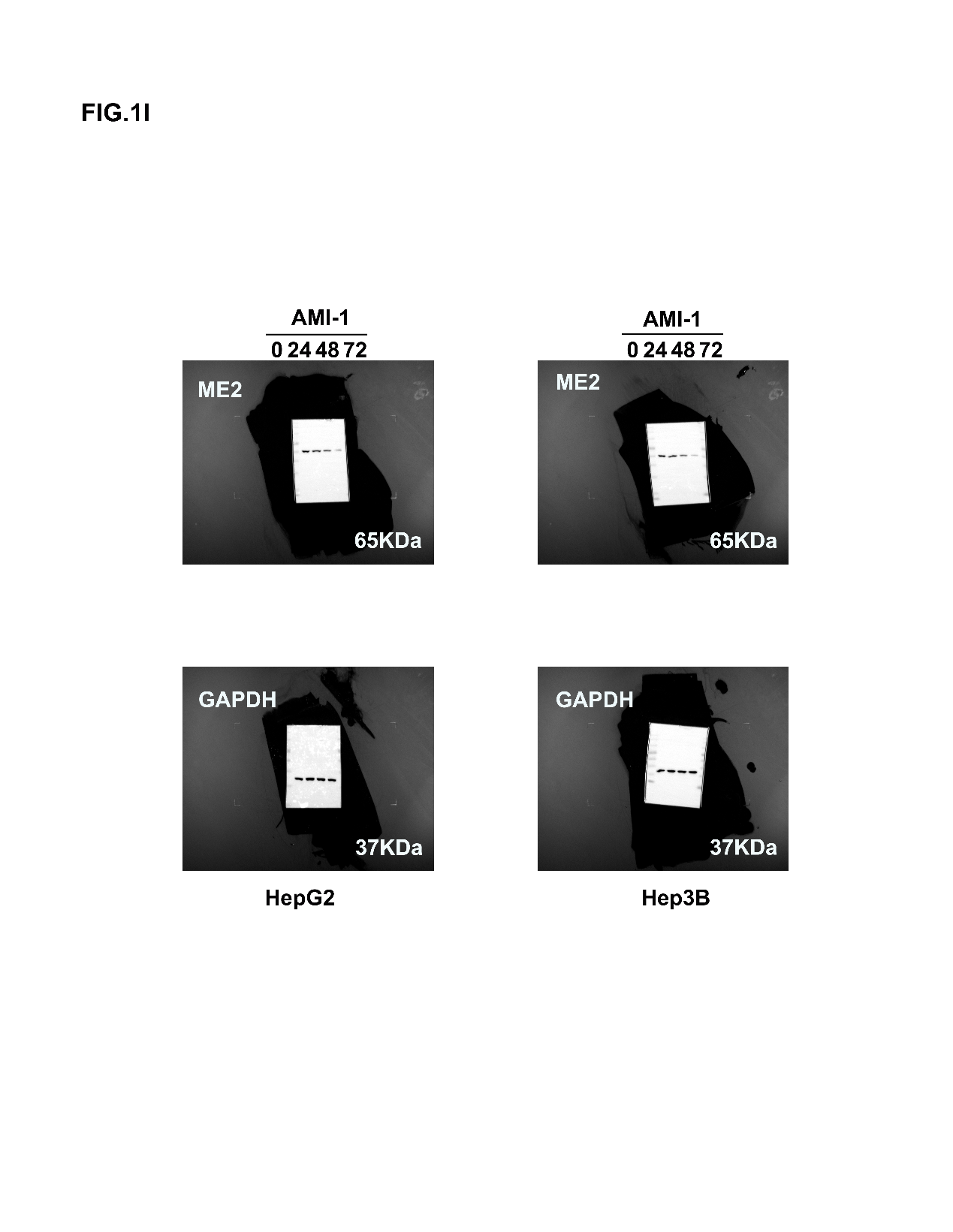


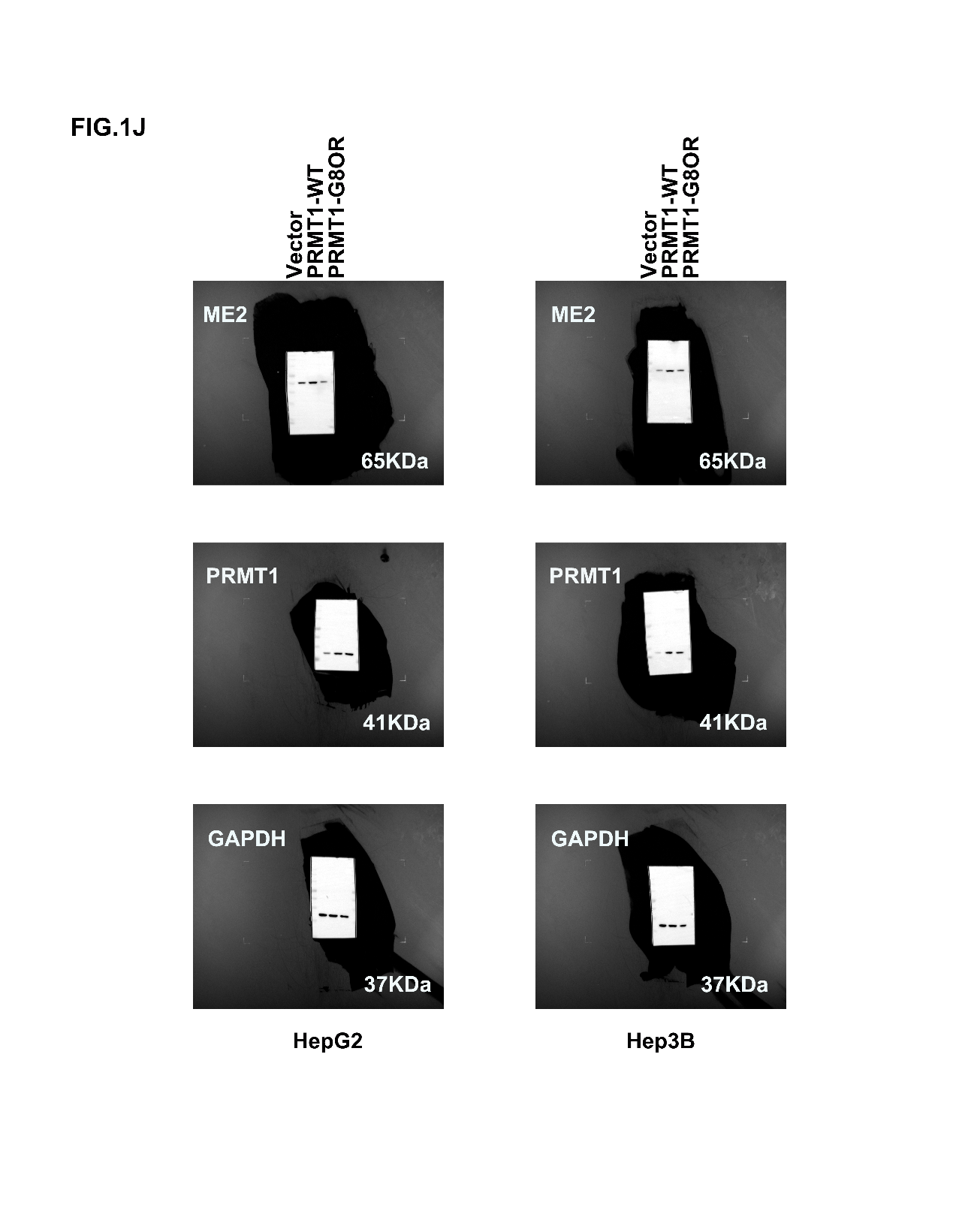


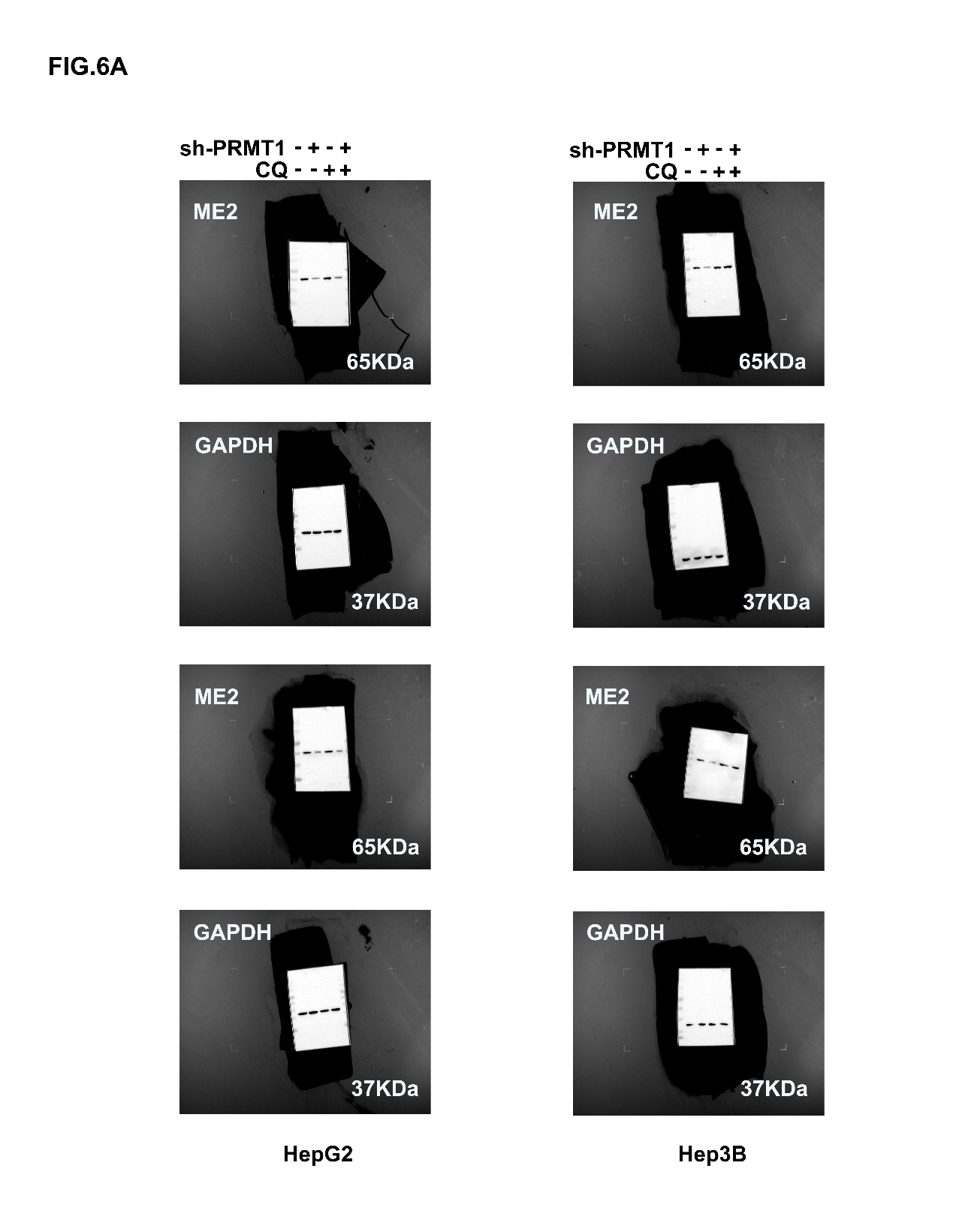


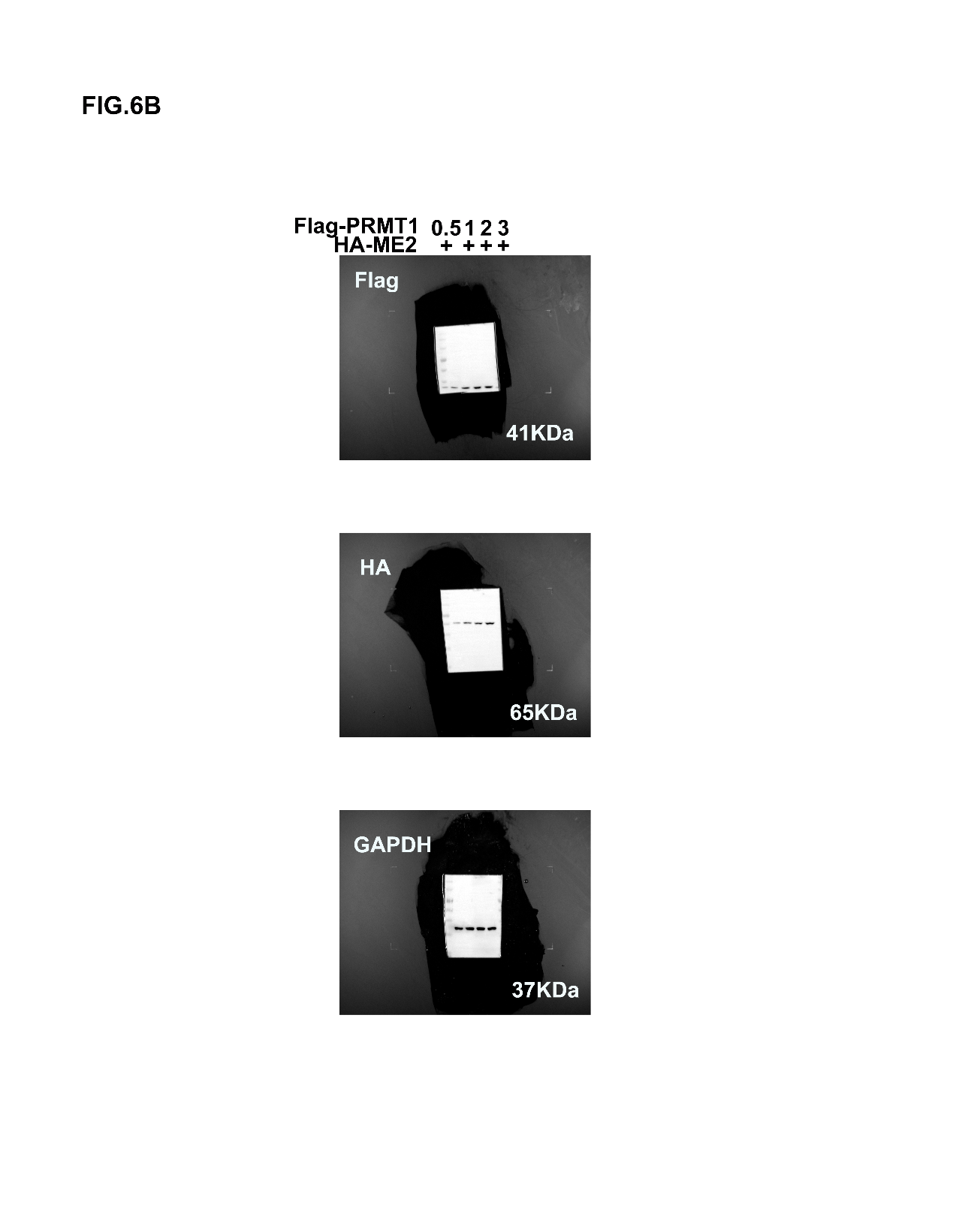


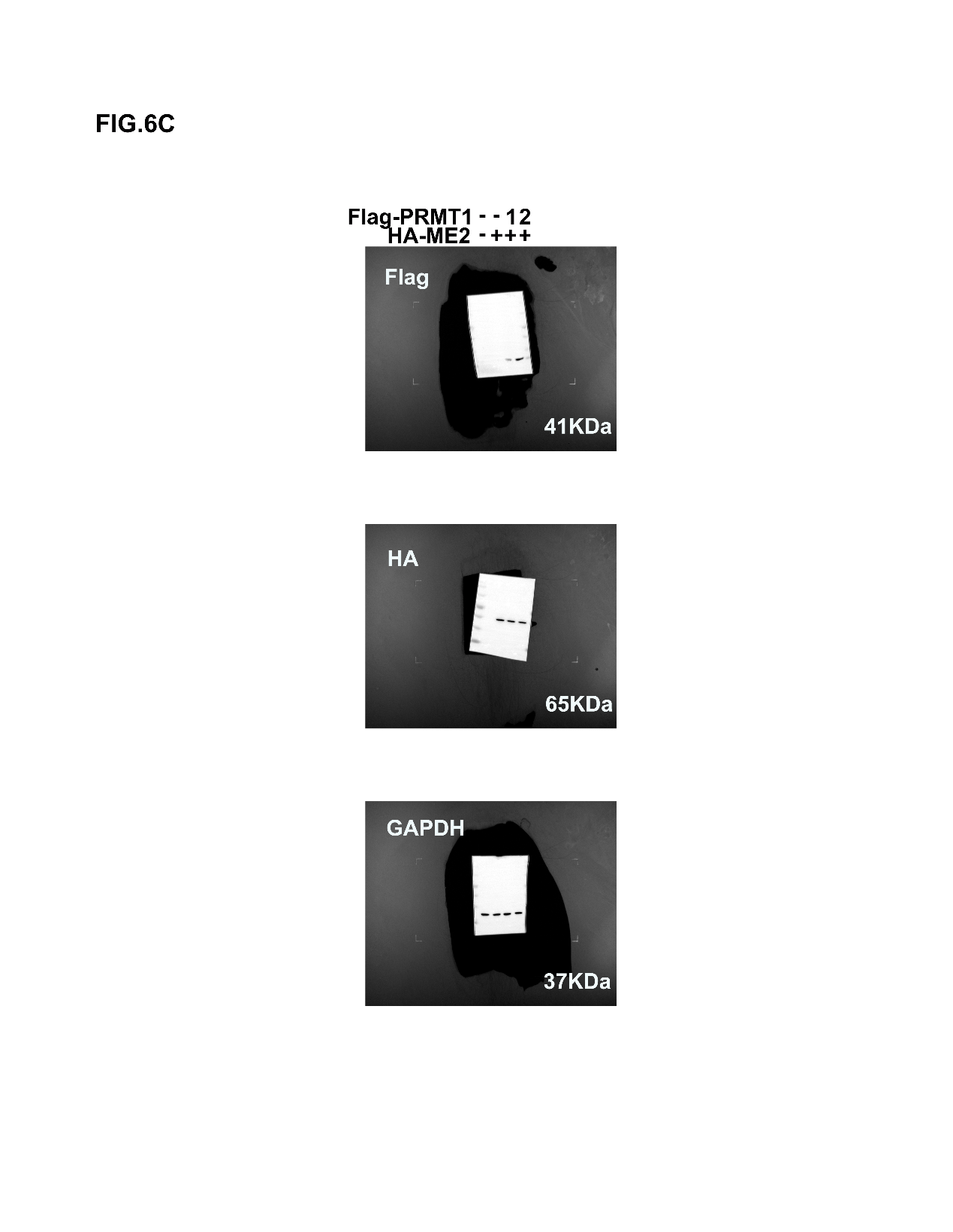


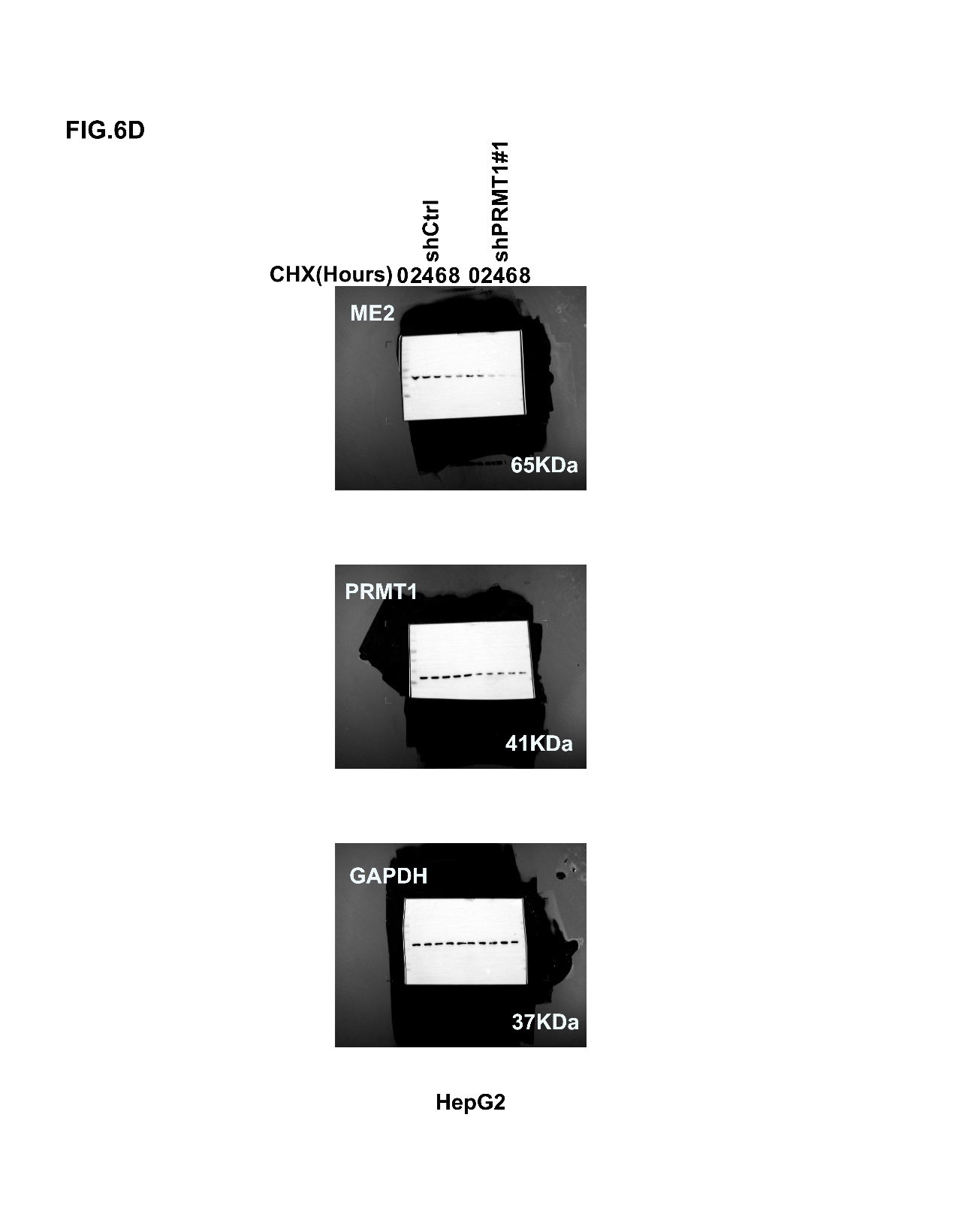


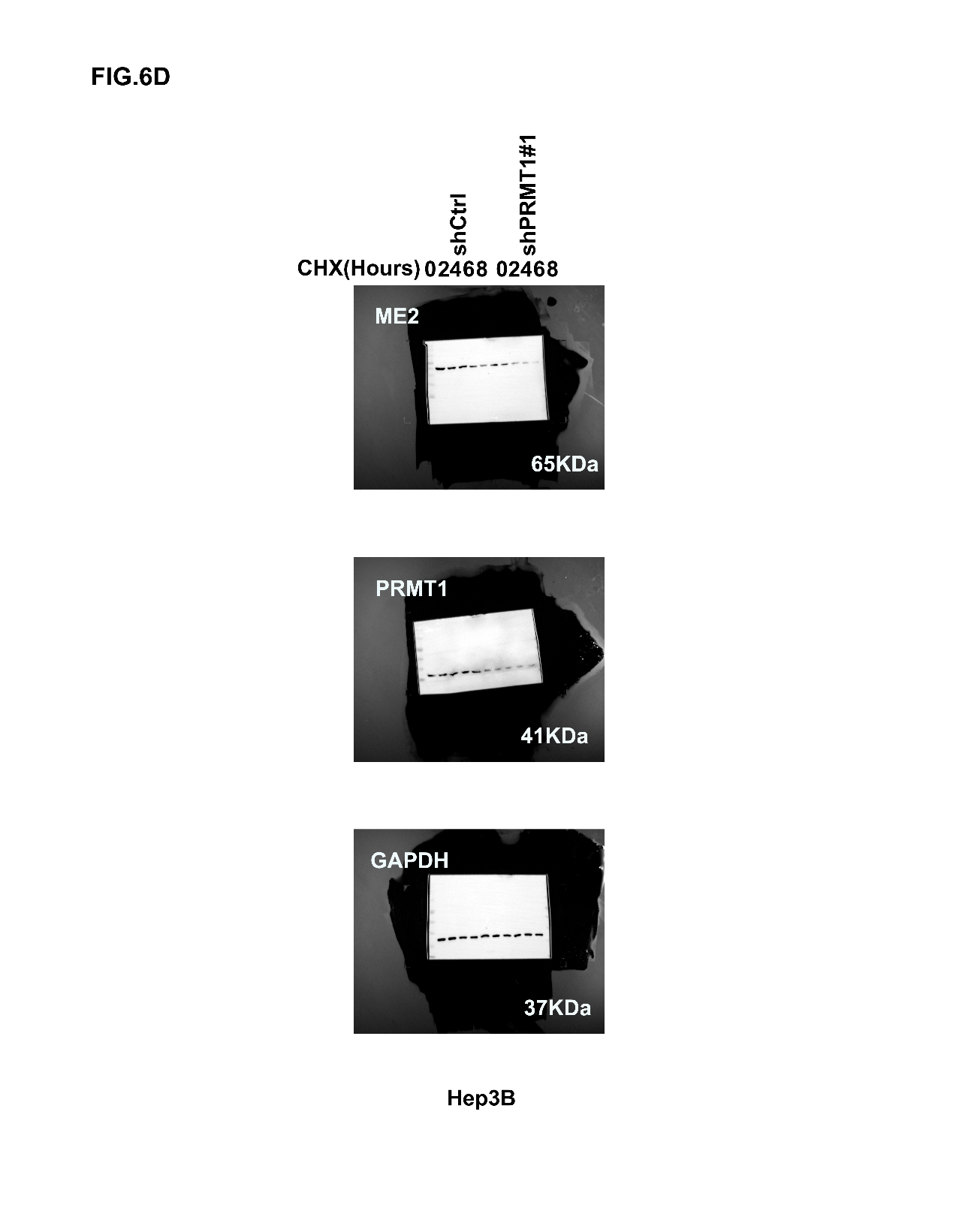


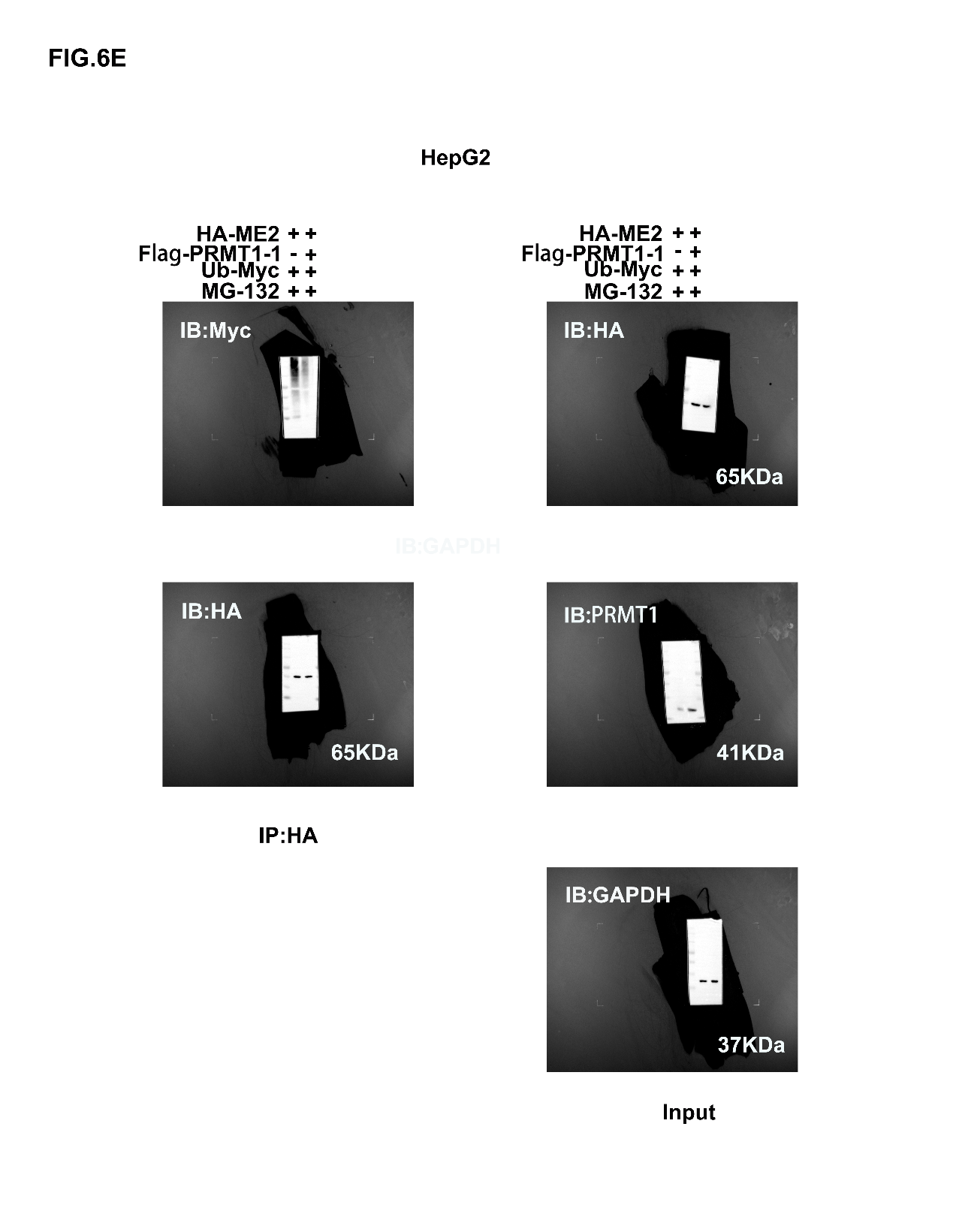


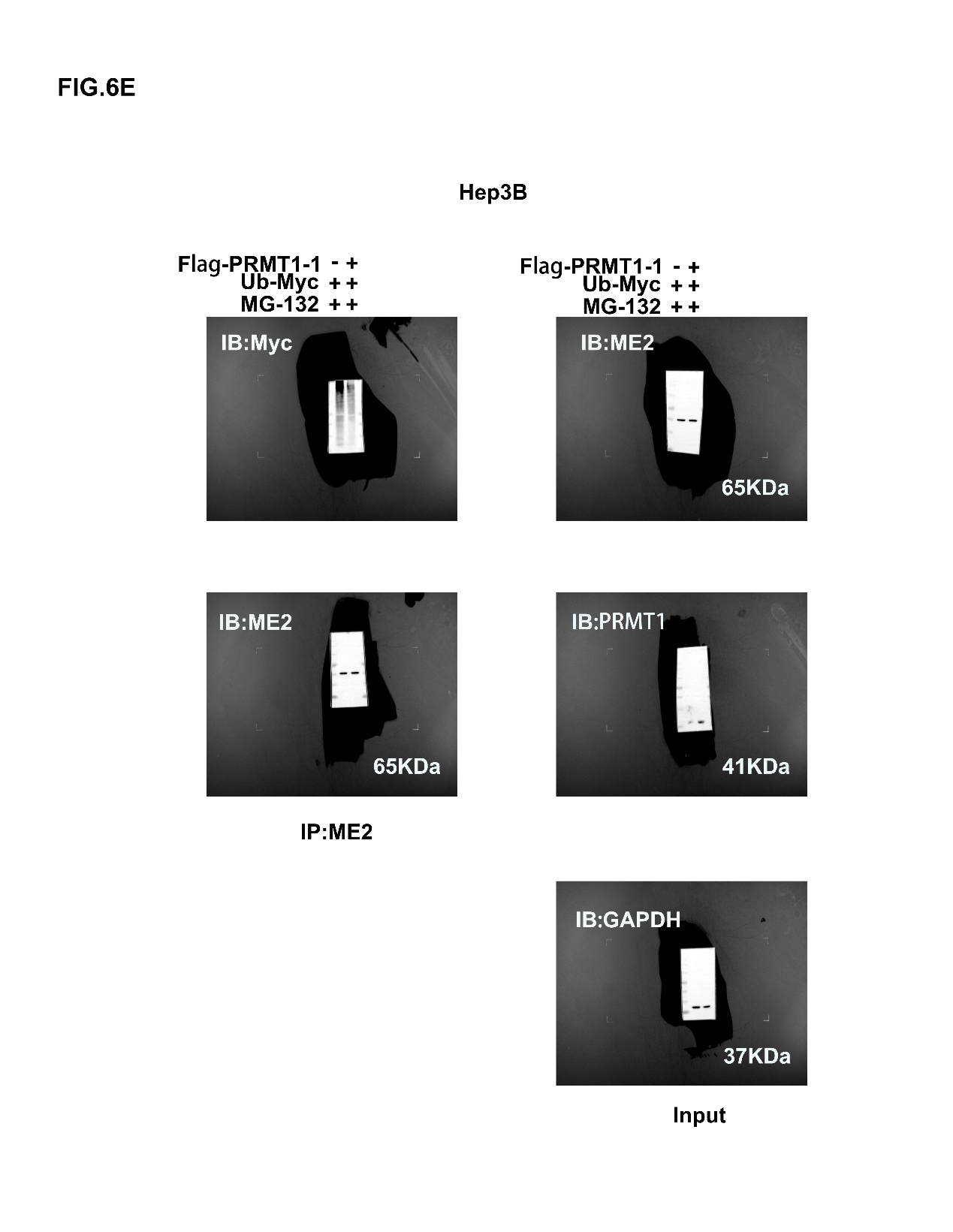


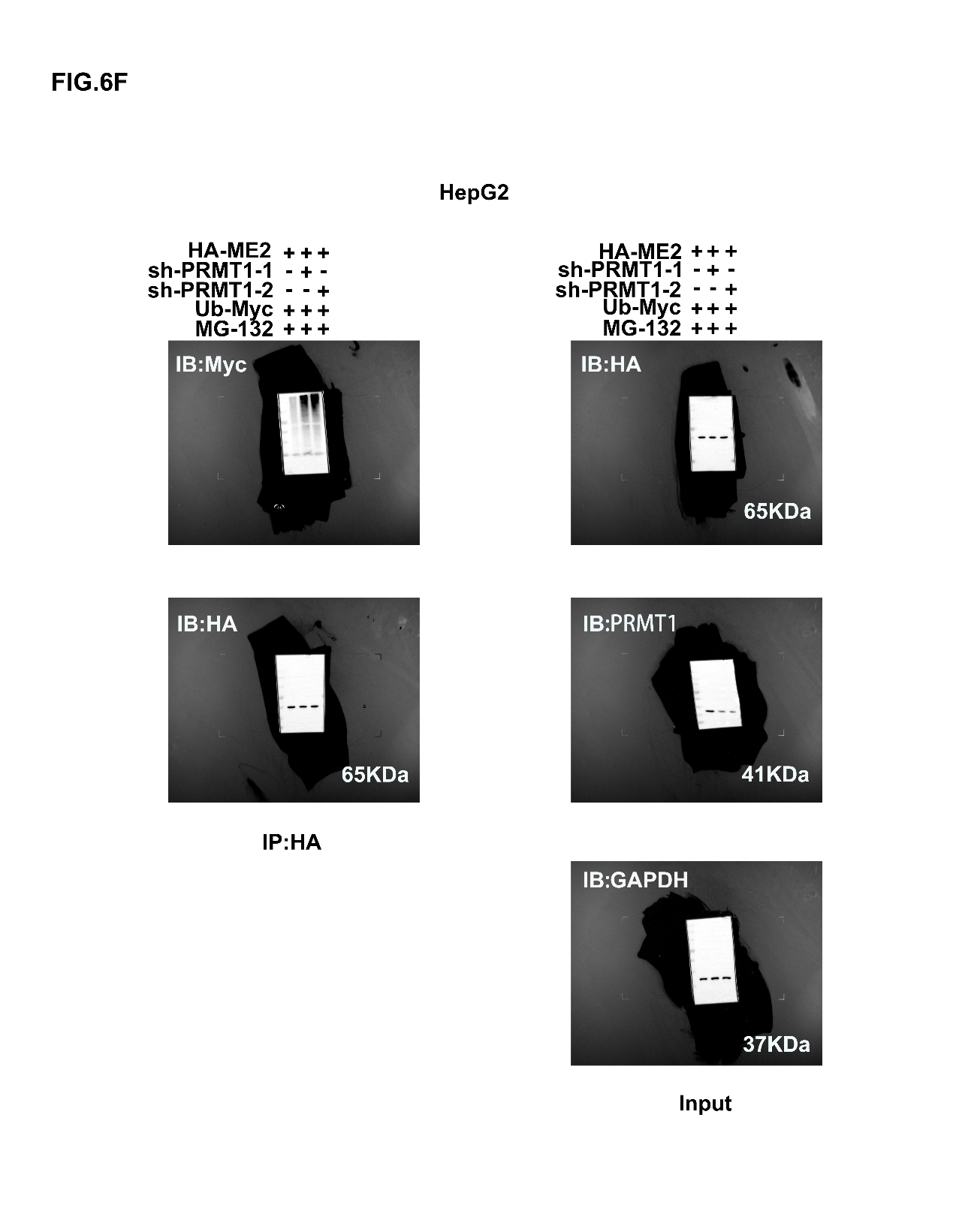


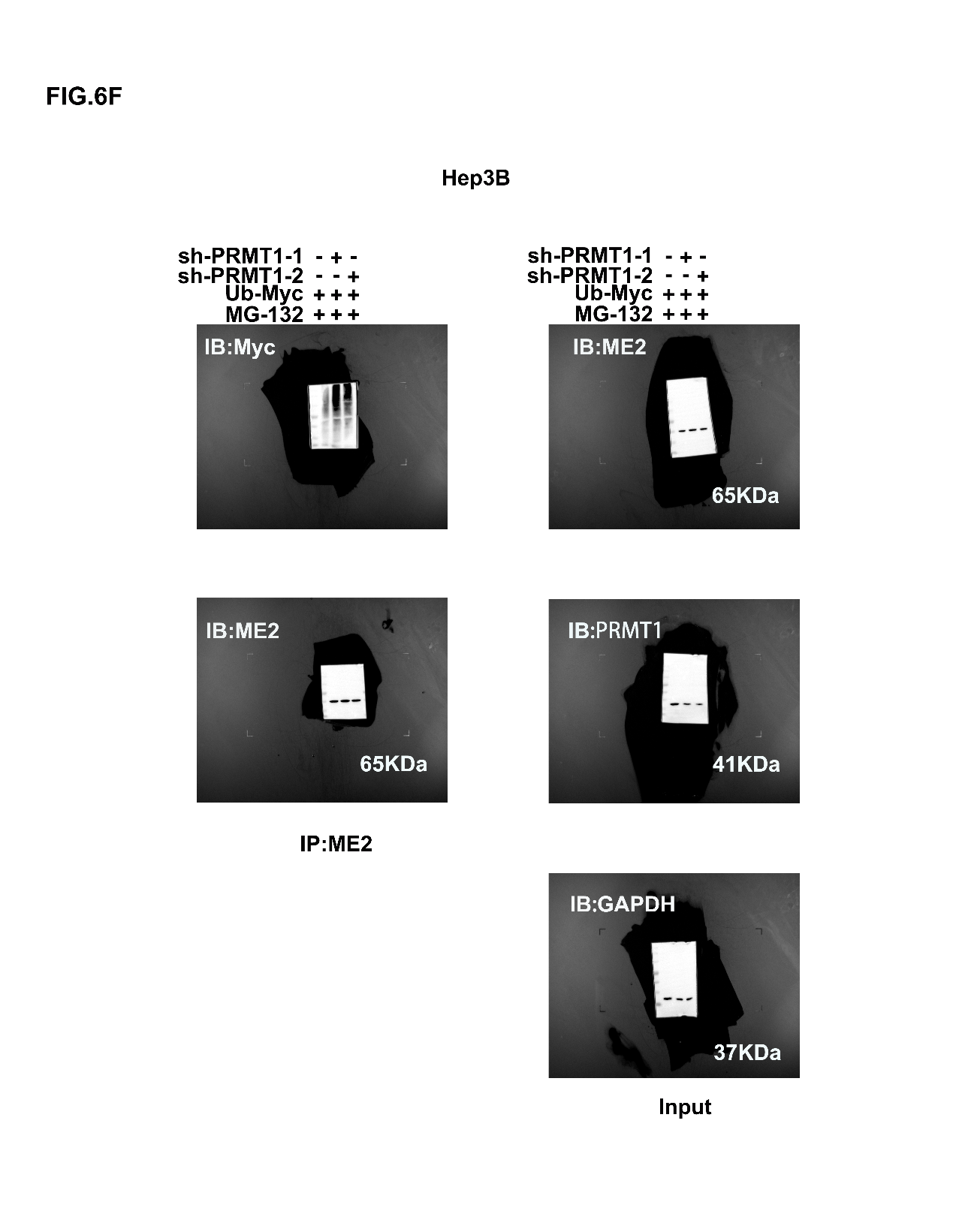


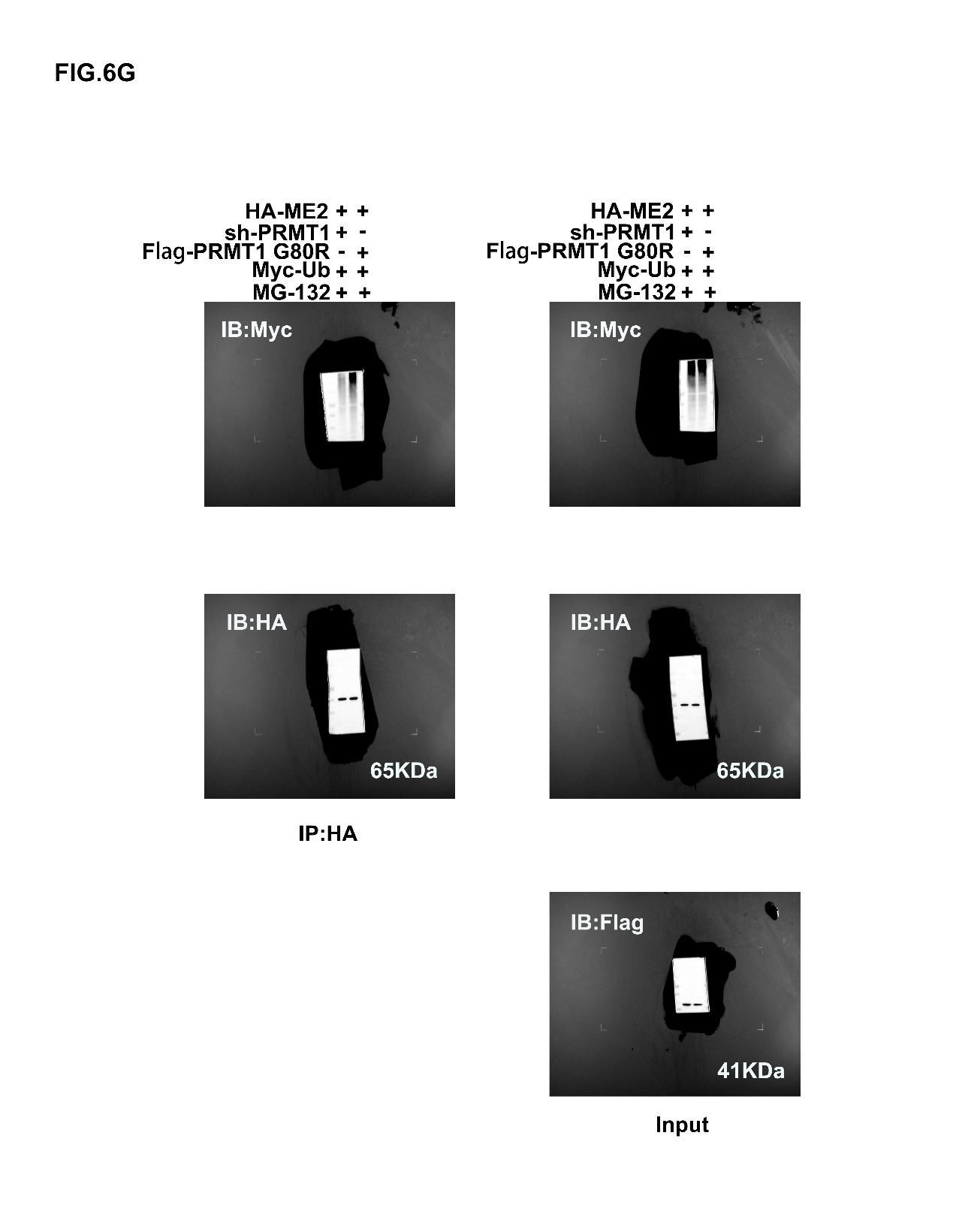


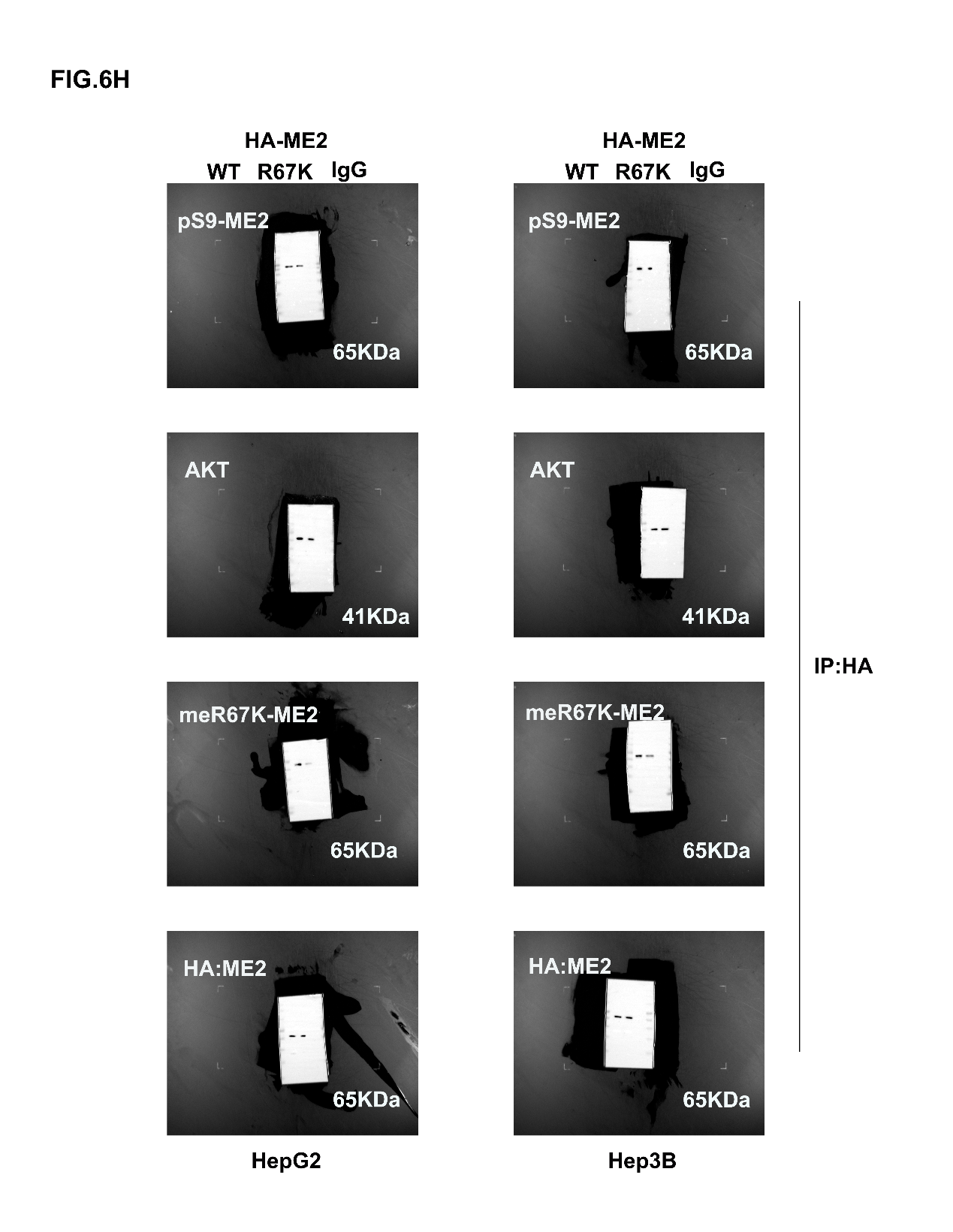


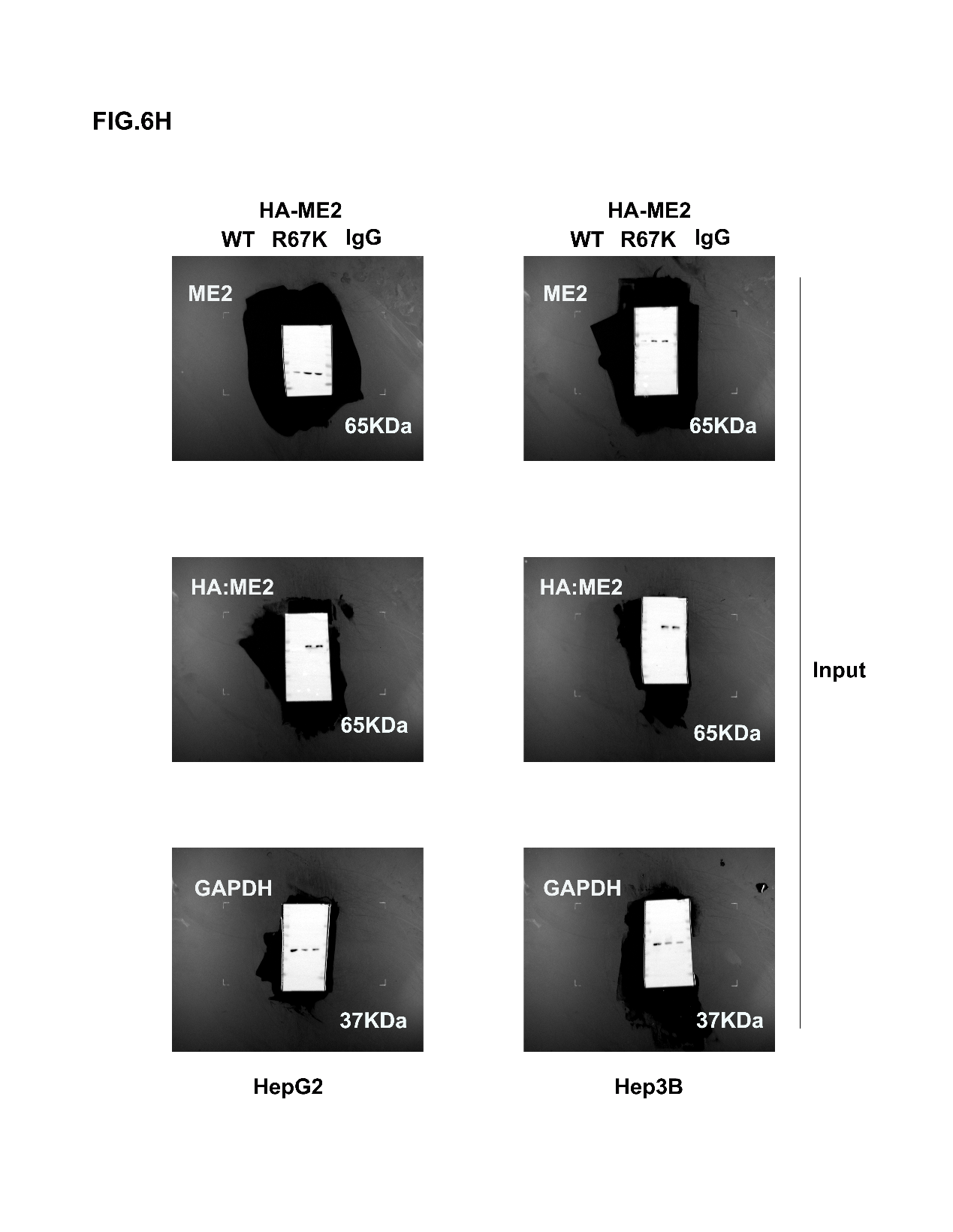


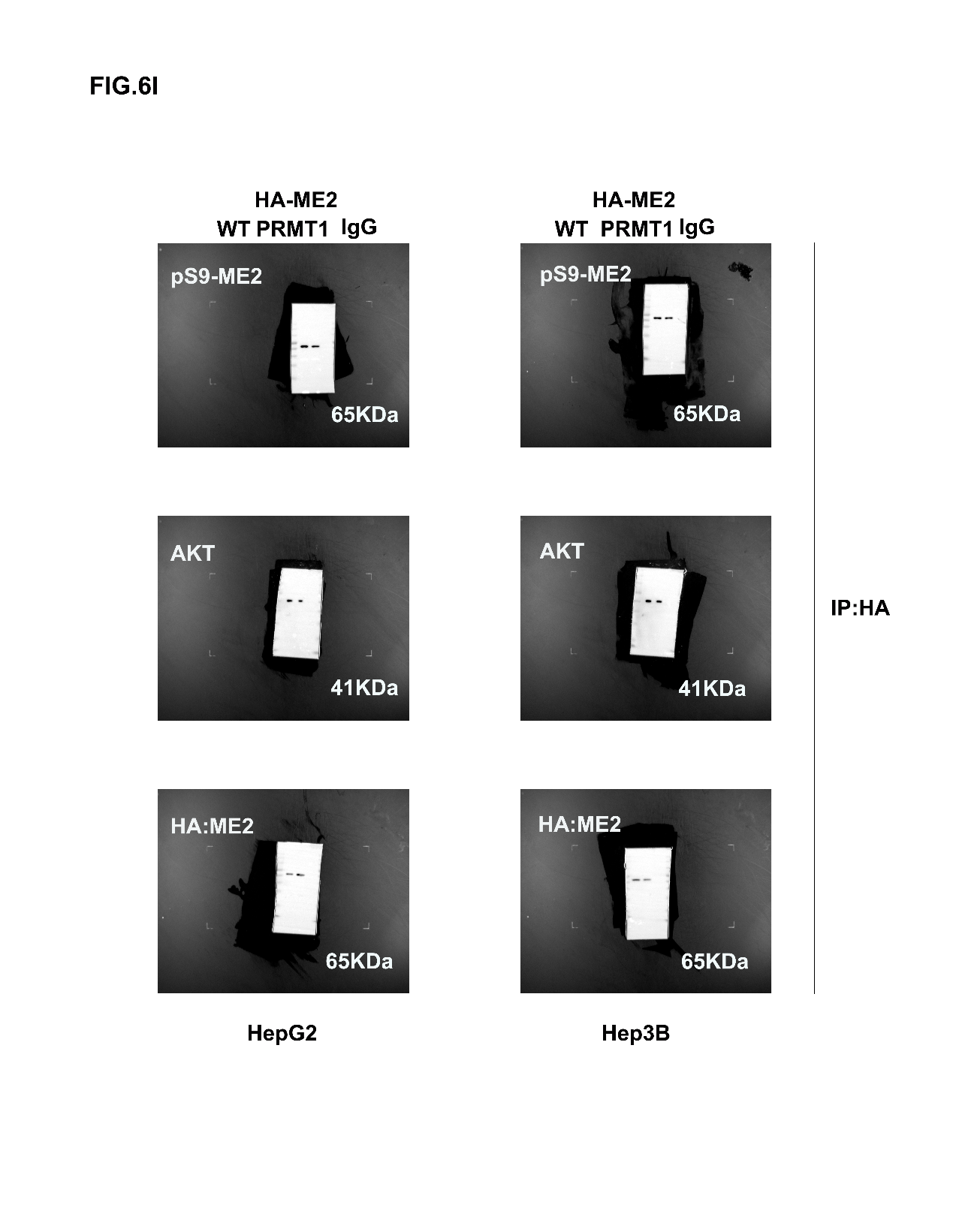


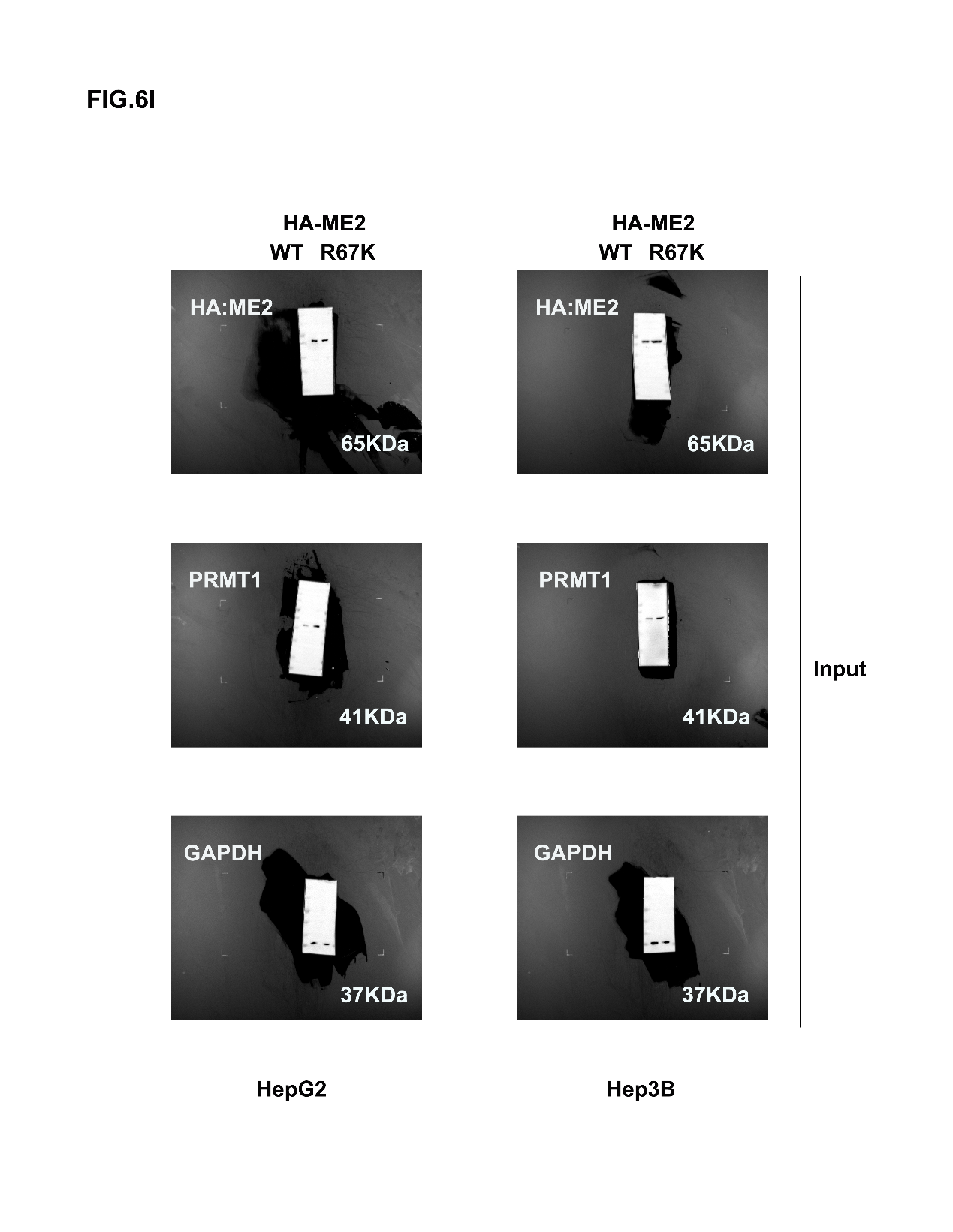


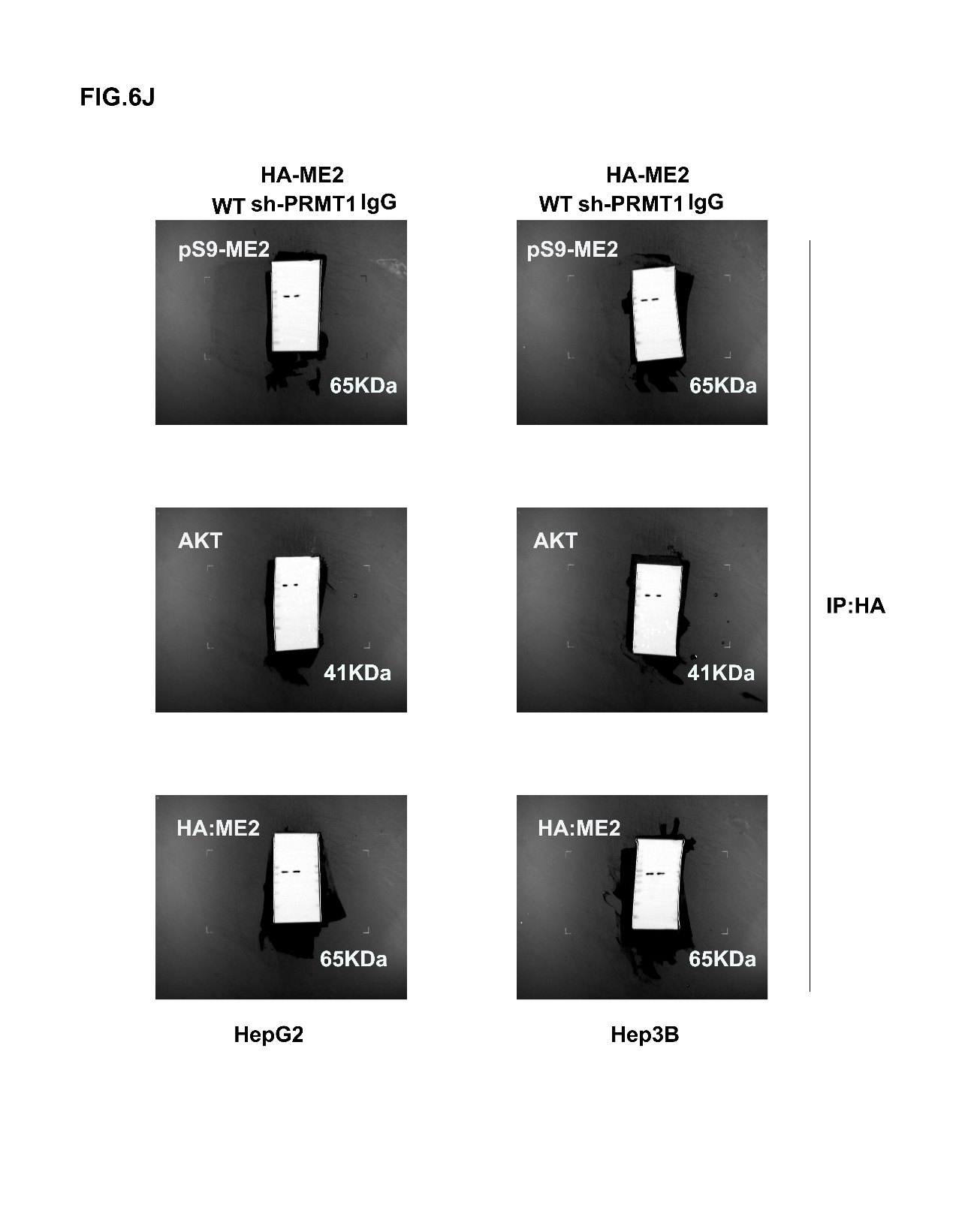


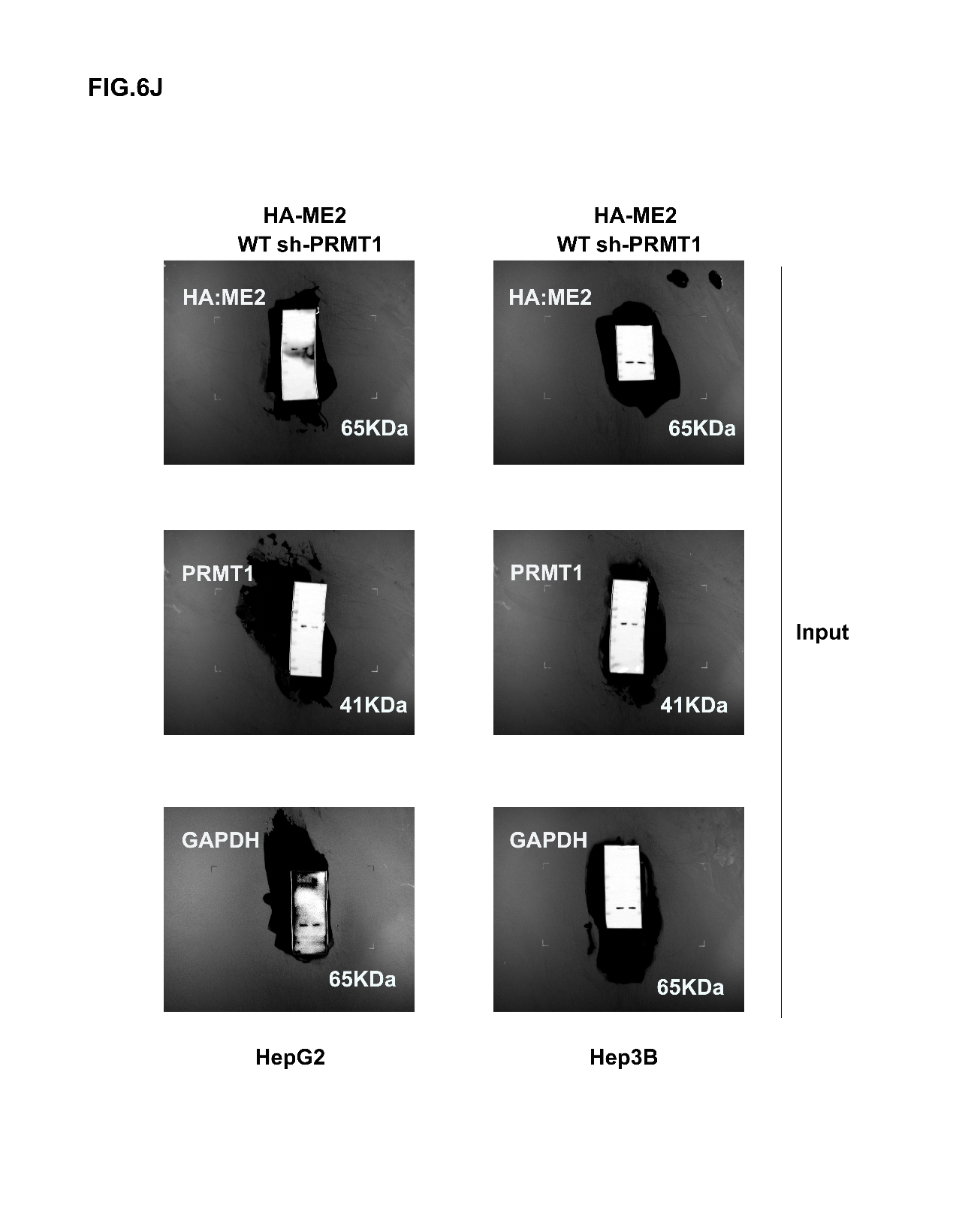


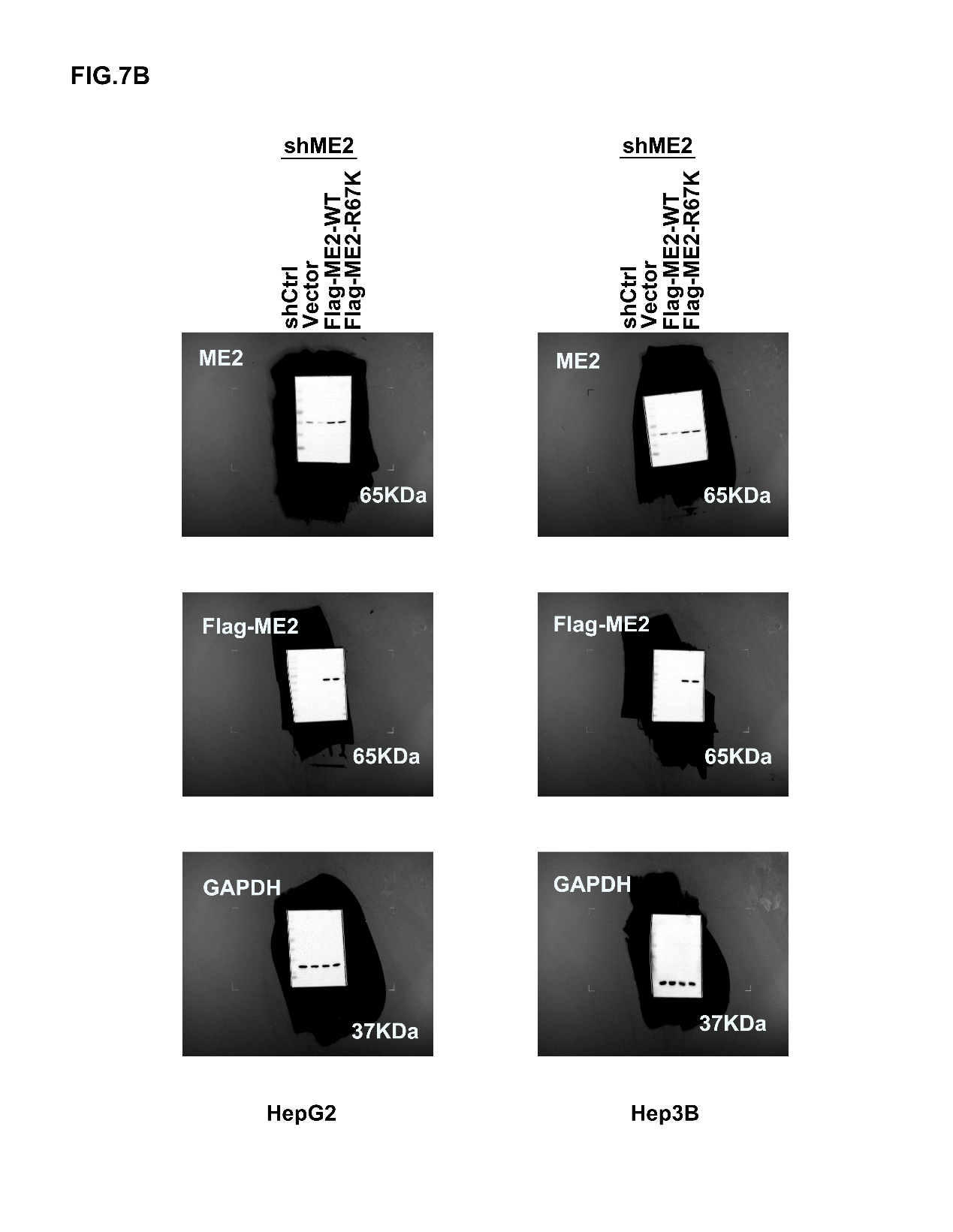


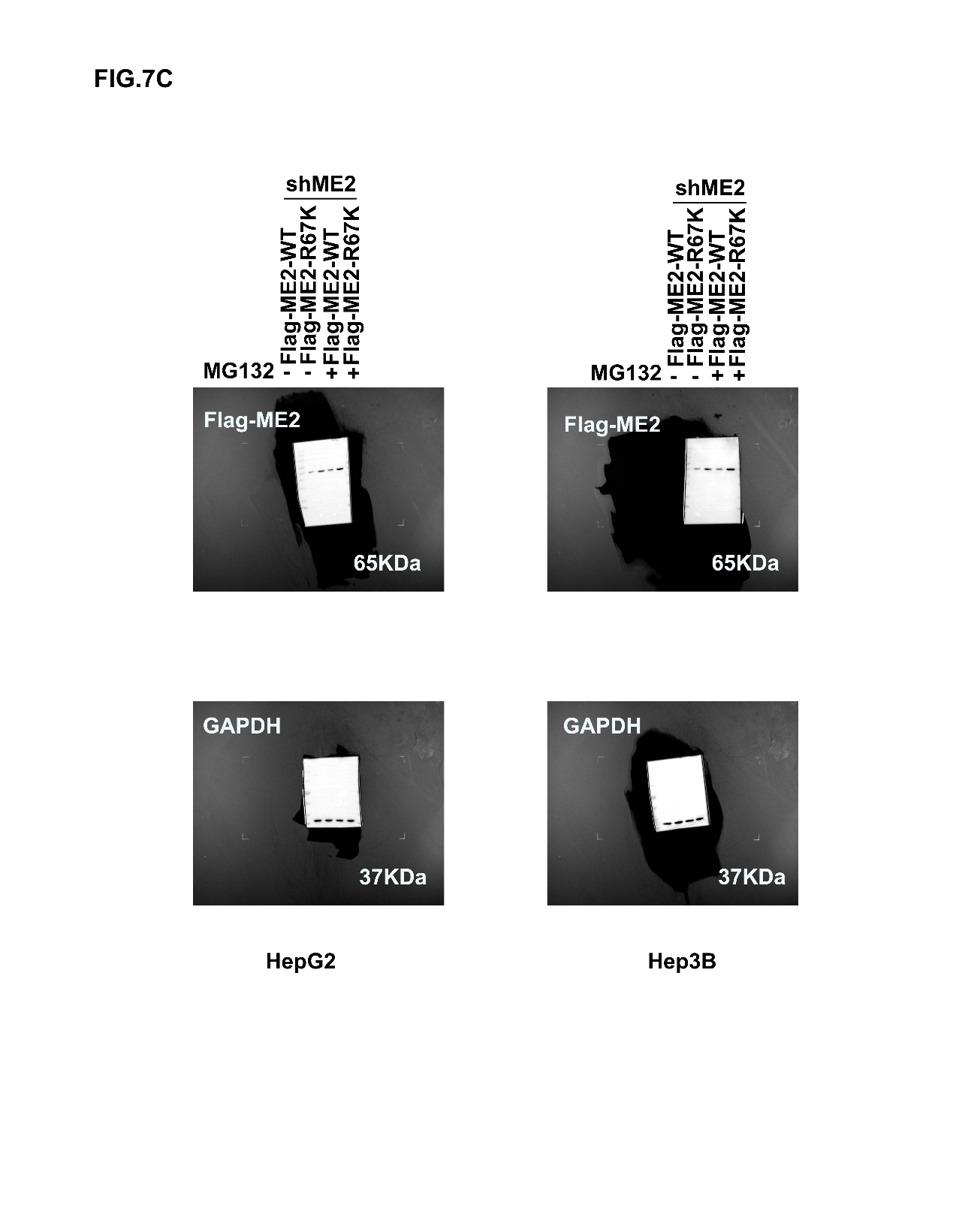


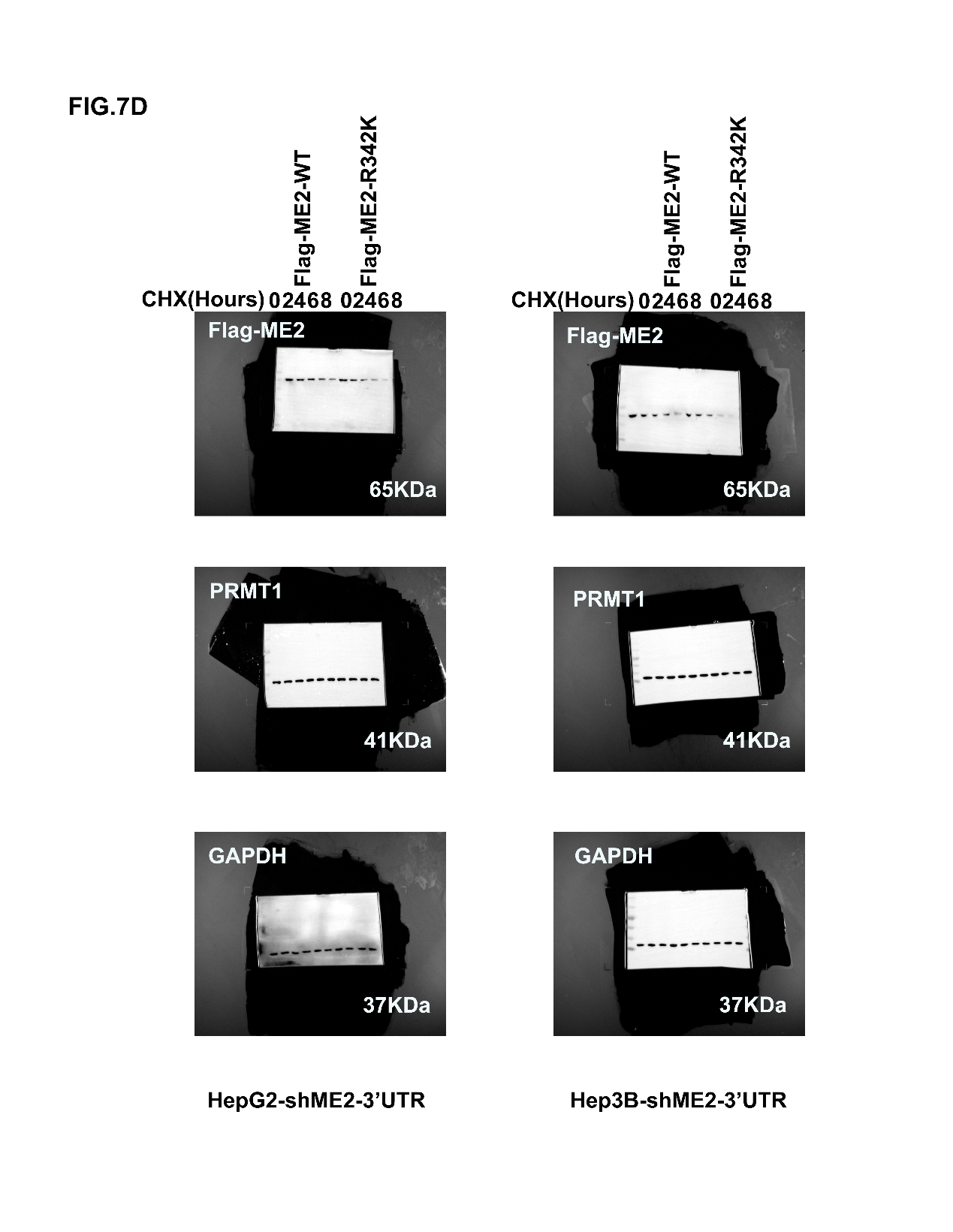


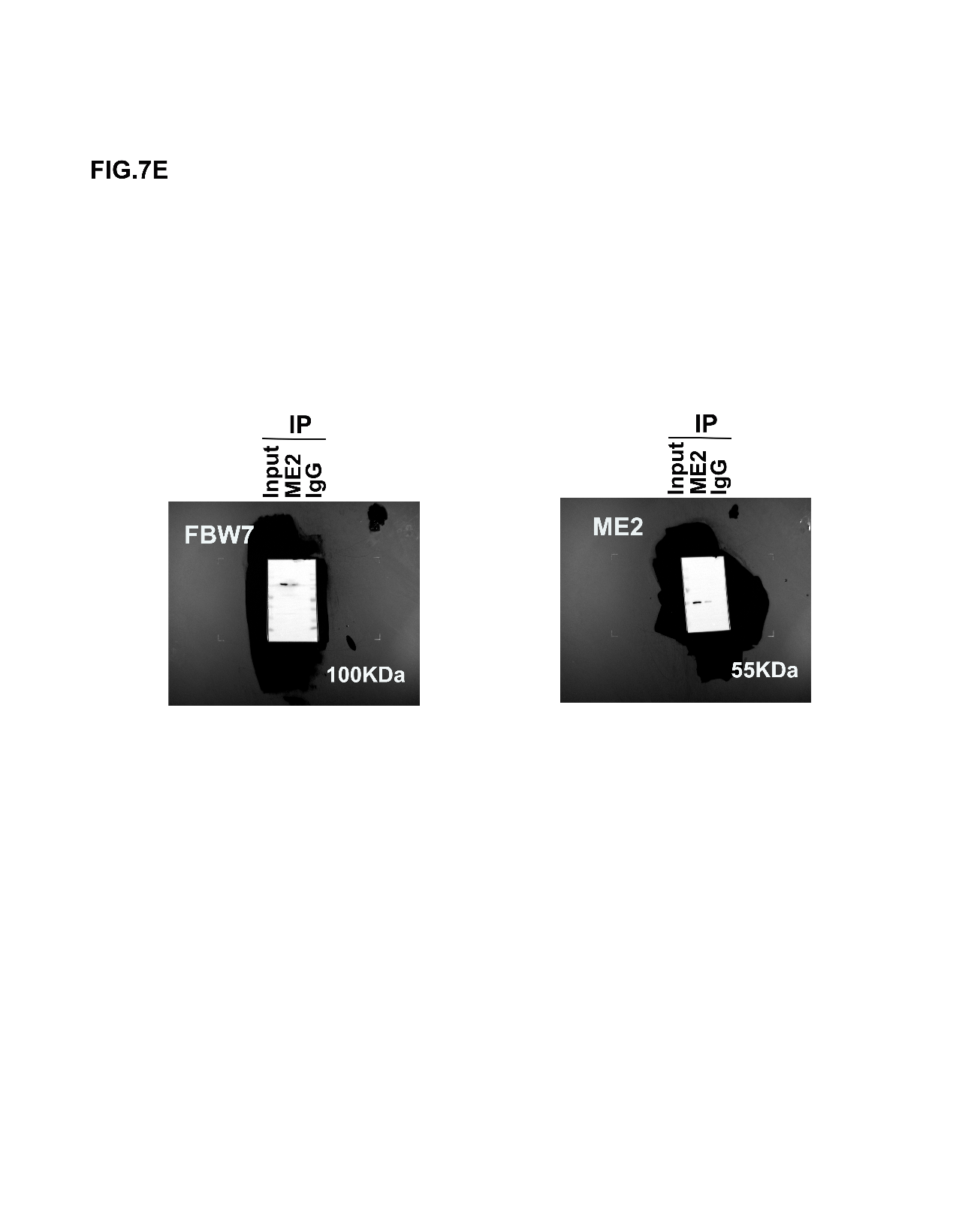


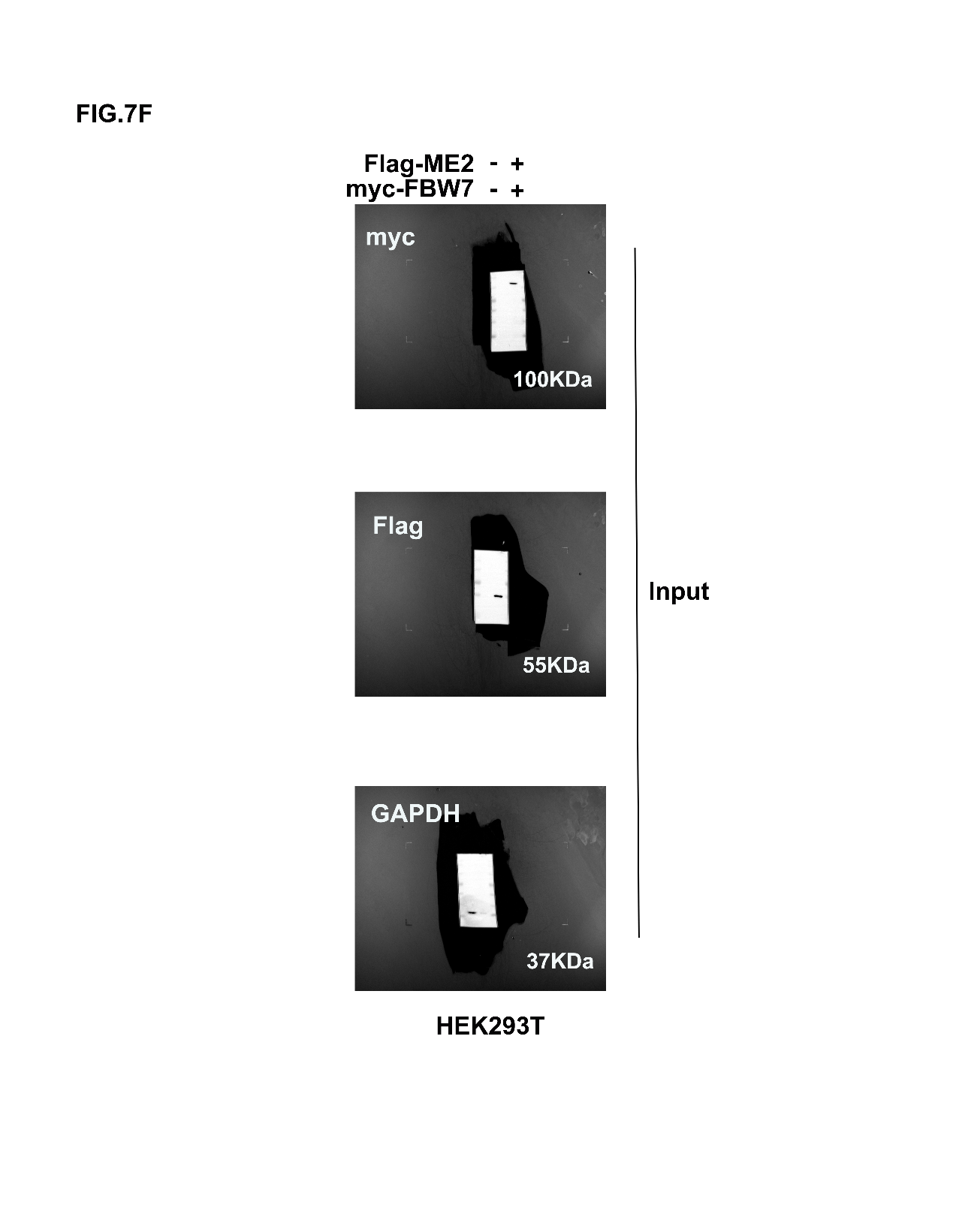


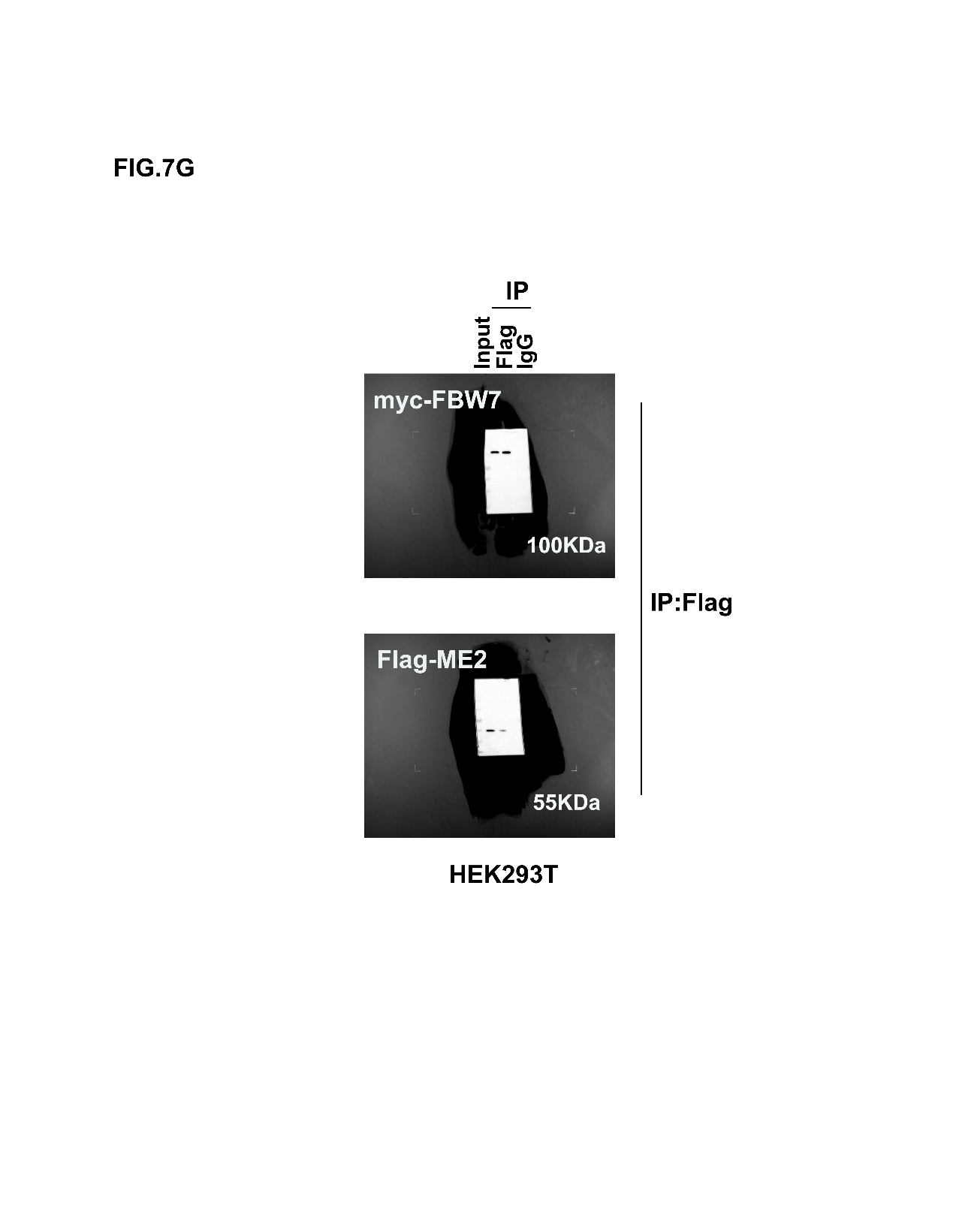


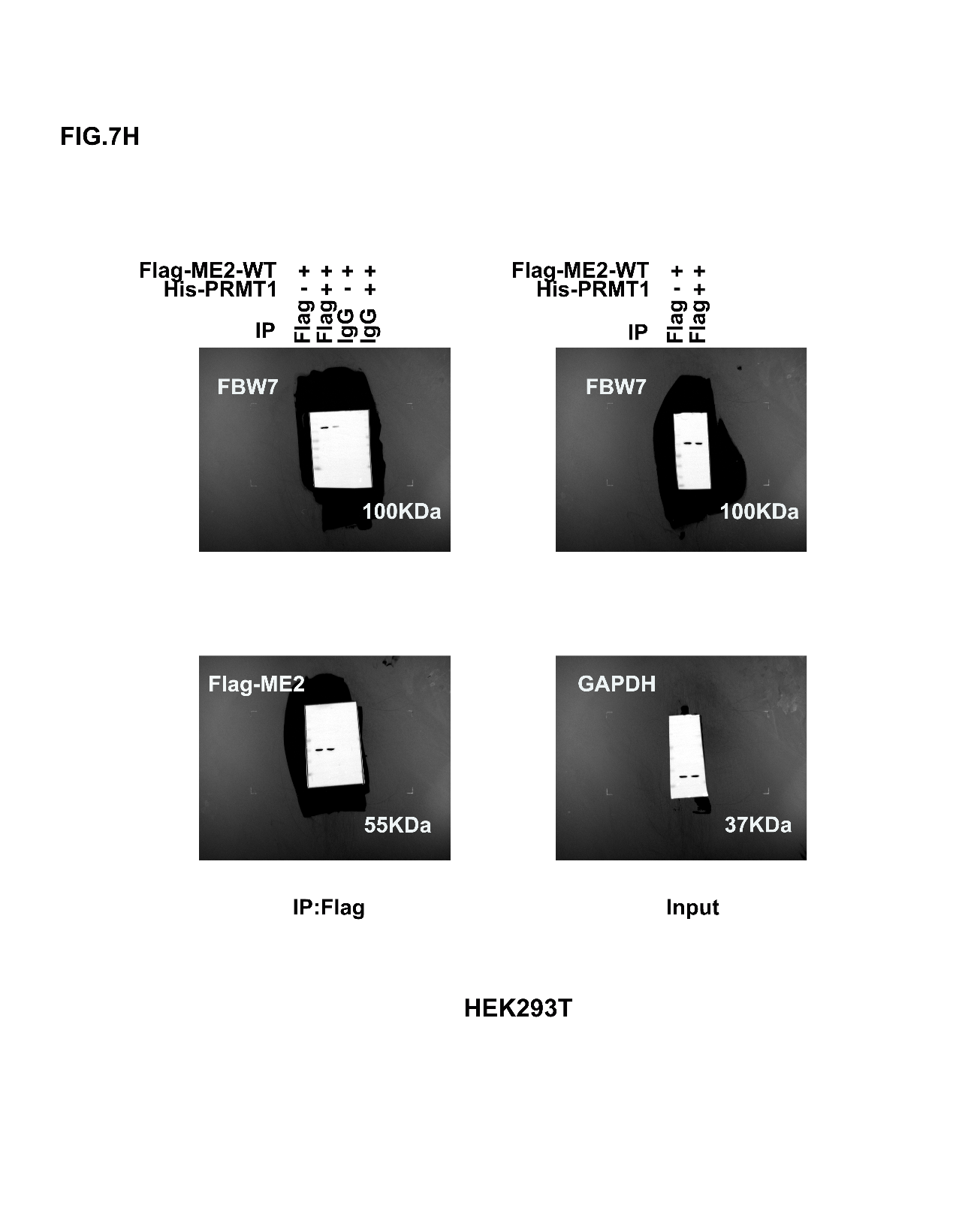


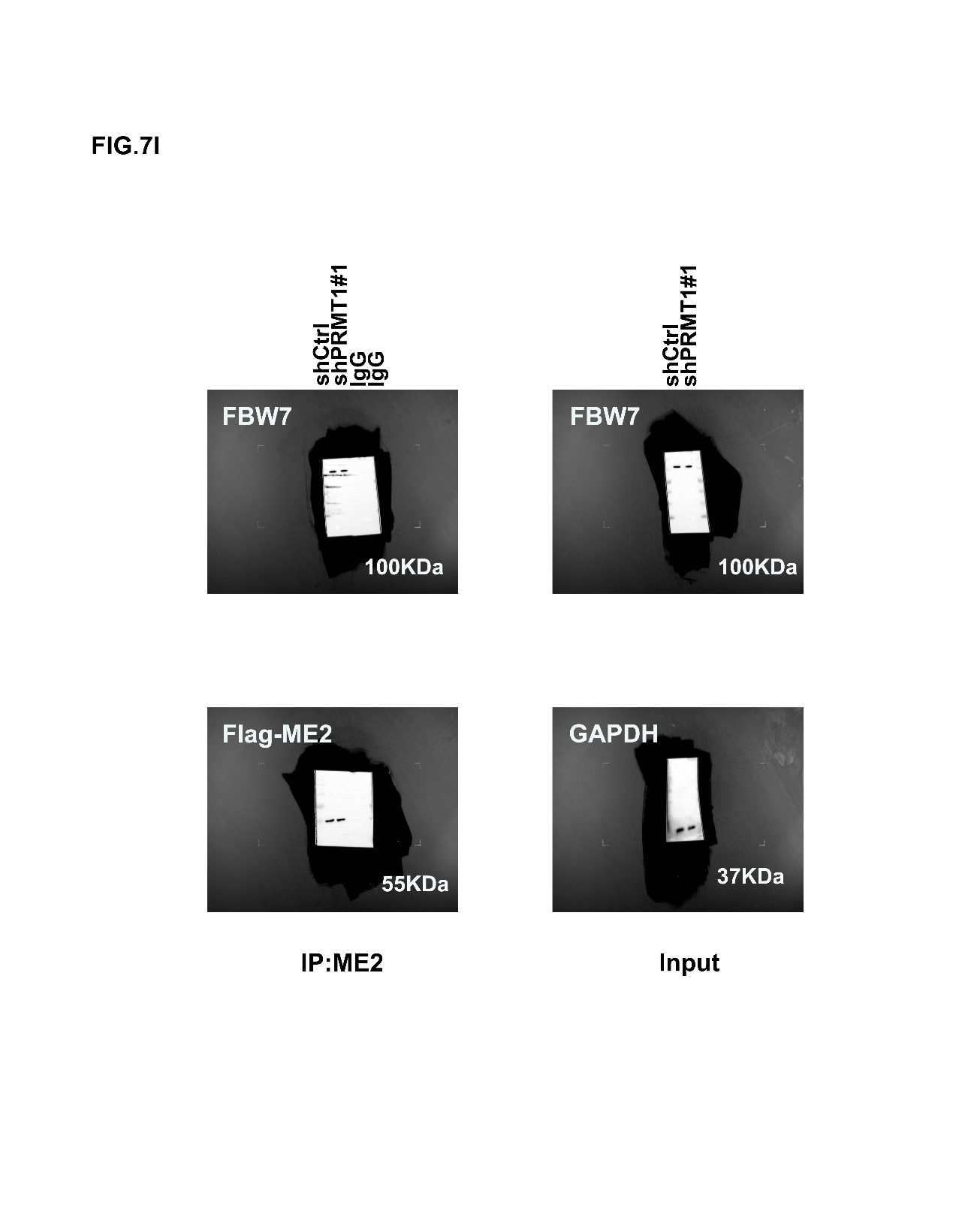


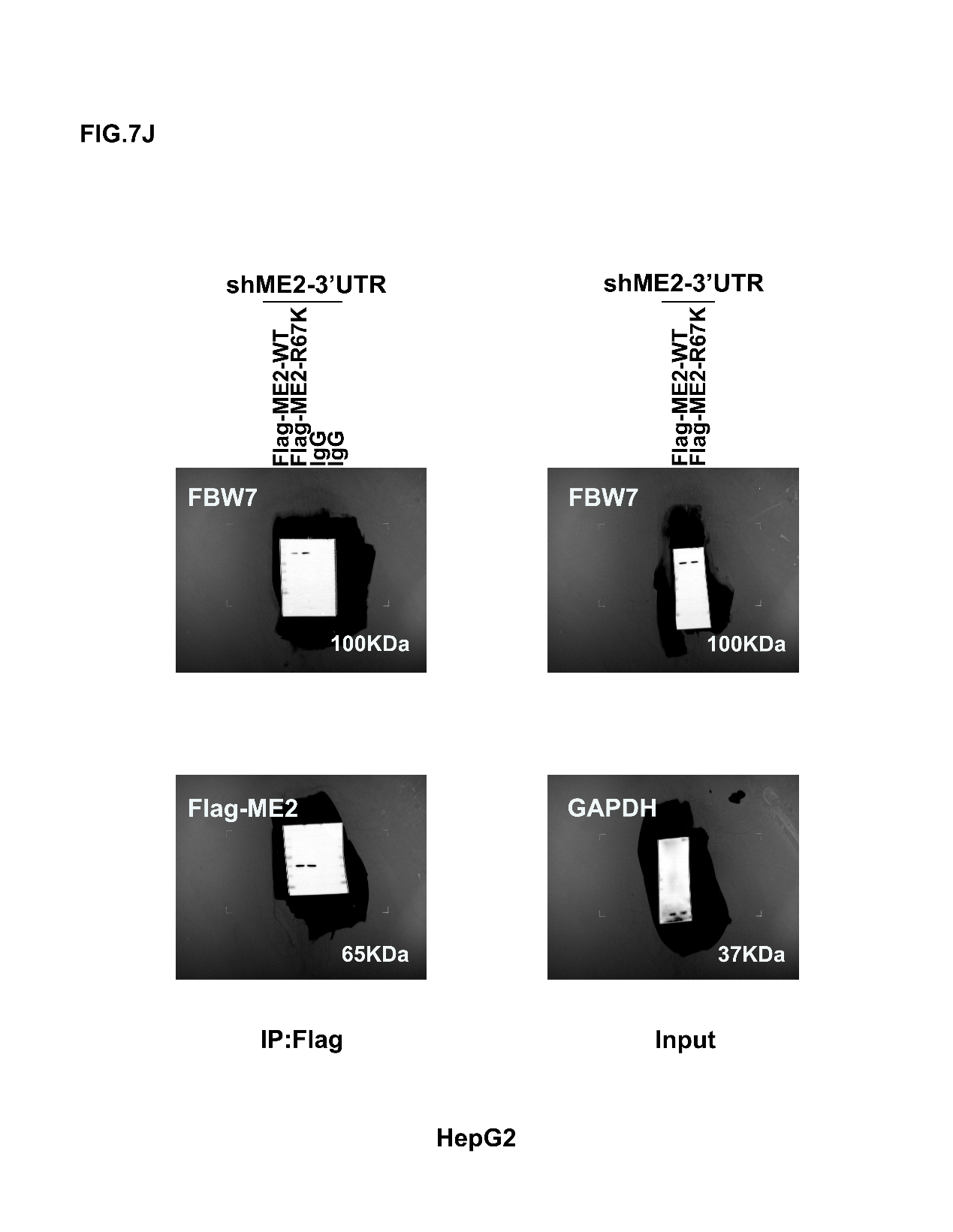


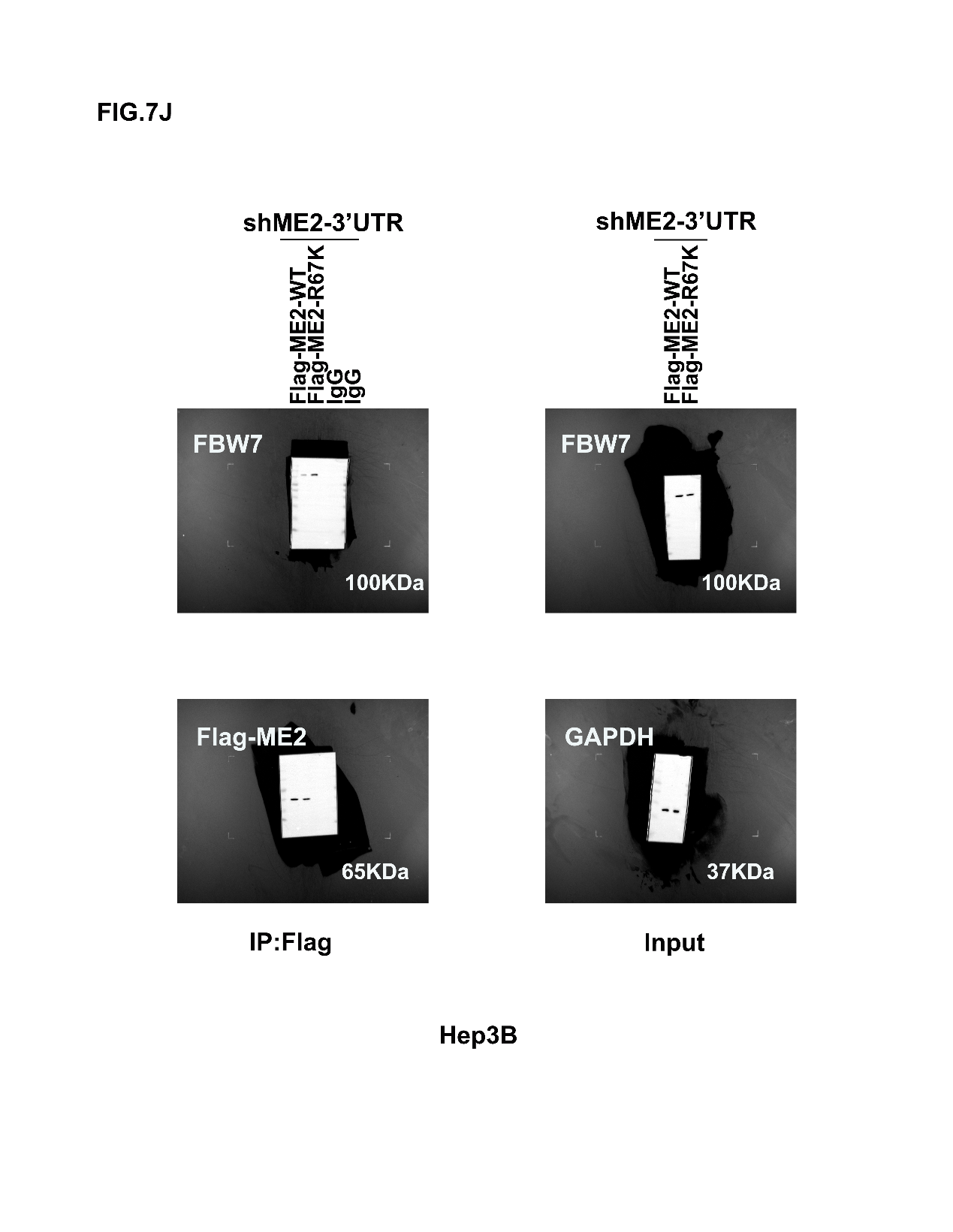


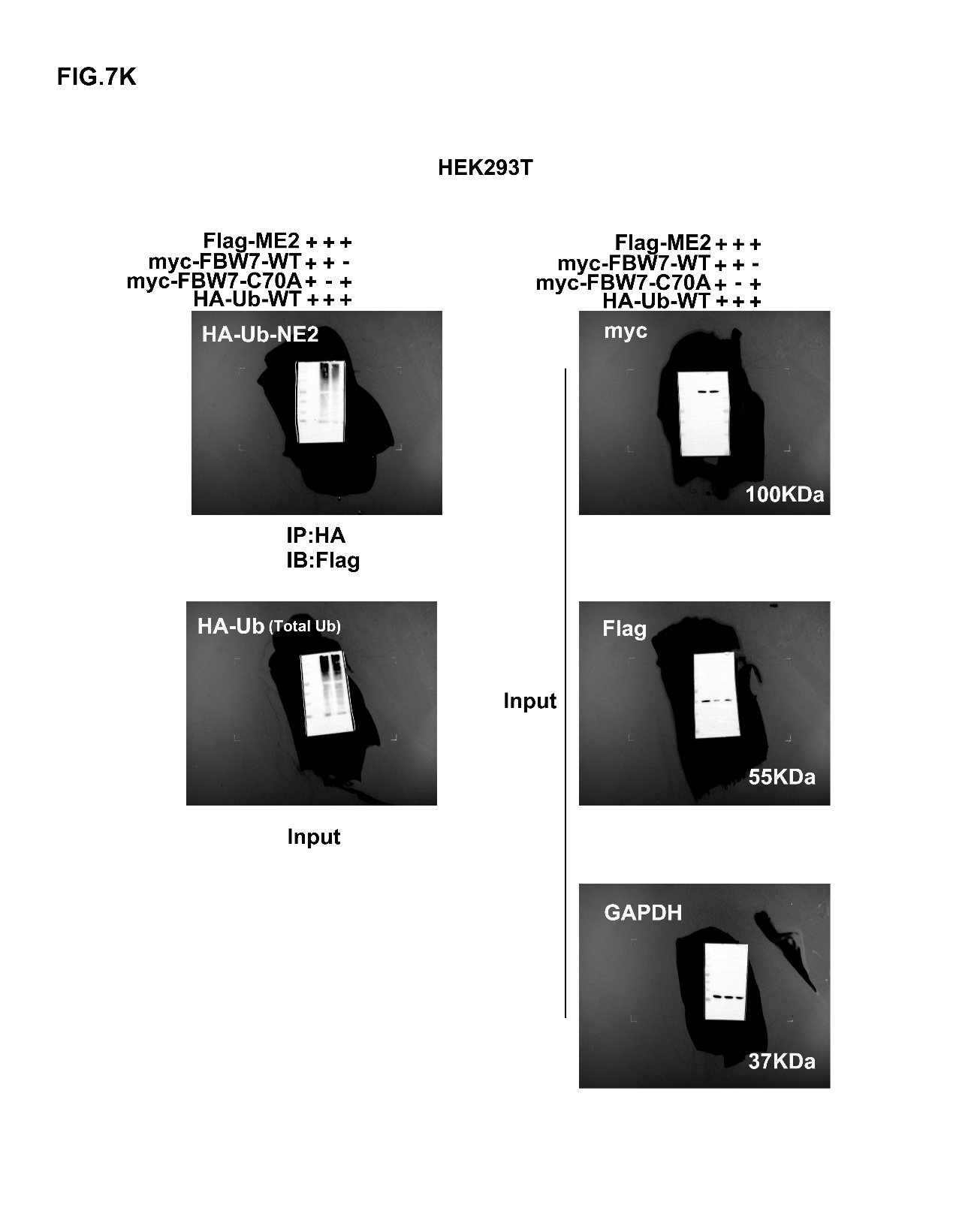


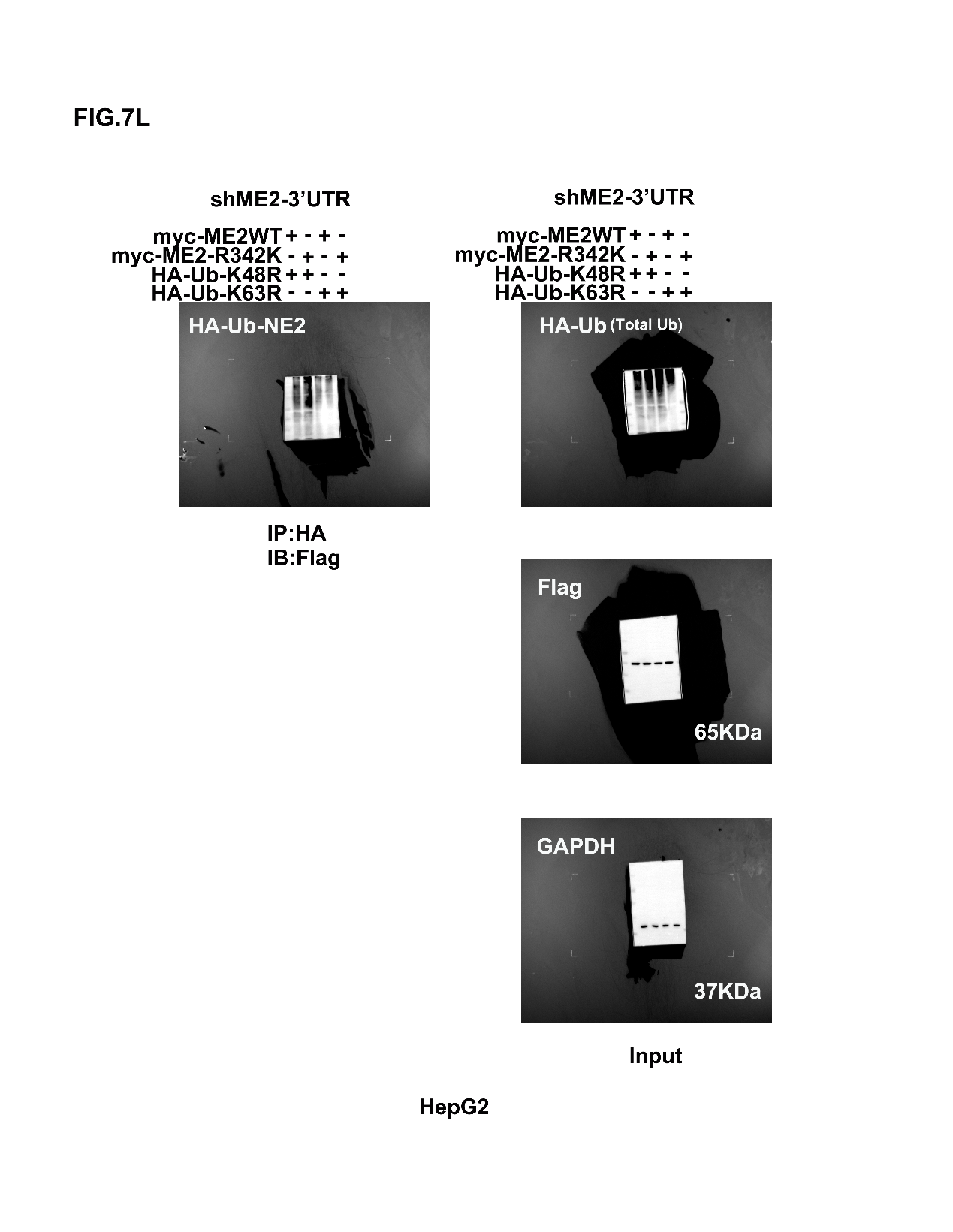


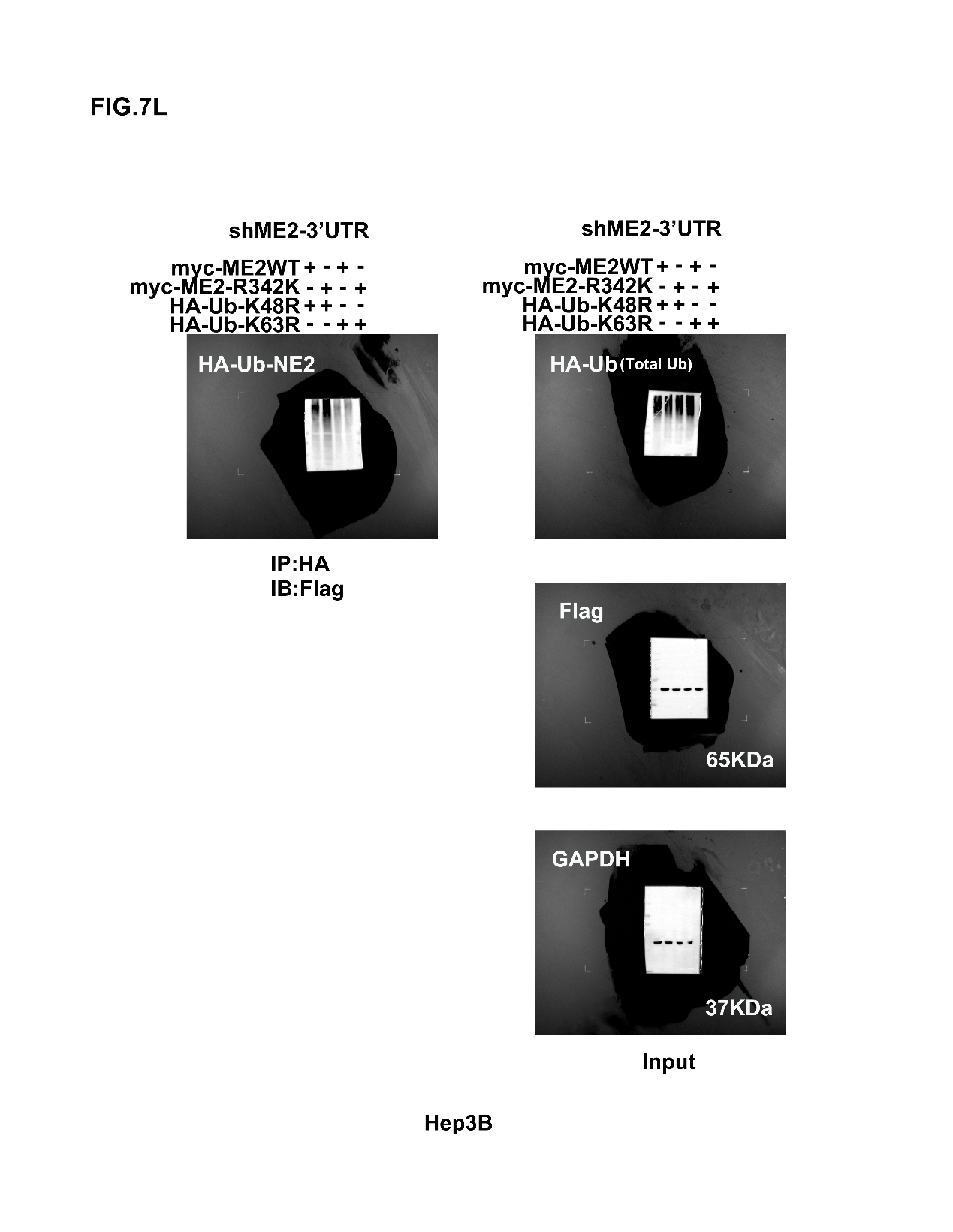


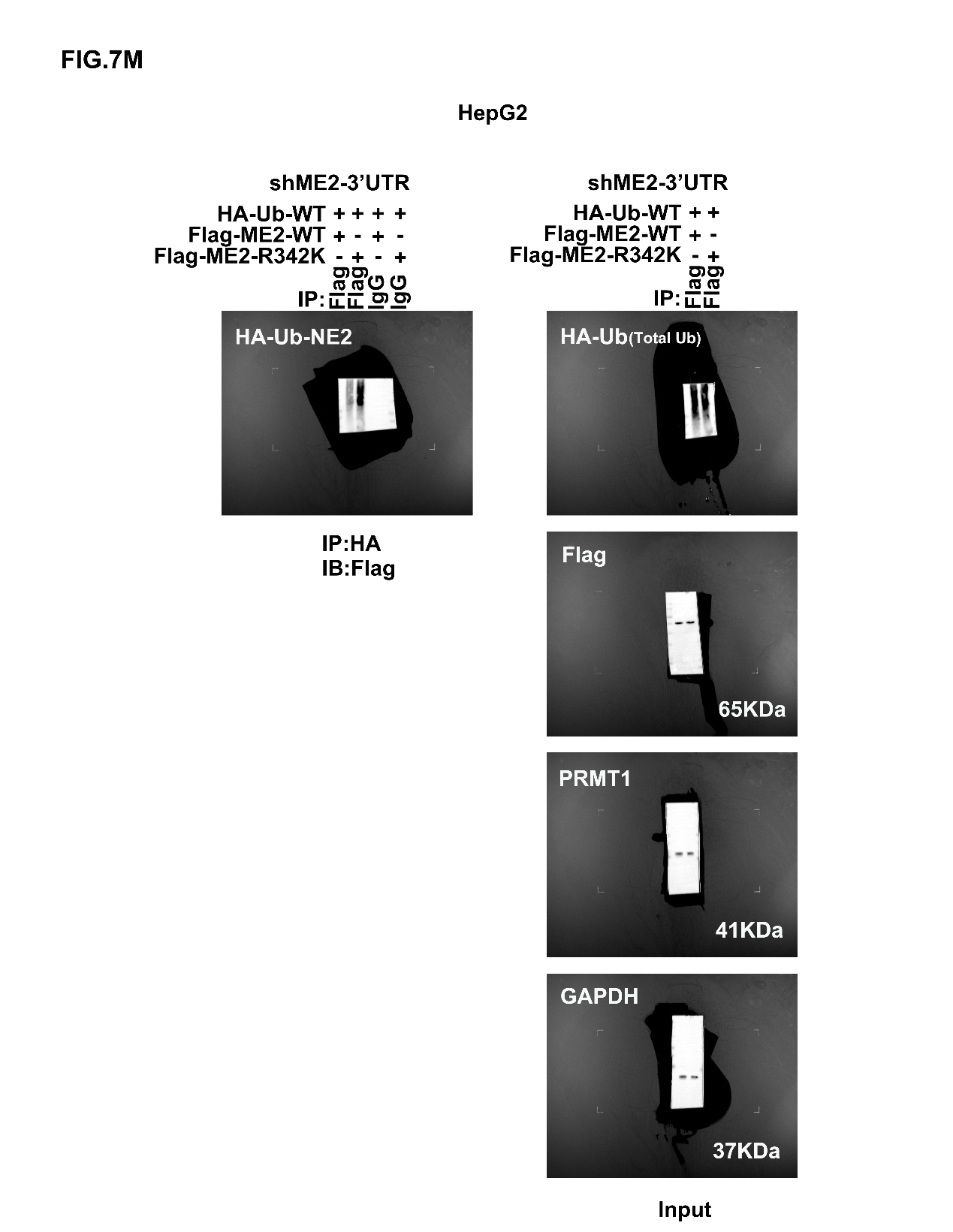


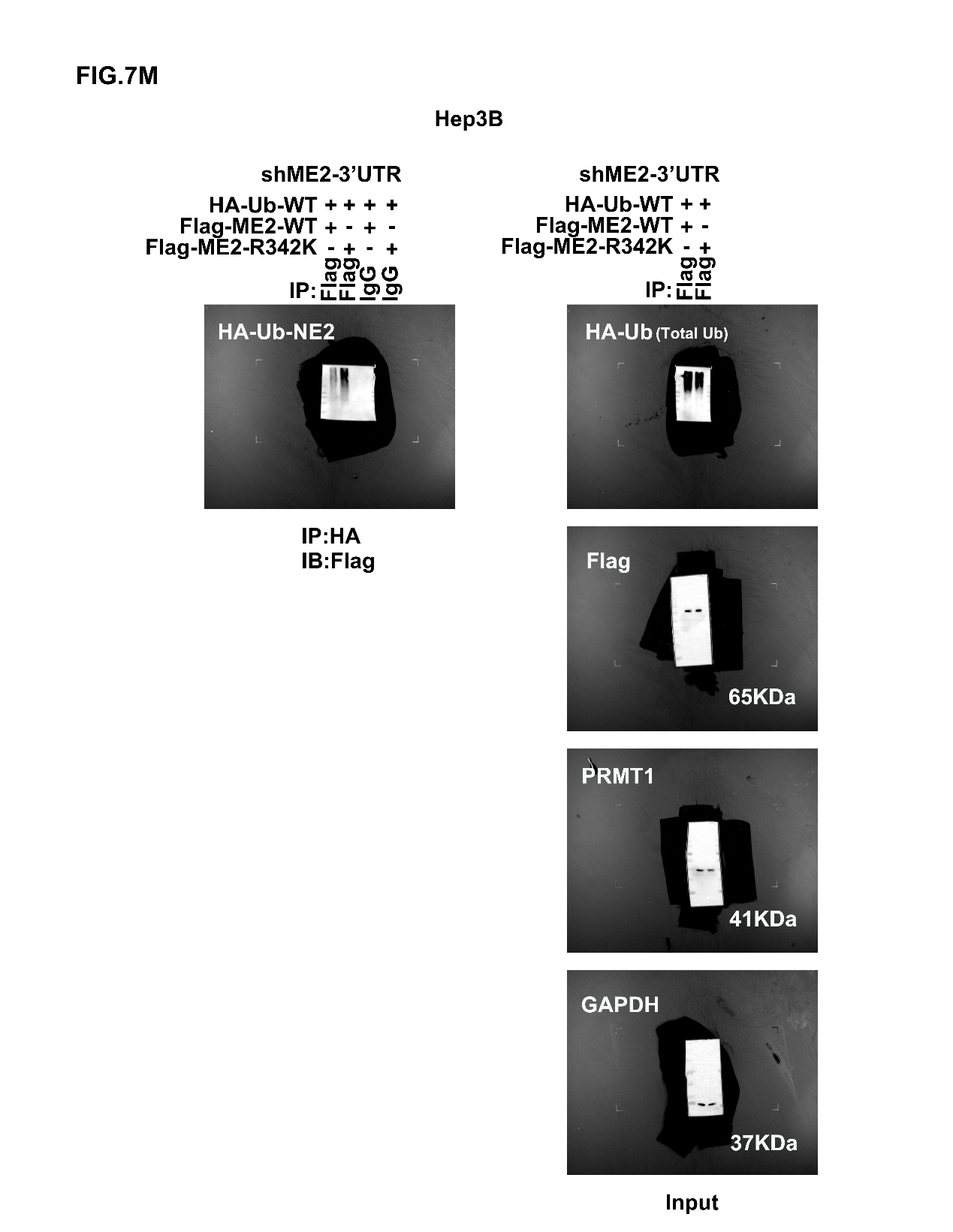

Supplement: Supplementary file 1 — supplementary material [file 41419_2024_7219_MOESM1_ESM.docx]
